# Colorectal cancer-associated *Streptococcus gallolyticus*: a hidden diversity expose 

**DOI:** 10.1128/jb.00230-25

**Published:** 2025-08-14

**Authors:** Bruno Périchon, Thomas Cokelaer, Wooi Keong Teh, Laurence du Merle, Laurence Ma, Marie Touchon, Alexandra Doloy, Claire Poyart, Michael Givskov, Patrick Trieu-Cuot, Shaynoor Dramsi

**Affiliations:** 1 Institut Pasteur, Université Paris Cité, Unité de Biologie des Bactéries Pathogènes à Gram-Positif 555089, Paris, France; 2 Institut Pasteur, Université Paris Cité, Microbial Evolutionary Genomics, CNRS UMR3525 27058, Paris, France; 3 Institut Pasteur, Université Paris Cité, INSERM U1201, Unité de Parasitologie moléculaire et Signalisation 27058, Paris, France; 4 Institut Pasteur, Université Paris Cité, Bioinformatics and Biostatistics Hub 27058, Paris, France; 5 Singapore Centre for Environmental Life Sciences Engineering, Nanyang Technological University 54761, Singapore, Singapore; 6 Institut Pasteur, Plateforme technologique Biomics 27058, Paris, France; 7 Assistance Publique Hôpitaux de Paris, Hôpitaux universitaires de Paris Centre-site Cochin Service de Bacteriologie, Centre National de Référence des Streptocoques, Paris, France; 8 Departement of Immunology and Microbiology, Faculty of Health and Medical Sciences, Costerton Biofilm Center, University of Copenhagen 4321, Copenhagen, Denmark; 9 Université Paris Cité, CNRS UMR8104, Inserm U1016, Institut Cochin, Bacterial Pathogenesis and Innate Immune Signaling Team, Paris, France; University of Illinois Chicago, Chicago, Illinois, USA

**Keywords:** *Streptococcus gallolyticus *subsp. *gallolyticus*, genome, antibiotic resistance genes, *Streptococcus gallolyticus *subsp. *macedonicus*, colorectal cancer

## Abstract

*Streptococcus gallolyticus* subsp. *gallolyticus* (*SGG*) is a bacterial pathogen implicated in bacteremia and endocarditis and is often associated with colon tumors in elderly individuals. The development of colorectal cancer (CRC) has been linked to intestinal dysbiosis, characterized by increased proportions of *SGG* and other intestinal microbes. In this study, we present the complete nucleotide sequence of five novel clinical isolates of *SGG* associated with colorectal cancer, revealing unexpected genetic diversity. Sequencing an additional 30 *SGG* clinical isolates provided a more comprehensive description of this genetic diversity. We did not identify a pathogenicity island specific to CRC-associated *SGG* isolates. Most of these human-derived *SGG* isolates exhibit resistance to multiple antibiotics. Our findings also offer additional insights into multilocus sequence typing (MLST), capsular loci, and pilus organization. Analysis of the repertoire of surface proteins reveals high potential for binding and foraging complex polysaccharides. Finally, comparative genomics with the phylogenetically closest non-pathogenic subspecies *S. gallolyticus* subsp. *macedonicus* confirmed that *SGG* pathogenicity-associated factors mostly rely on a large repertoire of surface proteins involved in host colonization, presence of C5a peptidase to avoid innate immunity, bile salt hydrolase to persist in the gut, and of specific bacteriocin and type VII-dependent effectors to colonize the host colon. Additionally, the presence of extracellular polysaccharides in *SGG* probably helps the bacterium survive in harsher conditions.

**IMPORTANCE:**

*Streptococcus gallolyticus* subsp. *gallolyticus* (*SGG*) was the first intestinal bacterium associated with colorectal cancer. It is now widely accepted that colonic microbiota dysbiosis contributes to oncogenesis, with a higher relative abundance of several potentially pro-carcinogenic bacteria. For example, the oncogenic role of *Escherichia coli pks*+ and enterotoxinogenic *Bacteroides fragilis* in colorectal cancer has been well established, identifying the role of genetic loci encoding toxins. Through the sequencing and analysis of 11 clinical *SGG* isolates from CRC patients and comparisons with non-CRC isolates, we uncovered a significant diversity among CRC-associated strains. Our findings suggest that *SGG* association with CRC is complex and is not linked to a specific strain or pathogenicity island, thus highlighting the opportunistic and versatile nature of *SGG*.

## INTRODUCTION

*Streptococcus gallolyticus* subsp. *gallolyticus* (*SGG*), formerly known as *Streptococcus bovis* biotype I, is a member of the larger *Streptococcus bovis*/*Streptococcus equinus* complex (SBSEC). The group *Streptococcus gallolyticus* includes two additional subspecies, namely subsp. *macedonicus* (*SGM*) and subsp. *pasteurianus* (*SGP*).

*SGG* is a commensal bacterium of the digestive tract of birds and of the rumen of herbivores (1). It is estimated that around 30% of humans harbor this bacterium in their gastrointestinal tract (2). *SGG* is recognized as an opportunistic pathogen, primarily responsible for bacteremia and endocarditis, especially in elderly individuals.

The first whole genome sequence of *S. gallolyticus* subsp. *gallolyticus* strain UCN34 was published in 2010 (3). This clinical isolate was recovered from the blood of a 70-year-old man with endocarditis and a colorectal tumor. Genome analysis of strain UCN34 revealed distinct features and suggested that this subspecies likely acquired genes through horizontal gene transfer from other species present in the rumen flora, such as *Lactobacillus* or *Clostridia* species (3).

*SGM*, a species used in the dairy industry, is the genetically closest relative to *SGG* and is considered non-pathogenic. Comparative genomics of *SGG* and *SGM* allowed the identification of putative virulence genes (4). One example is the *pil1* locus, which encodes the Pil1 pilus. This proteinaceous appendage at the bacterial surface allows *SGG* binding to collagen, bacterial colonization of cardiac valves, and the subsequent development of endocarditis (5).

Of medical interest, *SGG* was the first intestinal bacterium associated with colorectal cancer (CRC) (6). It is now widely accepted that colonic microbiota dysbiosis contributes to oncogenesis, with a higher relative abundance of potentially pro-carcinogenic bacteria, including *Escherichia coli pks*+, toxinogenic *Bacteroides fragilis* (ETBF)*, Fusobacterium nucleatum*, *Parvimonas micra,* and *SGG* (7, 8). The *pks* island is a cluster of genes that encode polyketide synthases and nonribosomal peptide synthetases involved in the production of colibactin, a genotoxin which can directly damage DNA in colonic cells, leading to mutations and potentially initiating cancer (9).

A few studies have experimentally addressed the role of *SGG* in tumorigenesis (10–12). Our research group has shown that the *SGG* UCN34 strain colonizes the murine colon 1,000-fold more effectively in a tumoral environment by outcompeting a phylogenetically close member of the resident murine microbiota (11). More recently, we showed that UCN34 can also accelerate colon tumor development in a murine model by activating several cancer-related signaling pathways in epithelial and stromal colonic cells (12).

Several molecular mechanisms have been proposed to explain the pro-tumoral effect induced by bacteria associated with colorectal cancer. These include (i) the induction of chronic inflammation; (ii) the secretion of specific bacterial factors such as toxins with oncogenic properties; (iii) the formation of mucus-invasive biofilms; (iv) the alteration of host transcription through genetic or epigenetic mechanisms; (v) the bacterial transformation of host metabolites into carcinogens; and (vi) the alteration of host immunity, creating a pro-tumoral environment (13). The oncogenic role of *E. coli pks*+ and enterotoxinogenic *B. fragilis* in colorectal cancer has been well documented (14). Specific genetic loci encoding the oncotoxin colibactin (*clbB, pks+*) and fragylisin also known as *B. fragilis* toxin (*bft*) have been identified within pathogenicity islands (15, 16). Fragilysin, produced by ETBF, is a metalloprotease that can cleave E-cadherin, a protein crucial for maintaining the integrity of epithelial cell junctions, leading to chronic inflammation and c-myc dependent hyperproliferation (17).

Very recently, it was shown that a distinct *Fusobacterium nucleatum* clade dominates the colorectal cancer niche, namely *Fn* subsp. *animalis* clade 2 (18). Similarly, only the phylotype A of *Parvimonas micra* was shown to be associated with colorectal cancer, epigenetically reprogramming human colonocytes (19).

To determine whether unique genetic loci or pathogenicity islands are present in CRC-associated *SGG*, we performed *de novo* sequencing of five novel *SGG* isolates associated with CRC and compared them to 30 other isolates linked to various clinical conditions. Our research shows that *Streptococcus gallolyticus* subsp. *gallolyticus* (*SGG*) isolates linked to colorectal cancer (CRC) are not clonal but rather diverse. We did not identify any genetic cluster specific to the *SGG* associated with CRC, suggesting that the association between *SGG* and CRC is likely multifactorial and largely dependent on the host. Nevertheless, the presence of *SGG* could serve as a potential diagnostic marker for CRC.

## MATERIALS AND METHODS

### Bacterial strains

Thirty-five *SGG* clinical strains isolated from blood samples were obtained from the National Reference Center for Streptococci in France (Table 1). They were grown at 37°C without shaking in Todd-Hewitt broth for genomic DNA isolation.

**TABLE 1:** Characteristics of whole-genome sequences of *Streptococcus gallolyticus* subsp. *gallolyticus^a^^,b,c^*

Strain	Diagnostic	Size (bp)	Number of contigs	Number of coding sequences	G + C%	MLST type
SGG17	IE + polyps	2,419,039	29	2,423	37.66	7
**SGG20**	IE + colon cancer	2,300,175	25	2,295	37.49	91
SGG21	IE	2,256,983	37	2,224	37.46	26
SGG22	IE	2,392,006	27	2,366	37.81	15
***SGG23***	Non-colon cancer	2,421,052	31	2,429	37.66	7
***SGG24***	IE + Non-colon cancer	2,557,573	54	2,574	37.41	11
***SGG25***	IE + Non-colon cancer	2,429,569	38	2,471	37.49	7
***SGG26***	IE + Non-colon cancer	2,412,039	78	2,422	37.5	62
***SGG27***	IE + Non-colon cancer	2,401,851	30	2,416	37.49	11
SGG28	IE	2,490,913	74	2,504	37.51	18
SGG29	IE	2,300,216	23	2,292	37.49	91
**SGG30**	IE + colon cancer	2,345,098	1	2,307	37.61	41
SGG31	NA	2,310,255	19	2,315	37.59	5
SGG32	NA	2,357,582	21	2,359	37.57	5
**SGG33**	IE + colon cancer	2,317,420	29	2,322	37.43	5
**SGG34**	IE + colon cancer	2,312,760	22	2,304	37.57	5
SGG35	IE	2,239,166	26	2,23	37.54	5
SGG36	IE	2,293,095	30	2,31	37.47	5
**SGG37**	IE + colon cancer	2,273,624	1	2,207	37.78	2
SGG38	IE + polyps	2,410,412	43	2,403	37.46	3
SGG39	Polyps	2,667,828	149	2,764	37.27	118
**SGG40**	Spondylodiscitis + colon cancer	2,468,312	2	2,492	37.67	5
**SGG41**	IE + colon cancer	2,202,809	19	2,15	37.6	120
**SGG42**	IE + colon cancer	2,426,743	3	2,422	37.65	116
SGG43	IE + polyps	2,325,599	14	2,335	37.52	109
**SGG44**	IE + colon cancer	2,366,909	1	2,318	37.62	26
SGG46	IE + diverticulosis	2,445,476	43	2,487	37.46	121
***SGG47***	IE + non-colon cancer	2,674,135	100	2,786	37.18	11
SGG48	IE	2,357,783	21	2,36	37.49	7
***SGG49***	IE + non-colon cancer	2,233,030	11	2,199	37.54	2
***SGG50***	IE + non-colon cancer	2,310,202	43	2,329	37.52	43
SGG51	IE	2,398,145	21	2,374	37.8	15
***SGG52***	IE + non-colon cancer	2,375,034	64	2,407	37.47	119
SGG53	IE	2,274,758	19	2,275	37.53	5
SGG54	NA	2,304,878	16	2,287	37.56	2
**UCN34**	IE + colon cancer	2,350,911	1	2,307	37.64	41
**TX20005**	IE + ‘suspected colon cancer’	2,258,003	1	2,224	37.73	117
ATCC 43143	NA	2,362,241	1	2,246	37.52	12
BAA 2069	NA	2,356,444	1	2,309	37.65	6
DSM 16831	Non-human (Koala)	2,492,900	1	2,464	37.7	1
**Mean**		**2,372,680**		**2,368**	**37.55**	

^*a*^ IE, infective endocarditis.

^*b*^ NA, not avalaible.

^*c*^ In bold : Cancer-associated strains. In bold and italics : No cancer-associated strains. In gray : a non-human strain.

### Antimicrobial susceptibility tests

Antimicrobial activity was tested by the agar disk-diffusion assay. A standardized inoculum of *SGG* strain was plated onto the surface of Mueller-Hinton agar containing 5% horse blood. Filter paper disk impregnated with antimicrobial agent is placed on the agar and plates were incubated overnight at 37°C. The isolates were tested for susceptibility to penicillin, ampicillin, tetracycline, gentamicin, kanamycin, erythromycin, lincomycin, pristinamycin, rifampicin, vancomycin, teicoplanin, levofloxacin, linezolid, sulfamethoxazole/trimethoprim, norfloxacin, and oxacillin. The minimum inhibitory concentration (MIC) of tetracycline was determined using the E-test gradient strip method (Biomerieux, Marcy l’Etoile, France).

### Genomic DNA isolation, sequencing, and assembly

Streptococcal strains were grown in Todd-Hewitt broth and incubated overnight at 37°C without agitation. The genomic DNA was extracted using DNAeasy blood and tissue kit (Qiagen) according to the manufacturer’s instruction.

Genome sequencing of five *SGG* strains associated with colon cancer (SGG30, SGG37, SGG40, SGG42, and SGG44) was performed in-house using a PacBio Sequel I instrument. Libraries were prepared according to the Pacbio Protocol for multiplex SMRT sequencing with v3 chemistry, utilizing the SMRTbell Express Template kit 2.0 and barcoded adapters kit. Assemblies were performed using the LORA pipeline v1.0.0 from Sequana (20). The LORA pipeline was run with default parameters and reproducible containers from the Damona project (https://github.com/cokelaer/damona), with Canu (21) as the assembler. Circularization was performed (22), and coverage homogeneity was assessed using Sequana Coverage software for quality control. The final scaffolds consisted of a single circularized contig for SGG30, SGG37, and SGG44, while the scaffolds for SGG40 and SGG42 were built from two and three contigs, respectively. In these cases, gaps between contigs were filled with Ns.

The sequencing data and scaffold for SGG30, SGG37, SGG40, SGG42, and SGG44 generated in this study are available in the European Nucleotide Archive (ENA) under the project accession number PRJEB88997. The resulting sequences were annotated with the RAST server (23).

Genome sequencing of an additional 30 *SGG* strains was performed at the Singapore Center for Environmental Life Sciences Engineering to at least a coverage of 100× on the Illumina Miseq platform (reagent kit v3, 2 × 300 bp). The paired-end sequencing reads were trimmed and assembled *de novo* into contigs on the CLC Genomics Workbench 10.0 (Qiagen). The resulting sequences were annotated with the RAST server (23). Raw sequencing reads used in this study are available on NCBI under accession number PRJNA762634 (24).

### Comparative genomic analysis

Genomic analysis was performed on our collection of 35 *SGG* and 1 *SGM*. In addition, we used the complete genome sequence of five *SGG* and two *SGM* available in the NCBI database, namely UCN34 (GenBank accession no FN597254) (3), TX20005 (GenBank accession no NZ_CP077423) (25), ATCC 43143 (GenBank accession no NC_017576) (26), ATCC BAA-2069 (GenBank accession no NC_015215) (27), and DSM16831 (GenBank accession no NZ_CP018822) (28), *SGM* ACA-DC198 (GenBank accession no HE613569) (29), and 679 (GenBank accession no GCA_900094105) (30).

The pangenome of the 35 *SGG* and 1 *SGM* was inferred using PanACoTA v1.4.1 (31), which clustered proteins with ≥80% sequence identity via single-linkage using MMseqs2 v15-6f452, resulting in 7,054 protein families. A gene family was classified as persistent if it was present as a single copy in at least 90% of the genomes (≥39 out of 43), yielding 1,663 persistent families. Each persistent family was aligned at the protein level using MAFFT v7.49 through PanACoTA. Alignments were subsequently back-translated to nucleotide sequences by replacing each amino acid with its original codon, a strategy that preserves nucleotide-level phylogenetic signal—particularly relevant for species-level polymorphism analyzes. The resulting nucleotide alignments were concatenated into a single alignment of 1,541,250 base pairs. A maximum-likelihood phylogenetic tree was then reconstructed from this concatenated alignment using IQ-TREE v2.2.0. Model selection was conducted with ModelFinder, which identified GTR + F + I + G4 as the best-fit substitution model based on the Bayesian Information Criterion (BIC). Branch support was assessed using 1,000 ultrafast bootstrap replicates to evaluate node robustness.

Jspecies web server (32) was used to determine the genetic diversity among the genomes by calculating the average nucleotide identity (ANI) based on ANIm (MUMer) and the OrthoVenn3 web server (https://orthovenn3.bioinfotoolkits.net/) was used to identify orthologous gene clusters in the genomes (default parameters: E-value 1 × 10^–5^, inflation value 1.5).

The core and pan-genome were defined using SPINE/AGEnt/ClustAGE web server (33).

MLST analysis based on the divergence of sequence of seven housekeeping genes (*aroE, glgB*, *nifS*, *p20*, *tkt*, *trpD*, and *uvrA*) was performed *in silico* using the scheme developed for *SGG* by Dumke et al. (34). The MLST type of *SGG* strains was determined using the public databases for molecular typing and microbial genome diversity (pubMLST.org) (35).

Lipoprotein signal peptides, putative LPxTG proteins, and genes putatively involved in virulence were predicted by using SignalP-6.0 (36), PSORTb-3.0 (37), and the virulence factor database (VFDB, https://www.mgc.ac.cn/cgi-bin/VFs/v5/main.cgi) (38), respectively.

Antibiotic resistance genes were identified with the Resistance Gene identifier (RGI) program from the comprehensive antibiotic-resistance database (CARD) (https://card.mcmaster.ca/analyze/rgi) (39) and the putative genomic islands by using the IslandViewer four database (http://www.pathogenomics.sfu.ca/islandviewer/) (40).

## RESULTS AND DISCUSSION

### Generalities and comparative genomics

*De novo* sequencing of five clinical strains of *Streptococcus gallolyticus* subsp. *gallolyticus* (*SGG*) using PacBio technology revealed an unexpected genetic diversity. These strains isolated in France from the blood of patients with colon cancer and designated SGG30, SGG37, SGG40, SGG42, and SGG44 were analyzed alongside two previously sequenced *SGG* strains, UCN34 (Accession number, NC_013798) and TX20005 (Accession number, NZ_CP077423). This latter strain was isolated from the blood of an endocarditis patient in a U.S. hospital (41). The general characteristics of these seven *SGG* strains are summarized in Table 1. The circular chromosome size ranged from 2,258,003 bp to 2,468,312 bp, with 2,207 to 2,492 open reading frames (ORFs). The G + C content remained consistent at 37.7%, across all seven strains. Each strain contained 18 predicted rRNAs, while the number of predicted tRNAs ranged from 70 to 72. Annotation using the RAST server indicated that approximately 23% of coding sequences (CDS) were classified as “hypothetical proteins.”

Additionally, whole-genome short-read sequencing of 30 clinical *SGG* strains recovered from human blood samples in France was performed by Illumina technology and *de novo* assembly. The number of contigs ranged from 11 to 149, while the estimated circular chromosome size varied between 2,202,809 to 2,674,135 bp (Table 1; Fig. S1A). The number of CDS ranged from 2,150 to 2,786 (mean, 2,368) (Fig. S1B). The average G + C% was 37.5% (37.2%–37.8%) (Fig. S1C). Among the 30 additional *SGG* isolates of our collection, four (SGG20, SGG33, SGG34, and SGG41) were isolated from patients exhibiting colon cancer at the colonoscopy examination, while nine (SGG23, SGG24, SGG25, SGG26, SGG27, SGG47, SGG49, SGG50, and SGG52) were from patients without colon cancer at the time of strain isolation (Table 1).

**Fig 1:**
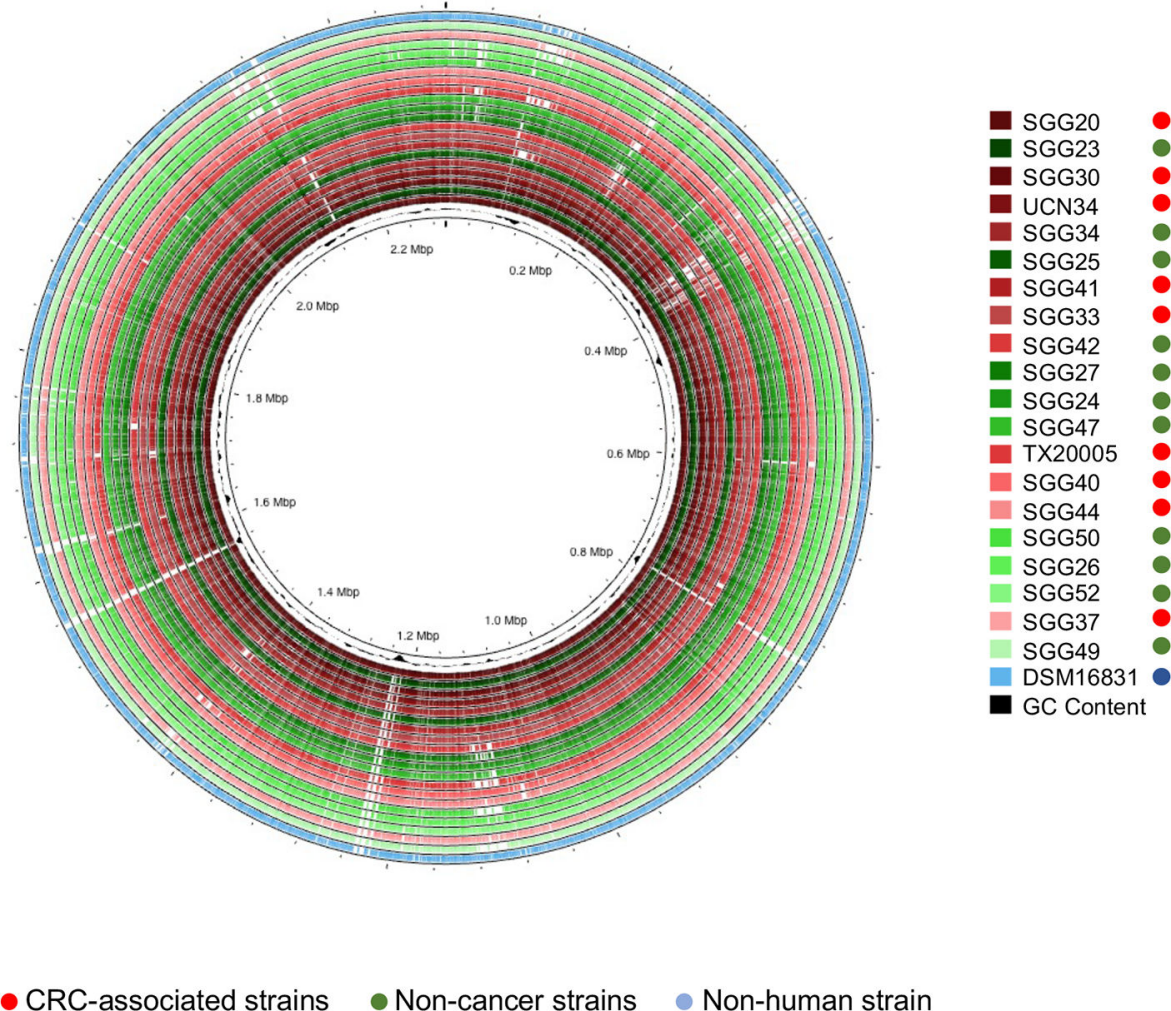
Circular representation of *SGG* genomes associated with colorectal cancer (in red) or non-cancer-associated (in green), including a non-human *SGG* isolate DSM16831 (in blue) for comparison. The innermost circle (in black) shows the G + C% content plot. The genomes were classified based on their level of nucleotide identity, the sense of ranking (highest to lowest identity) being from the inner circle to outer circle.

For further analysis, three additional genomes from the NCBI database were included: ATCC 43143, ATCC BAA-2069, and DSM16831 (Table 1). Notably, DSM16831 is the only non-human *SGG* strain, isolated from koala feces in Australia. A whole-genome circular comparative map was generated for cancer-associated isolates (UCN34, TX20005, SGG30, SGG37, SGG40, SGG42, SGG44, SGG20, SGG33, SGG34, and SGG41) colored in red and non-cancer-associated isolates (SGG23, SGG24, SGG25, SGG26, SGG27, SGG47, SGG49, SGG50, and SGG52) colored in green with DSM 16831 colored in blue also included for reference (Fig. 1). In this figure, genomes are organized according to their degree of identity (average nucleotide identity). This representation clearly shows the intertwining of red and green genomes, indicating that *SGG* isolates associated with CRC do not segregate from *SGG* belonging to the non-cancer group.

Average nucleotide identity (ANI) analysis revealed that within the cancer-associated group, the SGG37 and SGG40 were the most genetically distant strains (ANI, 98.97%), while UCN34 and SGG30 were the most closely related (ANI, 99.99%) (Table S1). In the non-cancer group, SGG47 and SGG49 were the most distant (ANI, 98.93%), whereas SGG27 and SGG47 were the closest (ANI, 99.99%). The average ANI within the cancer and non-cancer group was 99.41% and 99.24%, respectively. Comparisons with the non-human strain DSM16831 showed an average ANI of 99.03% with the cancer-associated group and 98.98% with the non-cancer group (Table S1).

### Phylogenetic trees

To assess the genetic diversity of *SGG* isolates, Multi Locus Sequence Typing (MLST), as established by Dumke et al. (34), was employed analyzing seven housekeeping genes (*aroE*, *glgB*, *nifS*, *p20*, *tkt*, *trpD,* and *uvrA*). The number of different alleles identified for each gene was as follows: *aroE* (8), *glgB* (8), *nifS* (7), *p20* (10), *tkt* (7), *trpD* (9), and *uvrA* (10). In total, 22 sequence types (STs) were detected among the 40 *SGG* strains, indicating high genetic diversity (Table 1). The most common STs were ST5 (8/40, 20%), ST7 (4/40, 10%), ST2 (3/40, 7.5%), ST11 (3/40, 7.5%), ST41 (3/40, 7.5%), and ST91 (3/40, 7.5%). Additionally, six novel STs were identified: ST116 (SGG42), ST117 (TX20005), ST118 (SGG39), ST119 (SGG52), ST120 (SGG41), and ST121 (SGG46). Fourteen STs were unique (14/40, 35%). No correlation was observed between MLST types and cancer association. Among the 11 *SGG* isolates from cancer patients, eight distinct STs were represented, while the 10 strains from non-cancer patients were distributed across seven STs, respectively (Table 1).

**Fig 2:**
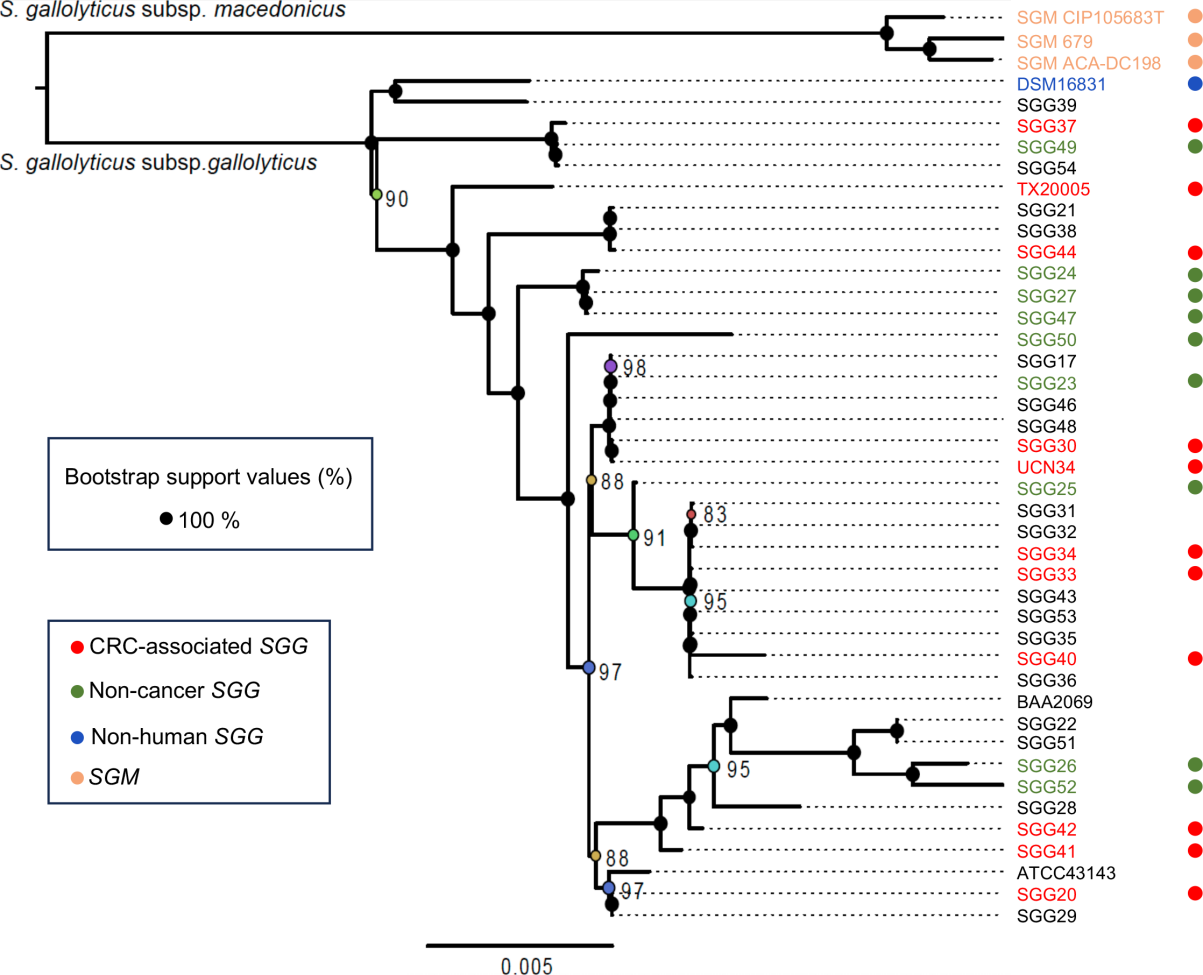
Maximum-likelihood phylogeny of 43 genomes based on 1,663 persistent gene families. The pangenome was built with MMseqs2 (≥80% identity, via PanACoTA), yielding 7,054 families. Persistent families (single-copy in ≥90% of genomes) were aligned with MAFFT, back-translated to nucleotides, and concatenated (1,541,250 bp). The tree was inferred with IQ-TREE (GTR + F + I + G4 model, selected by ModelFinder), with 1,000 ultrafast bootstrap replicates. Only bootstrap support values lower than 100% are shown at the nodes (based on 1,000 replicates). Most nodes are strongly supported, indicating robust phylogenetic relationships.

A phylogenetic tree was generated using the whole genome sequences of the 40 *SGG* and 3 *SGM* strains (Fig. 2). The distribution of strains revealed that those isolated from blood of patients with tumors were not clonal but highly diverse.

### Determination of the core- and pan-genomes

The core genome consists of sequences conserved across all strains, while the pan-genome comprises the core, the accessory genome (genes present in at least two genomes), and strain- or species-specific genes (found in only one genome). The core- and pan-genomes of *SGG* strains from both cancer- and non-cancer groups were determined separately using the “Spine and Agent” web server (http://spineagent.fsm.northwestern.edu/index_age.html) (33). The total number of predicted coding sequences (CDS) and the average GC content for each group are summarized in Table 2. The core genome size for strains associated with cancer (*n* = 11) and those not associated with cancer (*n* = 9) was 1.64 and 1.63 Mbp, respectively, accounting for an average of 80.1% and 75.2% of the total genome (Table 2).

**TABLE 2:** *SGG* core- and pan-genome characteristics

	Cancer-associated group (*n* = 11)		Non cancer-associated group (*n* = 9)	
	Core	Pangenome	Core	Pangenome
Size (nucleotides)	1,646,264	2,950,941	1,629,376	3,181,519
% of genome	80.1 (76.2-84)		75.2 (68.1-81.1)	
G + C %	38.8	37.5	38.8	37.4
Nb pred. CDS	1,856	3,727	1,833	4,137
Gene length (bp)	887	792	889	769

The size of the pan-genome depends on the number of strains analyzed. For the cancer-associated and non-cancer-associated groups, the pan-genome sizes were 2.95 and 3.18 Mbp, respectively (Table 2).

**Fig 3:**
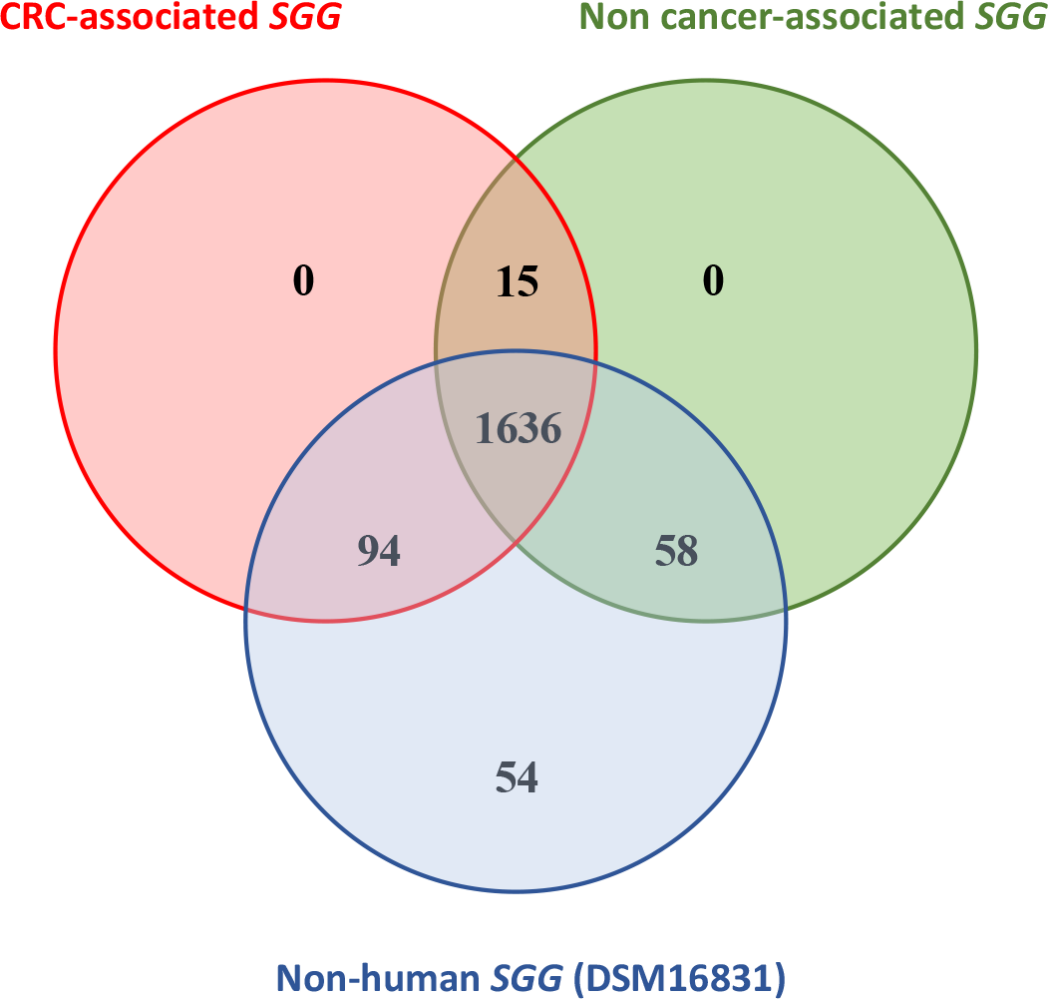
Venn diagram representing common and specific orthologous clusters of *SGG* core genomes from cancer-associated, non-cancer-associated, and non-human strains. The numbers of unique and shared orthologous clusters of each core genome are indicated.

A comparison of the entire genomes of *SGG* isolates from cancer and non-cancer groups did not reveal any specific genes or genetic locus (Fig. 3). However, we recognize that this classification between cancer-associated and non-cancer-associated strains is not so clear. Indeed, it has been reported that colonic neoplasia can develop years after *SGG* IE (42). Thus, it is possible that a patient who did not have a colon tumor at the time of strain isolation may continue to be colonized by *SGG* in the gut and develop tumors in subsequent years. Therefore, comparison of human *SGG* isolates to the non-human DSM16831 could be more relevant. Or, as pinpointed by a reviewer, core and pan-genome of all *SGG* human isolates with infective endocarditis can be compared with core and pan-genomes of the two other subspecies pasteurianus (*SGP*) and macedonicus (*SGM*) never associated with colon cancer.

### Genomic islands

Horizontal gene transfer (HGT) enables the acquisition of new gene in both prokaryotic and eukaryotic genomes. Genomic islands (GI) are regions acquired through HGT that may harbor genes implicated in metabolism, pathogenicity, or antibiotic resistance.

Putative genomic islands were identified using the IslandViewer4 web tool (40). Among the *SGG* strains associated with cancer, the number of GIs ranged from 4 to 15, with GI-to-genome size ratios spanning from 4.5% to 11.6% (Table 3).

**TABLE 3:** Determination of genomic islands in cancer-associated *SGG*, non-cancer-associated SGG, non-human SGG, and in S*. gallolyticus* subps. *macedonicus* (SGM)^*a*^

	Cancer-associated SGG											
	UCN34	TX20005	SGG20	SGG30	SGG37	SGG40	SGG42	SGG44	SGG33	SGG34	SGG41	Mean
No of GIs	8	13	7	8	11	15	8	9	9	10	4	9.9
GI total length (kb)	171	186	129	171	185	287	174	243	169	160	98	193
Largest GI (kb)	36	41	39	36	40	43	53	64	47	46	46	44
Mean length (kb)	21	14	18	21	17	18	22	27	19	16	25	20
Size GI/Size genome (%)	7.4	8.2	5.6	7.4	8.6	11.6	7.1	10.6	7.3	6.9	4.5	8.4

	Non cancer-associated SGG									
	SGG23	SGG24	SGG25	SGG26	SGG27	SGG47	SGG49	SGG50	SGG52	Mean
No of GIs	8	15	9	12	9	15	5	9	14	11
GI total length (kb)	148	265	265	279	217	361	86	231	290	238
Largest GI (kb)	36	47	72	47	62	97	38	43	47	54
Mean length (kb)	18	18	29	23	24	24	17	43	21	24
Size GI/Size genome (%)	6.1	10.4	10.9	11.6	9	13.5	3.8	8.5	12.2	9.8

	Non human SGG	SGM			
	DSM16831	ACA_DC198	679	CIP_105683T	Mean
No of GIs	9	16	15	16	15.7
GI total length (kb)	169	333	261	390	328
Largest GI (kb)	47	95	94	66	85
Mean length (kb)	19	21	17	24	21
Size GI/Size genome (%)	7.3	15.6	12.4	17.6	15.2

^*a*^ Genomic islands were determined by using Island viewer 4 (http://www.pathogenomics.sfu.ca/islandviewer/) (40).

### Antibiotic resistance genes

Like most streptococcal infections, bacteremia caused by *SGG* is treated with amoxicillin or a combination of amoxicillin and gentamicin for 7 days. If endocarditis or other complications are detected, this treatment can be extended for 2–4 weeks. Analysis of the 40 *SGG* genomes using the CARD database revealed that at least one antibiotic resistance gene was present in 85% of the strains (Table S2). Moreover, 82.5% of *SGG* strains carried at least one gene conferring resistance to tetracycline. As suggested for *Streptococcus agalactiae* (43), in which the dominance of *tet(M*) was also noted, resistance to tetracycline is commonly observed in *SGG* likely due to the extensive use of tetracycline in the livestock industry and in human and veterinary medicine.

A gene cluster (*aadE-sat4-aphA-3*), originally described as part of the transposon *Tn*5405 in methicillin-resistant *Staphylococcus aureus* (44), was identified in 17/40 *SGG* strains. This cluster confers resistance to streptomycin (*aadE*), streptothricin (*sat4*), and kanamycin (*aphA-3*), and was often found alongside the *erm(B*) gene, which confers resistance to macrolides, lincosamides, and streptogramins B. Additionally, a *dfrF* gene, involved in trimethoprim resistance, was detected in one strain (SGG28), and a *catA9* gene, which confers resistance to chloramphenicol, was found in two strains (SGG33 and SGG52) (Table S2). Resistance to streptomycin, erythromycin, or chloramphenicol was confirmed in strains harboring *aadE, erm(B*), or *cat* gene, respectively (data not shown).

**Fig 4:**
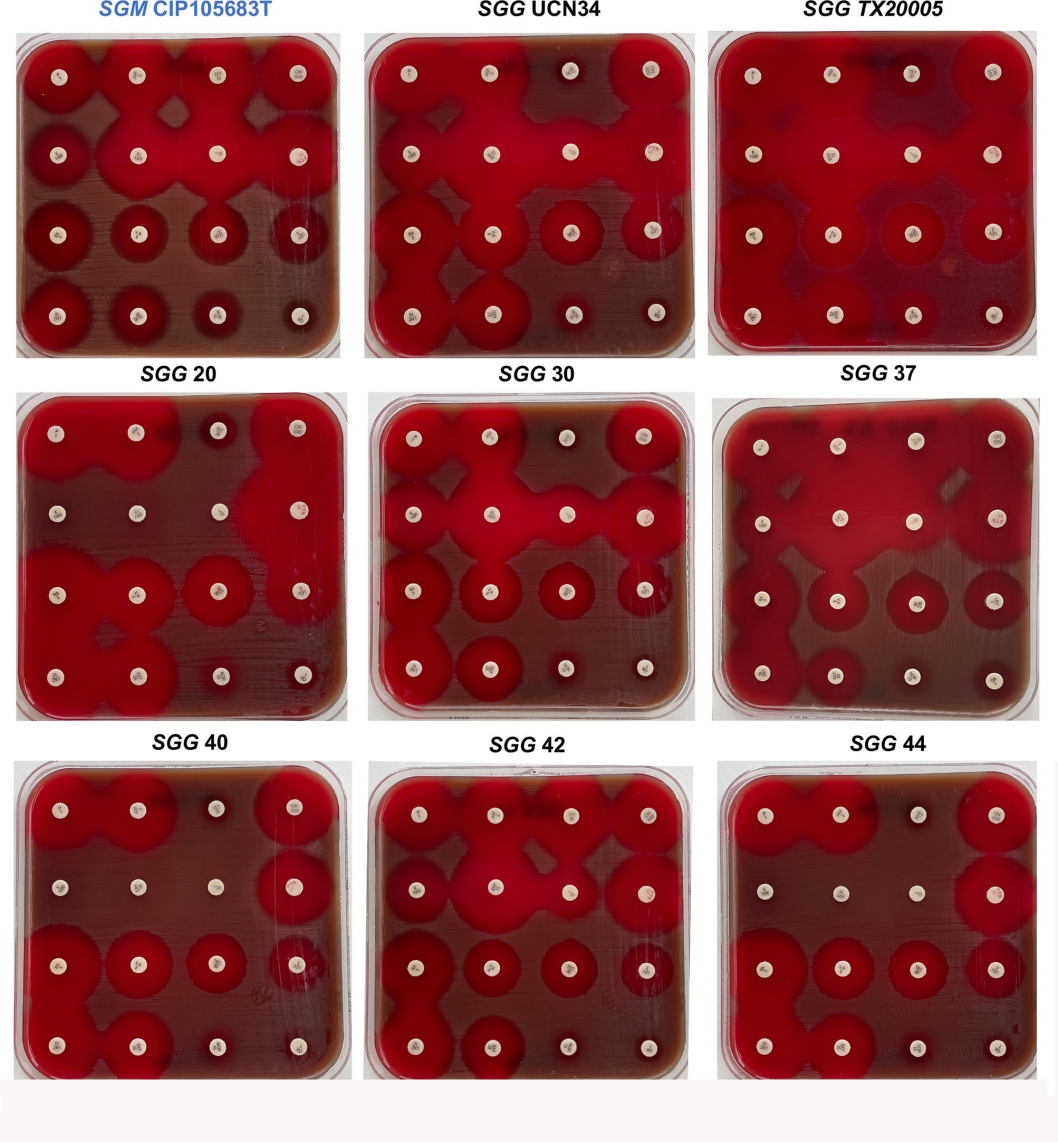
Visual representation of the antimicrobial susceptibility profile of six novel *SGG* isolates associated with colon cancer (SGG20, SGG30, SGG37, SGG40, SGG42, and SGG44). As controls, we included the reference *SGG* strains UCN34 and TX20005 and the non-pathogenic SGM CIP105683T. Each disk is labeled with the abbreviation of the antibiotic tested with the dose indicated below (in µg) Abbreviations: PEN, penicillin; AMP, ampicillin; TET, tetracycline; CN, gentamicin; K, kanamycin; ERY, erythromycin; L, lincomycin; PTN, pristinamycin; RA, rifampicin; VA, vancomycin; TEC, teicoplanin; LEV, levofloxacin; LNZ, linezolid; SXT, sulfamethoxazole/trimethoprim; NOR, norfloxacin; OX, oxacillin.

Antimicrobial resistance testing of all *SGG* strains using disk diffusion assay confirmed that the antibiotic resistance genes detected *in silico* were expressed *in vitro* and that their repertoires could confer different levels of resistance (data not shown). For example, two genes implicated in tetracycline resistance, *tet(M*) and *tet45*, were found in UCN34, while only one gene, *tet(M*), was found in TX20005. *In vitro* tetracycline resistance determination using E-test strips clearly showed that UCN34 was more resistant to tetracycline than TX20005, with measured MIC values of 48 and 16 µg/mL, respectively. Representative antibiograms performed in routine for Streptococci are shown in Fig. 4 for the six sequenced *SGG* strains (SGG20, 30, 37, 40, 42, and 44), the reference strains UCN34 and TX20005, and *SGM* CIP105683T for comparison. SGG40 and SGG44 were resistant to multiple antibiotics, whereas TX20005 and SGG37 were among the most sensitive strains. Like many other Streptococci, most *SGG* isolates were found resistant to norfloxacin and oxacillin.

### Predicted surface proteins

Surface proteins play a crucial role in bacterial invasion, adherence, and interactions with the host immune system and environment. These proteins can be categorized into three groups based on their mode of attachment: LPxTG proteins (anchored at C-terminal end and exceptionally at the N-terminus), hydrophobically bound proteins, and lipoproteins (anchored at the N-terminal end) (45).

#### LPxTG proteins

LPxTG proteins are located on the bacterial surface and anchored to the cell wall by sortase A (46, 47). An analysis of all putative LPxTG detected in our 40 *SGG* strains identified a diverse repertoire of proteins containing both an N-terminal signal peptide and a C-terminal LPxTG motif (Table 4; Table S3). Most of these proteins are predicted to be involved in adherence to host proteins (Table 4). Of the 18 LPxTG proteins detected in the UCN34 strain (3), only six (33%) were present in all genomes of cancer-associated strains (Table 4). Among these, five were also found in non-cancer strains.

**TABLE 4:** Putative LPxTG proteins found in the pangenome of 40 SGG and their presence (+) or absence (−) in cancer-associated - group, non-cancer-associated group, and non-human isolate^*a*^

				Cancer-associated SGG										
ID (a)	Size (AA)	Motif	Putative function	UCN34	TX 20005	SGG 20	SGG 30	SGG 37	SGG 40	SGG 42	SGG 44	SGG 33	SGG 34	SGG 41
1	1,301	LPQTG	Extracellular fructan hydrolase	+	+	+	+	-	+	+	+	+	+	+
2	253	LPETG	Tn-related protein	+	-	+	+	-	+	-	-	-	-	-
3	863	LPTTG	Tn-related protein	+	-	+	+	-	+	-	-	-	-	-
4	1,033	LPTTG	Tn-related protein	+	-	+	+	-	+	-	-	-	-	-
5	750	LPSTG	PDB 4HSQ/A (fimbrial)	+	+	+	+	+	+	-	-	+	+	+
6	560	LPKTG	glucan-binding protein	+	+	+	+	+	+	+	+	+	+	+
7	2,219	LPATG	Pullulanase	+	+	+	+	+	+	+	+	+	+	+
8	505	LPSTG	Pil2 pilin	+	-	+	+	-	+	+	-	+	+	+
9	641	FPSTG	Pil2 adhesin	+	-	+	+	-	+	+	-	+	+	+
10	594	LPKTG	Sel1 TPR-12 -containing protein	+	-	+	+	+	+	+	+	+	+	+
11	300	LPETG	Serine rich protein	+	-	+	+	-	-	+	+	-	-	-
12	759	LPETG	S. mutans adhesin P1-like protein	+	-	-	+	-	+	+	+	+	+	+
13	775	LPSTG	Fimbrial like-protein	+	+	+	+	+	+	+	+	+	+	+
14	478	LPSTG	Pil3 pilin	+	+	+	+	+	+	+	+	+	+	+
15	1,664	LPHTG	Pil3 adhesin	+	+	+	+	+	+	+	+	+	+	+
16	480	LPSTG	Pil1pilin	+	+	+	+	-	+	+	+	+	+	+
17	658	FPNTG	Pil1 adhesin	+	+	+	+	-	+	+	+	+	+	+
18	1,157	LPSTG	CnaB rich- ClfA like protein	-	+	-	-	-	-	-	-	-	-	-
19	652	FPSVG	Pil2 adhesin	-	+	-	-	-	-	-	-	-	-	-
20	480	LPSTG	Pil2 pilin	-	+	-	-	-	-	-	-	-	-	-
21	265	LPHTG	Extracellular protein	-	-	-	-	+	-	-	-	-	-	-
22	1,074	LPMTG	Chromosome partition protein Smc	-	-	-	-	+	-	-	-	-	-	-
23	1,509	LPMTG	Cell surface adhesin	-	-	-	-	+	-	-	-	-	-	-
24	121	LPKTG	LPXTG cell wall anchor domain-containing protein	-	-	+	-	-	+	-	+	-	-	-
25	1,076	LPLTG	Cell surface adhesin	-	-	-	-	-	+	+	-	+	-	-
26	260	LPMTG	Extracellular protein	-	-	-	-	-	+	+	-	-	-	-
27	1,068	LPMTG	Chromosome partition protein Smc	-	-	-	-	-	+	-	-	-	-	-
28	266	LPHTG	Extracellular protein	-	-	-	-	-	+	-	-	-	-	-
29	274	LPMTG	Extracellular protein	-	-	-	-	-	-	+	-	-	-	-
30	1,435	LPNTG	MucBP domain-containing protein	-	-	-	-	-	-	-	+	-	-	-
31	274	LPMTG	Extracellular protein	-	-	-	-	-	-	-	+	-	-	-
32	1,602	LPETG	Spy0128 family protein	-	-	-	-	-	-	-	+	-	-	-
33	269	LPMTG	Extracellular protein	-	-	-	-	-	-	-	+	+	-	-
34	1,631	LPNTG	glucan-binding protein	-	-	-	-	-	-	-	-	-	+	-
35	241	LPQTG	Hypothetical protein	-	-	-	-	-	-	-	-	-	-	-
36	743	LPLTG	Hypothetical protein	-	-	-	-	-	-	-	-	-	-	-
37 (b)	394	LPMTG	Hypothetical protein	-	-	+	-	-	-	-	+	-	-	-
38 (b)	71	LPNTG	Hypothetical protein	-	-	-	-	-	-	-	-	-	-	-
39	1,262	LPSTG	Cna B-type domain-containing protein	-	-	-	-	-	-	-	-	-	-	-
40	207	LPNTG	Spy0128 family protein	-	-	-	-	-	-	-	-	-	-	-
41	1,425	LPSTG	SspB-related isopeptide-forming adhesin	-	-	-	-	-	-	-	-	-	-	-
42	169	LPETG	DNA cytosine methyltransferase	-	-	+	-	-	-	-	+	-	-	-
43	1,472	LPDTG	SpaA isopeptide-forming pilin-related protein	-	-	-	-	+	-	-	-	-	-	-
44	450	LPATG	Alpha-like surface protein	-	-	-	-	-	-	-	-	-	+	-
45	135	LPTTG	SspB-related isopeptide-forming adhesin	-	-	-	-	-	-	-	-	-	-	-
46	811	LPNTG	Hypothetical protein	-	-	-	-	-	-	-	-	-	-	-
47	251	LPNTG	Tn-related protein	-	-	-	-	-	-	-	-	-	-	-
48	1,289	LPATG	Cna B-type domain-containing protein	-	-	-	-	-	-	-	-	-	-	-
49	73	LPKTG	Spy0128 family protein	-	-	-	-	-	-	-	-	-	-	-
50	1,067	LPMTG	Chromosome partition protein Smc	-	-	-	-	-	-	-	-	-	+	-
51	1,519	LPMTG	SspB-related isopeptide-forming adhesin	-	-	-	-	-	+	-	-	-	-	-
52	270	LPHTG	Extracellular protein	-	-	-	-	-	-	-	-	-	+	-
53	760	LPLTG	Hypothetical protein	+	-	-	-	-	+	+	+	+	+	+

				Non cancer-associated SGG								Non-human
ID (a)	Size (AA)	Motif	Putative function	SGG 24	SGG 25	SGG 26	SGG 27	SGG47	SGG 49	SGG 50	SGG 52	DSM 16831
1	1,301	LPQTG	Extracellular fructan hydrolase	+	-	+	+	+	-	+	+	-
2	253	LPETG	Tn-related protein	-	+	-	-	-	-	-	-	-
3	863	LPTTG	Tn-related protein	-	+	-	-	-	-	-	-	-
4	1,033	LPTTG	Tn-related protein	+	+	-	-	-	-	-	-	-
5	750	LPSTG	PDB 4HSQ/A (fimbrial)	+	+	+	+	+	+	+	+	+
6	560	LPKTG	glucan-binding protein	+	+	+	+	+	+	+	+	+
7	2,219	LPATG	Pullulanase	-	+	+	+	+	+	+	+	+
8	505	LPSTG	Pil2 pilin	+	+	-	+	+	-	+	-	+
9	641	FPSTG	Pil2 adhesin	+	+	-	+	+	-	+	-	+
10	594	LPKTG	Sel1 TPR-12 -containing protein	-	+	-	-	-	+	+	-	+
11	300	LPETG	Serine rich protein	-	+	-	-	-	+	+	-	-
12	759	LPETG	S. mutans adhesin P1-like protein	-	+	-	-	-	+	+	-	-
13	775	LPSTG	Fimbrial like-protein	+	-	+	+	+	+	-	-	+
14	478	LPSTG	Pil3 pilin	+	+	+	+	+	+	+	+	+
15	1,664	LPHTG	Pil3 adhesin	+	+	+	+	+	+	+	+	+
16	480	LPSTG	Pil1pilin	+	+	+	+	+	+	+	+	+
17	658	FPNTG	Pil1 adhesin	+	+	+	+	+	+	+	+	+
18	1,157	LPSTG	CnaB rich- ClfA like protein	-	-	-	-	-	-	-	-	-
19	652	FPSVG	Pil2 adhesin	-	-	-	-	-	-	-	-	-
20	480	LPSTG	Pil2 pilin	-	-	-	-	-	-	-	-	-
21	265	LPHTG	Extracellular protein	+	-	+	-	-	-	-	-	-
22	1,074	LPMTG	Chromosome partition protein Smc	-	-	+	-	-	-	-	-	-
23	1,509	LPMTG	Cell surface adhesin	+	-	+	-	-	-	-	-	-
24	121	LPKTG	LPXTG cell wall anchor domain-containing protein	+	+	-	+	+	-	+	-	-
25	1,076	LPLTG	Cell surface adhesin	-	-	+	-	+	-	-	-	-
26	260	LPMTG	Extracellular protein	+	-	+	-	-	-	-	-	-
27	1,068	LPMTG	Chromosome partition protein Smc	-	-	-	-	-	-	-	-	-
28	266	LPHTG	Extracellular protein	-	-	-	-	-	-	-	-	-
29	274	LPMTG	Extracellular protein	-	-	-	-	-	-	-	-	-
30	1,435	LPNTG	MucBP domain-containing protein	-	-	-	-	-	-	-	-	+
31	274	LPMTG	Extracellular protein	-	-	-	-	-	-	-	-	-
32	1,602	LPETG	Spy0128 family protein	-	-	-	-	-	-	-	-	-
33	269	LPMTG	Extracellular protein	-	-	-	-	-	-	-	-	+
34	1,631	LPNTG	glucan-binding protein	+	-	-	-	-	-	-	-	+
35	241	LPQTG	Hypothetical protein	-	-	-	-	-	-	-	-	-
36	743	LPLTG	Hypothetical protein	-	-	-	-	-	-	-	-	-
37 (b)	394	LPMTG	Hypothetical protein	+	-	-	+	+	-	-	-	-
38 (b)	71	LPNTG	Hypothetical protein	-	-	+	-	-	-	-	+	-
39	1,262	LPSTG	Cna B-type domain-containing protein	-	-	+	-	-	-	-	-	-
40	207	LPNTG	Spy0128 family protein	-	-	+	-	-	-	-	+	-
41	1,425	LPSTG	SspB-related isopeptide-forming adhesin	-	-	-	-	+	-	-	-	-
42	169	LPETG	DNA cytosine methyltransferase	-	-	-	-	-	+	-	-	-
43	1,472	LPDTG	SpaA isopeptide-forming pilin-related protein	-	-	-	-	-	+	+	+	+
44	450	LPATG	Alpha-like surface protein	-	-	-	-	-	-	-	+	-
45	135	LPTTG	SspB-related isopeptide-forming adhesin	+	-	+	-	+	-	-	-	-
46	811	LPNTG	Hypothetical protein	-	-	-	-	-	-	-	-	-
47	251	LPNTG	Tn-related protein	-	-	-	-	-	-	-	-	-
48	1,289	LPATG	Cna B-type domain-containing protein	-	-	-	-	-	-	-	-	+
49	73	LPKTG	Spy0128 family protein	-	-	-	-	-	-	-	+	-
50	1,067	LPMTG	Chromosome partition protein Smc	-	-	-	-	-	-	-	+	+
51	1,519	LPMTG	SspB-related isopeptide-forming adhesin	-	-	-	-	-	-	-	-	-
52	270	LPHTG	Extracellular protein	-	-	-	-	-	-	-	+	+
53	760	LPLTG	Hypothetical protein	-	+	-	-	-	+	+	-	-

^*a*^ Sequences of putative LPxTG proteins are shown in Table S3.

Sortase A, a transpeptidase enzyme, anchors covalently LPxTG proteins to bacterial cell wall. In the UCN34 genome, three genes encoding sortase A were identified: the housekeeping Gallo-1127 (present in all 40 *SGG* strains), Gallo_0299 (found in 20/40 strains, 50%), and Gallo_1651 (present in 22/40 strains, 55%). These genes, designated as *srtA*, *srtA_1_*, and *srtA_2_*, are located downstream of genes encoding GyrA, plasmid mobilization proteins, and a “phage tail tip lysozyme,” respectively. Additional sortase genes, *srtA*_3_ (SGG24, SGG42), *srtA*_4_ (SGG47), and *srtA*_5_ (SGG40) were sporadically detected with the number of *srtA* genes varying from 1 to 4 among *SGG* isolates. All *srtA* genes encode potentially functional enzymes with the characteristic catalytic motif TL(V/I)TC at the C-terminal region, a hallmark of the SrtA protein (48). Analysis of the genomic context of these *srtA* genes (Fig. S2) revealed that (i) the *srtA* gene, present in all strains, is consistently downstream of *gyrA*; (ii) additional *srtA* genes (*srtA_1_-srtA_5_*) are consistently found near mobile genetic elements (e.g. transposase, integrase), suggesting that while *srtA* serves as the primary housekeeping enzyme, auxiliary *srtA* genes were acquired through horizontal gene transfer. Blast analysis confirmed that homologs of these additional sortases are found in other *Streptococcus* species, such as *SGM*, *S. gallolyticus* subsp. *pasteurianus* (*SGP*), *Streptococcus infantarius*, and *Streptococcus lutetiensis*.

#### Pili

In Gram-positive bacteria, covalently assembled pili play a key role in adherence and host tissue colonization (49). The UCN34 genome contains three loci involved in pilus biosynthesis: Pil1 (Gallo_2177-2179), Pil2 (Gallo_1570-1568), and Pil3 (Gallo_2038-2040) (3). Pil1 and Pil3 have been extensively studied in UCN34. Pil1 is essential for adhesion to collagen type I and contributes to heart valve colonization and endocarditis in a rat model (5), while Pil3 facilitates adhesion to colonic mucus and colonization of the distal colon in mice (50). Both *pil1* and *pil3* were detected in all 40 *SGG* strains. The *pil1* cluster, located between a *tetR*-like gene, encoding a transcriptional regulator and a trehalose catabolism locus, was conserved in 33 strains. In the remaining seven strains, two additional genes encoding a DNA repair protein and a signal peptidase were inserted upstream of the *pil1* locus (Fig. S3). In these strains, the genes coding for the accessory and major pilins were more distantly related to those in UCN34. Notably, in the CRC-associated SGG37, the genes encoding the major pilin and sortase C in the *pil1* locus were absent. The Pil1 locus is replaced by a large SpaA isopeptide-forming pilin related protein of 1,571 amino acids (Fig. S3). The *pil3* gene cluster was highly conserved in all *SGG* strains, with at least 90% identity across the locus. However, three genes, downstream of *pil3*, encoding a copper transport system, a protease, and a lactate dehydrogenase, were absent in five strains (SGG37, SGG39, SGG49, SGG54, and the non-human isolate DSM 16831) compared with the other 35 other *SGG* strains. Unlike *pil1* and *pil3*, the *pil2* gene cluster was less conserved and was absent in 10/40 (25%) of *SGG* strains. Of note, *pil2* was absent in two CRC-associated isolates (SGG37 and SGG44). When present, it was well conserved, except in TX20005 (Fig. S3). Interestingly, a specific *pil2* locus was detected in the genome of TX20005 and was not found in any of the other 38 strains studied. Overall, these results highlight the critical role of Pil3, which contributes to mucus-binding and host colon colonization by *SGG*. The Pil1 locus, involved in collagen binding and endocarditis, is also well conserved except in a few isolates. Pil2 appears as a species-specific locus whose role remains to be determined.

#### Lipoproteins

Lipoproteins are anchored to the bacterial membrane via an N-terminal lipid moiety attached to a conserved cysteine residue. They are involved in various biological processes, particularly in transport and sugar uptake systems. Compared with LPxTG proteins, lipoproteins exhibit lower variability within *SGG* strains. Using the SignalP 6.0 web server, the number of predicted lipoproteins in *SGG* genomes ranged from 39 to 47. Of the 43 putative lipoproteins identified in UCN34, 34 (79%) of them were conserved across all cancer-associated isolates (Table S4). One lipoprotein, Gallo_1778, was found in all cancer-associated strains except strain TX20005 and was mostly absent (7 out of 9) in non-cancer-associated isolates and the non-human isolate DSM16831. This protein contains a CarG-like motif, which is associated with carbapenem intrinsic resistance factor, and a glycosyl hydrolase motif, suggesting a potential role in bacterial survival and host interactions.

### Biosynthesis of various extracellular polysaccharides in SGG strains

Extracellular polysaccharides play a crucial role in bacterial ability to evade the immune response and to survive in hostile environments. In *SGG* UCN34, three distinct genetic loci have been identified as contributing to polysaccharide biosynthesis.

#### Capsule

**Fig 5:**
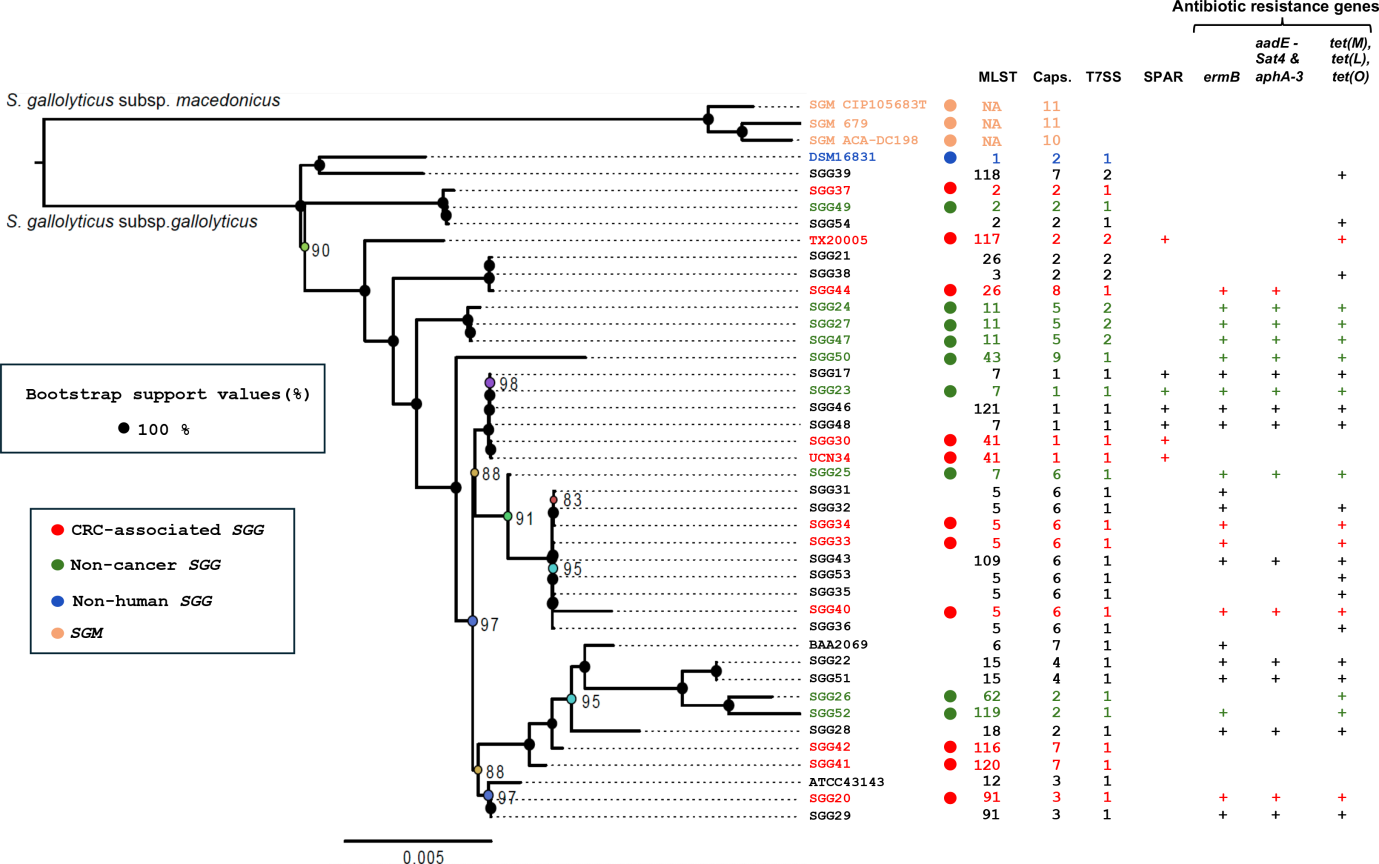
Recapitulative figure showing the diversity of *SGG* isolates associated with CRC in France. Pan-genome-based phylogenetic tree, association or not with CRC at the time of strain isolation, MLST, capsular sequence type (Caps), Type of T7SS arbitrarily defined as one for T7SS_UCN34_-type and two for T7SS_TX20005_-type, presence of SPAR region and antibiotic resistance genes.

A cluster of 12 genes (*cpsA-M*) enables *SGG* UCN34 to synthesize an extracellular capsule (3). Like other extracellular pathogens, this capsule protects *SGG* from phagocytosis and clearance by macrophages (51). Previous studies have shown that the *cps* locus, which encodes the biosynthesis of capsular polysaccharides biosynthesis in *SGG*, consists of two parts: a highly conserved 5′ region and a species- or strain-specific 3′ region (52). The present analysis of 40 *SGG* strains supports this observation. The first five genes (*cpsA-E*), located in the 5′ region of the locus, are highly conserved, while the 3′ region varies among strains, containing between six and eleven genes. Based on nucleotide sequence analysis, we identified nine distinct *cps* cluster types. The capsule type of each *SGG* isolate is indicated in Fig. 5. Our results confirm that CRC-associated *SGG* are diverse and belong to six different capsular types.

#### Hemicellulose

A genetic locus (*gallo_0364-0367*) putatively involved in hemicellulose biosynthesis has been identified in UCN34 (3). This locus consists of four genes encoding a putative diguanylate cyclase, two glycosyltransferases, and a hypothetical transmembrane protein. Analysis of our *SGG* genome collection revealed that this locus is present and highly conserved across the 40 *SGG* strains. However, a few strains exhibit single nucleotide polymorphisms (SNPs) and stop mutations, likely leading to locus inactivation (Fig. S4).

A similar locus is present in *S. infantarius*, *S. lutetiensis*, *S. equinus*, *S. salivarius*, and *S. ruminicola* but is absent in *Streptococcus gallolyticus* subsp. *pasteurianus* (*SGP*). In *SGM* CIP105683T, this locus is present, though the two genes encoding glycosyltransferases appear nonfunctional due to premature stop codons, and the fourth gene encoding the hypothetical transmembrane protein is missing.

#### Glucan

A glucan biosynthesis locus (*gallo_1052-1057*) encoding three glycosyl transferases and associated regulatory proteins has been described in UCN34 (3). These transferases are highly similar to *Streptococcus mutans* GtfA, GtfB, and GtfC, which play key roles in bacterial adherence to tooth surfaces and biofilm formation (3).

Analysis of the 40 *SGG* genomes revealed that this locus is present in all strains. In most isolates (62.5%), the genetic organization is identical to that of UCN34 (Fig. S5). However, variations were observed in some strains: four strains (SGG20, SGG29, SGG42, and ATCC43143; 10%) lacked the first regulatory gene. Ten strains (25%) were missing both the first glycosyltransferase gene and its regulatory gene. Two strains (SGG31 and SGG36) contained a premature stop codon, leading to a truncated glycosyltransferase. One strain (SGG24) lacked the two last genes of the cluster (Fig. S5).

### Type VII secretion system

The type VII secretion system (T7SS) is a specialized protein secretion system originally discovered in Actinobacteria like Mycobacteria. It plays a crucial role in the virulence and survival of these bacteria by secreting proteins across the bacterial membrane. The T7SSb nomenclature specifically refers to the second type VII secretion system found in Bacteroides (ex-Firmicutes), which are Gram-positive bacteria. Canonical effectors secreted by T7SS are WXG- and LXG-containing proteins. A locus encoding a type VII secretion system (T7SS) has been identified in *SGG* strains TX20005 (25) and UCN34 (24). In the TX20005 strain, the T7SS appears to play a role in colon colonization and the development of colon tumors (25).

While the 5′ region of the T7SS locus, which encodes the structural components of the secretion apparatus, is relatively conserved between the two strains, the gene cluster located downstream of *essC,* which encodes putative effectors, shows significant differences (24).

Analysis of 38 additional *SGG* genomes revealed that T7SS associated with UCN34 (T7SS_UCN34_-type) is more commonly found than the T7SS associated with TX20005 (T7SS_TX20005_-type) (32 vs 8 strains, 80% vs 20%). Among the colorectal cancer (CRC) group, 9 out of 11 strains harbor the T7SS_UCN34_-type, compared with 7 out of 10 in the non-cancer group. The results are summarized in Fig. 5.

### TX20005 pathogenicity locus (*sparA-L*)

A recent study identified a chromosomal locus in *SGG* cancer-associated strain TX20005, designated the *SGG* pathogenicity-associated region (SPAR), which consists of 12 genes (JGX27_RS05685 to JGX_RS05740). This locus has been implicated in the adherence of strain TX20005 to colonic cells and its ability to stimulate CRC cell proliferation (53). It was observed that the ∆*spar* mutant phenocopies the ∆*esx* mutant (T7SS knock-out) in TX20005. It was proposed that this could be due to the presence of a putative transcriptional activator (JGX27_RS05710) of the T7SS system in the SPAR locus. An analysis of the 40 *SGG* genomes revealed that the entire locus was present in only 10 genomes (25%). Specifically, it was detected in 3 out of 11 (27.2%) *SGG* isolates associated with cancer and in just 1 out of 11 (9.1%) isolates from the non-cancer group (Fig. 5). These findings suggest that the SPAR locus may have a more limited role in tumor development than previously thought (53). Very interestingly, the only gene of the SPAR locus conserved in all 40 *SGG* is the putative transcriptional activator (JGX27_RS05710) described above. These results demonstrate the importance of studying several clinical strains and the role of National Reference Centers in providing such contemporary circulating strains.

### Virulence factors

The presence of putative virulence genes in the *SGG* genomes was investigated using the VFDB database. Genes associated with adherence, including fibronectin-binding protein (*fbp*), glucosyltransferases (*gtf*), sortases (*srt*), plasmin receptor protein (*plr*), or lipoprotein rotamase A (*slrA*), were identified in *SGG* (Table S5). Additionally, a few proteases, such as C3-degrading protease, C5a peptidase, and HtrA serine protease, were detected, which may help *SGG* to evade the host immune system. The prevalence of these potential virulence factors is summarized in Table S5.

Bile salt hydrolase (BSH), also known as choloylglycine hydrolase, encoded by the *bsh* gene, is commonly found in many bacterial species, especially those present in the gastrointestinal microbiota, such as *Lactobacillus*, *Bifidobacteria*, *Clostridia*, and *Enterococci*. The *bsh* gene is strictly conserved in all *SGG* strains. The potential role of these virulence and resistance factors in the pathogenicity of *SGG* warrants further investigation, particularly in the context of host-pathogen interactions.

### Comparison S*. gallolyticus* subsp. *gallolyticus* vs S*. gallolyticus* subsp. *macedonicus*

*S. gallolyticus subsp. macedonicus* (*SGM*) is a thermophilic, homofermentative, dairy *Streptococcus* that is phylogenetically very close to *SGG*. Unlike *SGG* and *SGP*, *SGM* is considered non-pathogenic (54), which is reflected by a reduced number of pathogenicity-related genes compared with *SGG* (4). Previous sequencing of *SGM* CIP 105683T (55), a type strain from the Institut Pasteur Collection, was used to perform detailed genomic analysis including two others publicly available *SGM* genomes: ACA-DC 198 (Accession number, NC_016749) and 679 (Accession number, GCA_900094105). The general features of these strains are presented in Table 5.

**TABLE 5:** Characteristics of whole-genome sequences of three S*. gallolyticus* subsp. *macedonicus* (*SGM*)

Strain	Acc. number	Size (bp)	G + C content	ORFs	Coding %
ACA-DC198	NC_016749	2,130,034	37.6	2,248	86.2
679	GCA_900094105	2,107,416	37.5	2,277	86.3
CIP_105683T	PRJNA940176	2,210,410	37.7	2,372	85.7

The core genome of *SGM* subspecies was determined from these three strains. Next, a comparison of *SGM* and *SGG* core genomes revealed that 117 proteins, belonging to 30 different orthologous clusters, were specific to the *SGM* core genome (Fig. S6). Our findings support the data reported by Papadimitriou et al. (4) regarding the absence of several virulence factors in *SGM* compared to *SGG* (Table S4).

*SGM* ACA-DC 198 produces two food-grade lantibiotics, macedocin and macedovicin. Macedocin is putatively encoded by two genes (SMA_1380 and SMA_1381) (56) within a 10-gene cluster, whereas macedovicin is encoded by a single gene (SMA_1409) (57). The gene cluster responsible for macedocin biosynthesis was also detected in *SGM* 679 and *SGM* CIP 105683T, but the macedovicin gene was found only in ACA-DC 198. In sharp contrast, all 40 *SGG* isolates harbor the genetic cluster encoding the class IIb bacteriocin named gallocin A, which enables *SGG* to outcompete *Enterococcus faecalis*, a resident member of the host microbiota in tumoral conditions (11).

A large LPxTG protein of 7,961 amino acids (SMA_1990 in the ACA-DC 198 genome) was identified in *SGM*_ACA-DC 198. This protein contains a YSIRK signal peptide motif at the N-terminus, five Flg-new (Pfam 09479) domains, and 48 cadherin repeat-like (cI46864; Pfam 05345) domains at the C-terminus. The Flg-new domain, found in intestinal archaea such as *Methanomassiliicoccales*, exhibits structural similarity with mucus binding domains. Interestingly, the Pil3 adhesin (Gallo_2040), which mediates mucus binding, also contains four Flg-new domains (Fig. S7). The same LPxTG protein was found in *SGM* CIP 105683T, but with only 28 cadherin repeat-like domains, whereas it was absent in SGM_679. SMA_1990 homologs were identified in various *Streptococcus* species, including *S. infantarius, S. thermophilus, S. iniae*, *S. parauberis*, *S. suis*, *S. dysgalactiae*, as well as in *Lactococcus* species such as *L. lactis* and *L. raffinolactis*. However, it was absent in the two subsp. *SGG* and *SGP*.

Unlike *SGG*, *SGM* lacked the gene clusters encoding the type VII secretion system (T7SS) and glucan biosynthesis. The absence of T7SS, a system often associated with bacterial ability to compete with closely related bacteria and colonize the gut microbiota, may further explain the non-pathogenic nature of *SGM*. Similarly, the lack of glucan biosynthesis genes, which contribute to biofilm formation and adhesion in pathogenic *Streptococcus* species, suggests that *SGM* has a reduced ability to establish persistent infections. Moreover, the gene encoding bile salt hydrolase is probably nonfunctional in *SGM* due to a premature stop codon in the coding sequence. These genetic differences highlight the evolutionary divergence between *SGM* and its pathogenic counterpart, *SGG*, reinforcing the hypothesis that *SGM* is adapted for a non-pathogenic, dairy-associated lifestyle rather than a virulent, host-associated one.

### Concluding remarks and perspectives

The analysis of 11 clinical *Streptococcus gallolyticus* subsp. *gallolyticus* (*SGG*) genomes from patients with colorectal cancer (CRC) and their comparison with isolates from non-CRC patients did not identify a specific subtype of strains associated with disease. This study has some limitations that need to be recognized, such as (i) the small number of *SGG* isolates associated with colorectal cancer; (ii) their limited geographical distribution; (iii) the comparison with only one non-human *SGG*.

Nevertheless, one key finding of this work is the hidden diversity among CRC-associated *SGG* isolates, which belong to various MLST types, exhibit different capsule compositions, and contain diverse T7SSb loci (Fig. 5). Furthermore, the TX20005 pathogenicity locus (SPAR), previously described as a potential cancer-associated determinant (53), is not conserved in *SGG* and was detected in only 3 out of 11 CRC-associated strains. However, the putative regulator of the type VII secretion system detected in the SPAR locus is present in all the SGG sequenced so far. Notably, the majority of human-derived *SGG* isolates (87%) carried at least one gene conferring resistance to an antibiotic, while the non-human strain, DSM16831, lacked any antibiotic resistance genes. This suggests that antibiotic resistance in human *SGG* isolates was likely acquired *in vivo* through horizontal gene transfer (HGT). These findings highlight the potential impact of host environment on the genetic adaptation of *SGG*. In sum, these genomic analyzes highlight the opportunistic nature of *SGG* and its complex association with cancer relying mostly on *SGG*’s ability to colonize the host colon and its ability to compete with bacterial members of the host colon microbiota through production of specific bacteriocin such as gallocin A and other LXG-effectors secreted by the specialized T7SSb whose roles need to be further studied.

## SUPPLEMENTAL MATERIAL

Figure S1 jb.00230-25-s0001.tif Genome characteristics of 40 SGG clinical isolates.

Figure S2 jb.00230-25-s0002.tif Sortase A-containing genetic loci.

Figure S3 jb.00230-25-s0003.tif Pilus loci in the various SGG isolates.

Figure S4 jb.00230-25-s0004.tif Hemicellulose loci in the various SGG isolates.

Figure S5 jb.00230-25-s0005.tif Glucan biosynthesis locus in the various SGG isolates.

Figure S6 jb.00230-25-s0006.tif Venn diagram comparing SGG to non-pathogenic SGM.

Figure S7 jb.00230-25-s0007.tif Schematic diagram of the Flg-new containing LPxTG proteins.

Supplemental figure legends jb.00230-25-s0008.docx Legends for Fig. S1 to S7.

Tables S1 to S5 jb.00230-25-s0009.pdf Genome similarity, antibiotic resistance genes, amino acid sequence of LPxTG proteins, putative lipoproteins, and putative virulence factors.

ASM does not own the copyrights to Supplemental Material that may be linked to, or accessed through, an article. The authors have granted ASM a non-exclusive, world-wide license to publish the Supplemental Material files. Please contact the corresponding author directly for reuse.

## References

[1] Khafipour E, Li S, Plaizier JC, Krause DO. 2009. Rumen microbiome composition determined using two nutritional models of subacute ruminal acidosis. Appl Environ Microbiol 75:7115–7124. doi:10.1128/AEM.00739-09 19783747 PMC2786511

[2] Périchon B, Lichtl-Häfele J, Bergsten E, Delage V, Trieu-Cuot P, Sansonetti P, Sobhani I, Dramsi S. 2022. Detection of Streptococcus gallolyticus and four other CRC-associated bacteria in patient stools reveals a potential “Driver” role for enterotoxigenic Bacteroides fragilis. Front Cell Infect Microbiol 12:794391. doi:10.3389/fcimb.2022.794391 35360109 PMC8963412

[3] Rusniok C, Couvé E, Da Cunha V, El Gana R, Zidane N, Bouchier C, Poyart C, Leclercq R, Trieu-Cuot P, Glaser P. 2010. Genome sequence of Streptococcus gallolyticus: insights into its adaptation to the bovine rumen and its ability to cause endocarditis. J Bacteriol 192:2266–2276. doi:10.1128/JB.01659-09 20139183 PMC2849448

[4] Papadimitriou K, Anastasiou R, Mavrogonatou E, Blom J, Papandreou NC, Hamodrakas SJ, Ferreira S, Renault P, Supply P, Pot B, Tsakalidou E. 2014. Comparative genomics of the dairy isolate Streptococcus macedonicus ACA-DC 198 against related members of the Streptococcus bovis/Streptococcus equinus complex. BMC Genomics 15:272. doi:10.1186/1471-2164-15-272 24713045 PMC4051162

[5] Danne C, Entenza JM, Mallet A, Briandet R, Débarbouillé M, Nato F, Glaser P, Jouvion G, Moreillon P, Trieu-Cuot P, Dramsi S. 2011. Molecular characterization of a Streptococcus gallolyticus genomic island encoding a pilus involved in endocarditis. J Infect Dis 204:1960–1970. doi:10.1093/infdis/jir666 22043018

[6] McCoy WC, Mason JM 3rd. 1951. Enterococcal endocarditis associated with carcinoma of the sigmoid; report of a case. J Med Assoc State Ala 21:162–166. 14880846

[7] Janney A, Powrie F, Mann EH. 2020. Host-microbiota maladaptation in colorectal cancer. Nature 585:509–517. doi:10.1038/s41586-020-2729-3 32968260

[8] White MT, Sears CL. 2024. The microbial landscape of colorectal cancer. Nat Rev Microbiol 22:240–254. doi:10.1038/s41579-023-00973-4 37794172

[9] Pleguezuelos-Manzano C, Puschhof J, Rosendahl Huber A, van Hoeck A, Wood HM, Nomburg J, Gurjao C, Manders F, Dalmasso G, Stege PB, et al.. 2020. Mutational signature in colorectal cancer caused by genotoxic pks+ E. coli. Nature 580:269–273. doi:10.1038/s41586-020-2080-8 32106218 PMC8142898

[10] Kumar R, Herold JL, Schady D, Davis J, Kopetz S, Martinez-Moczygemba M, Murray BE, Han F, Li Y, Callaway E, Chapkin RS, Dashwood W-M, Dashwood RH, Berry T, Mackenzie C, Xu Y. 2017. Streptococcus gallolyticus subsp. gallolyticus promotes colorectal tumor development. PLoS Pathog 13:e1006440. doi:10.1371/journal.ppat.1006440 28704539 PMC5509344

[11] Aymeric L, Donnadieu F, Mulet C, du Merle L, Nigro G, Saffarian A, Bérard M, Poyart C, Robine S, Regnault B, Trieu-Cuot P, Sansonetti PJ, Dramsi S. 2018. Colorectal cancer specific conditions promote Streptococcus gallolyticus gut colonization. Proc Natl Acad Sci USA 115:E283–E291. doi:10.1073/pnas.1715112115 29279402 PMC5777054

[12] Pasquereau-Kotula E, du Merle L, Sismeiro O, Pietrosemoli N, Varet H, Legendre R, Trieu-Cuot P, Dramsi S. 2023. Transcriptome profiling of human col\onic cells exposed to the gut pathobiont Streptococcus gallolyticus subsp. gallolyticus. PLoS ONE 18:e0294868. doi:10.1371/journal.pone.0294868 38033043 PMC10688619

[13] Knippel RJ, Sears CL. 2021. The microbiome colorectal cancer puzzle: initiator, propagator, and avenue for treatment and research. J Natl Compr Canc Netw 19:986–992. doi:10.6004/jnccn.2021.7062 34416704

[14] Dejea CM, Fathi P, Craig JM, Boleij A, Taddese R, Geis AL, Wu X, DeStefano Shields CE, Hechenbleikner EM, Huso DL, Anders RA, Giardiello FM, Wick EC, Wang H, Wu S, Pardoll DM, Housseau F, Sears CL. 2018. Patients with familial adenomatous polyposis harbor colonic biofilms containing tumorigenic bacteria. Science 359:592–597. doi:10.1126/science.aah3648 29420293 PMC5881113

[15] Homburg S, Oswald E, Hacker J, Dobrindt U. 2007. Expression analysis of the colibactin gene cluster coding for a novel polyketide in Escherichia coli. FEMS Microbiol Lett 275:255–262. doi:10.1111/j.1574-6968.2007.00889.x 17714479

[16] Moncrief JS, Duncan AJ, Wright RL, Barroso LA, Wilkins TD. 1998. Molecular characterization of the fragilysin pathogenicity islet of enterotoxigenic Bacteroides fragilis. Infect Immun 66:1735–1739. doi:10.1128/IAI.66.4.1735-1739.1998 9529104 PMC108111

[17] Valguarnera E, Wardenburg JB. 2020. Good gone bad: one toxin away from disease for Bacteroides fragilis. J Mol Biol 432:765–785. doi:10.1016/j.jmb.2019.12.003 31857085

[18] Zepeda-Rivera M, Minot SS, Bouzek H, Wu H, Blanco-Míguez A, Manghi P, Jones DS, LaCourse KD, Wu Y, McMahon EF, Park S-N, Lim YK, Kempchinsky AG, Willis AD, Cotton SL, Yost SC, Sicinska E, Kook J-K, Dewhirst FE, Segata N, Bullman S, Johnston CD. 2024. A distinct Fusobacterium nucleatum clade dominates the colorectal cancer niche. Nature 628:424–432. doi:10.1038/s41586-024-07182-w 38509359 PMC11006615

[19] Bergsten E, Mestivier D, Donnadieu F, Pedron T, Barau C, Meda LT, Mettouchi A, Lemichez E, Gorgette O, Chamaillard M, Vaysse A, Volant S, Doukani A, Sansonetti PJ, Sobhani I, Nigro G. 2023. Parvimonas micra, an oral pathobiont associated with colorectal cancer, epigenetically reprograms human colonocytes. Gut Microbes 15:2265138. doi:10.1080/19490976.2023.2265138 37842920 PMC10580862

[20] Cokelaer T, Desvillechabrol D, Legendre R, Cardon M. 2017. “Sequana”: a set of snakemake NGS pipelines. JOSS 2:352. doi:10.21105/joss.00352

[21] Koren S, Walenz BP, Berlin K, Miller JR, Bergman NH, Phillippy AM. 2017. Canu: scalable and accurate long-read assembly via adaptive k-mer weighting and repeat separation. Genome Res 27:722–736. doi:10.1101/gr.215087.116 28298431 PMC5411767

[22] Hunt M, Silva ND, Otto TD, Parkhill J, Keane JA, Harris SR. 2015. Circlator: automated circularization of genome assemblies using long sequencing reads. Genome Biol 16:294. doi:10.1186/s13059-015-0849-0 26714481 PMC4699355

[23] Aziz RK, Bartels D, Best AA, DeJongh M, Disz T, Edwards RA, Formsma K, Gerdes S, Glass EM, Kubal M, et al.. 2008. The RAST server: rapid annotations using subsystems technology. BMC Genomics 9:75. doi:10.1186/1471-2164-9-75 18261238 PMC2265698

[24] Teh WK, Ding Y, Gubellini F, Filloux A, Poyart C, Givskov M, Dramsi S. 2023. Characterization of TelE, a T7SS LXG effector exhibiting a conserved C-terminal glycine zipper motif required for toxicity. Microbiol Spectr 11:e0148123. doi:10.1128/spectrum.01481-23 37432124 PMC10434224

[25] Taylor JC, Gao X, Xu J, Holder M, Petrosino J, Kumar R, Liu W, Höök M, Mackenzie C, Hillhouse A, Brashear W, Nunez MP, Xu Y. 2021. A type VII secretion system of Streptococcus gallolyticus subsp. gallolyticus contributes to gut colonization and the development of colon tumors. PLoS Pathog 17:e1009182. doi:10.1371/journal.ppat.1009182 33406160 PMC7815207

[26] Knight RG, Shlaes DM. 1985. Physiological characteristics and deoxyribonucleic acid relatedness of human isolates of Streptococcus bovis and Streptococcus bovis (var.). Int J Syst Bacteriol 35:357–361. doi:10.1099/00207713-35-3-357

[27] Hinse D, Vollmer T, Rückert C, Blom J, Kalinowski J, Knabbe C, Dreier J. 2011. Complete genome and comparative analysis of Streptococcus gallolyticus subsp. gallolyticus, an emerging pathogen of infective endocarditis. BMC Genomics 12:400. doi:10.1186/1471-2164-12-400 21824414 PMC3173452

[28] Grimm I, Dumke J, Vollmer T, Hinse D, Rückert C, Kalinowski J, Knabbe C, Dreier J. 2017. Complete genome sequence of the Streptococcus gallolyticus subsp. gallolyticus strain DSM 16831. Genome Announc 5:e00108–17. doi:10.1128/genomeA.00108-17 28428288 PMC5399247

[29] Papadimitriou K, Ferreira S, Papandreou NC, Mavrogonatou E, Supply P, Pot B, Tsakalidou E. 2012. Complete genome sequence of the dairy isolate Streptococcus macedonicus ACA-DC 198. J Bacteriol 194:1838–1839. doi:10.1128/JB.06804-11 22408241 PMC3302469

[30] Papadimitriou K, Mavrogonatou E, Bolotin A, Tsakalidou E, Renault P. 2016. Whole-genome sequence of the cheese isolate Streptococcus macedonicus 679. Genome Announc 4:e01025-16. doi:10.1128/genomeA.01025-16 27660795 PMC5034146

[31] Perrin A, Rocha EPC. 2020. PanACoTA: a modular tool for massive microbial comparative genomics. Bioinformatics. doi:10.1101/2020.09.11.293472 PMC7803007 33575648

[32] Richter M, Rosselló-Móra R, Oliver Glöckner F, Peplies J. 2016. JSpeciesWS: a web server for prokaryotic species circumscription based on pairwise genome comparison. Bioinformatics 32:929–931. doi:10.1093/bioinformatics/btv681 26576653 PMC5939971

[33] Ozer EA, Allen JP, Hauser AR. 2014. Characterization of the core and accessory genomes of Pseudomonas aeruginosa using bioinformatic tools Spine and AGEnt. BMC Genomics 15:737. doi:10.1186/1471-2164-15-737 25168460 PMC4155085

[34] Dumke J, Hinse D, Vollmer T, Knabbe C, Dreier J. 2014. Development and application of a multilocus sequence typing scheme for Streptococcus gallolyticus subsp. gallolyticus. J Clin Microbiol 52:2472–2478. doi:10.1128/JCM.03329-13 24789199 PMC4097760

[35] Jolley KA, Bray JE, Maiden MCJ. 2018. Open-access bacterial population genomics: BIGSdb software, the PubMLST.org website and their applications. Wellcome Open Res 3:124. doi:10.12688/wellcomeopenres.14826.1 30345391 PMC6192448

[36] Teufel F, Almagro Armenteros JJ, Johansen AR, Gíslason MH, Pihl SI, Tsirigos KD, Winther O, Brunak S, von Heijne G, Nielsen H. 2022. SignalP 6.0 predicts all five types of signal peptides using protein language models. Nat Biotechnol 40:1023–1025. doi:10.1038/s41587-021-01156-3 34980915 PMC9287161

[37] Yu NY, Wagner JR, Laird MR, Melli G, Rey S, Lo R, Dao P, Sahinalp SC, Ester M, Foster LJ, Brinkman FSL. 2010. PSORTb 3.0: improved protein subcellular localization prediction with refined localization subcategories and predictive capabilities for all prokaryotes. Bioinformatics 26:1608–1615. doi:10.1093/bioinformatics/btq249 20472543 PMC2887053

[38] Liu B, Zheng D, Zhou S, Chen L, Yang J. 2022. VFDB 2022: a general classification scheme for bacterial virulence factors. Nucleic Acids Res 50:D912–D917. doi:10.1093/nar/gkab1107 34850947 PMC8728188

[39] McArthur AG, Waglechner N, Nizam F, Yan A, Azad MA, Baylay AJ, Bhullar K, Canova MJ, De Pascale G, Ejim L, et al.. 2013. The comprehensive antibiotic resistance database. Antimicrob Agents Chemother 57:3348–3357. doi:10.1128/AAC.00419-13 23650175 PMC3697360

[40] Bertelli C, Laird MR, Williams KP, Simon Fraser University Research Computing Group, Lau BY, Hoad G, Winsor GL, Brinkman FSL. 2017. IslandViewer 4: expanded prediction of genomic islands for larger-scale datasets. Nucleic Acids Res 45:W30–W35. doi:10.1093/nar/gkx343 28472413 PMC5570257

[41] Sillanpää J, Nallapareddy SR, Qin X, Singh KV, Muzny DM, Kovar CL, Nazareth LV, Gibbs RA, Ferraro MJ, Steckelberg JM, Weinstock GM, Murray BE. 2009. A collagen-binding adhesin, Acb, and ten other putative MSCRAMM and pilus family proteins of Streptococcus gallolyticus subsp. gallolyticus (Streptococcus bovis Group, biotype I). J Bacteriol 191:6643–6653. doi:10.1128/JB.00909-09 19717590 PMC2795296

[42] Corredoira J, García-País MJ, Coira A, Rabuñal R, García-Garrote F, Pita J, Rodríguez-Macías A, Blanco M, Lopez-Roses L, López-Álvarez MJ, Alonso-García MP. 2015. Differences between endocarditis caused by Streptococcus bovis and Enterococcus spp. and their association with colorectal cancer. Eur J Clin Microbiol Infect Dis 34:1657–1665. doi:10.1007/s10096-015-2402-1 26017665

[43] Da Cunha V, Davies MR, Douarre P-E, Rosinski-Chupin I, Margarit I, Spinali S, Perkins T, Lechat P, Dmytruk N, Sauvage E, et al.. 2014. Streptococcus agalactiae clones infecting humans were selected and fixed through the extensive use of tetracycline. Nat Commun 5:4544. doi:10.1038/ncomms5544 25088811 PMC4538795

[44] Derbise A, de Cespedes G, el Solh N. 1997. Nucleotide sequence of the Staphylococcus aureus transposon, Tn5405, carrying aminoglycosides resistance genes. J Basic Microbiol 37:379–384. doi:10.1002/jobm.3620370511 9373952

[45] Fischetti VA. 2019. Surface proteins on Gram-positive bacteria. Microbiol Spectr 7:7. doi:10.1128/microbiolspec.gpp3-0012-2018 PMC6684298 31373270

[46] Marraffini LA, Dedent AC, Schneewind O. 2006. Sortases and the art of anchoring proteins to the envelopes of gram-positive bacteria. Microbiol Mol Biol Rev 70:192–221. doi:10.1128/MMBR.70.1.192-221.2006 16524923 PMC1393253

[47] Mazmanian SK, Ton-That H, Schneewind O. 2001. Sortase-catalysed anchoring of surface proteins to the cell wall of Staphylococcus aureus. Mol Microbiol 40:1049–1057. doi:10.1046/j.1365-2958.2001.02411.x 11401711

[48] Dramsi S, Trieu-Cuot P, Bierne H. 2005. Sorting sortases: a nomenclature proposal for the various sortases of Gram-positive bacteria. Res Microbiol 156:289–297. doi:10.1016/j.resmic.2004.10.011 15808931

[49] Danne C, Dramsi S. 2012. Pili of gram-positive bacteria: roles in host colonization. Res Microbiol 163:645–658. doi:10.1016/j.resmic.2012.10.012 23116627

[50] Martins M, Aymeric L, du Merle L, Danne C, Robbe-Masselot C, Trieu-Cuot P, Sansonetti P, Dramsi S. 2015. Streptococcus gallolyticus Pil3 Pilus is required for adhesion to colonic mucus and for colonization of mouse distal colon. J Infect Dis 212:1646–1655. doi:10.1093/infdis/jiv307 26014801

[51] Isenring J, Köhler J, Nakata M, Frank M, Jans C, Renault P, Danne C, Dramsi S, Kreikemeyer B, Oehmcke-Hecht S. 2018. Streptococcus gallolyticus subsp. gallolyticus endocarditis isolate interferes with coagulation and activates the contact system. Virulence 9:248–261. doi:10.1080/21505594.2017.1393600 29072555 PMC5955193

[52] Lin I-H, Liu T-T, Teng Y-T, Wu H-L, Liu Y-M, Wu K-M, Chang C-H, Hsu M-T. 2011. Sequencing and comparative genome analysis of two pathogenic Streptococcus gallolyticus subspecies: genome plasticity, adaptation and virulence. PLoS One 6:e20519. doi:10.1371/journal.pone.0020519 21633709 PMC3102119

[53] Taylor JC, Kumar R, Xu J, Xu Y. 2023. A pathogenicity locus of Streptococcus gallolyticus subspecies gallolyticus. Sci Rep 13:6291. doi:10.1038/s41598-023-33178-z 37072463 PMC10113328

[54] Maragkoudakis PA, Papadelli M, Georgalaki M, Panayotopoulou EG, Martinez-Gonzalez B, Mentis AF, Petraki K, Sgouras DN, Tsakalidou E. 2009. In vitro and in vivo safety evaluation of the bacteriocin producer Streptococcus macedonicus ACA-DC 198. Int J Food Microbiol 133:141–147. doi:10.1016/j.ijfoodmicro.2009.05.012 19515446

[55] Proutière A, du Merle L, Garcia-Lopez M, Léger C, Voegele A, Chenal A, Harrington A, Tal-Gan Y, Cokelaer T, Trieu-Cuot P, Dramsi S. 2023. Gallocin A, an atypical two-peptide bacteriocin with intramolecular disulfide bonds required for activity. Microbiol Spectr 11:e0508522. doi:10.1128/spectrum.05085-22 36951576 PMC10100652

[56] Georgalaki MD, Van Den Berghe E, Kritikos D, Devreese B, Van Beeumen J, Kalantzopoulos G, De Vuyst L, Tsakalidou E. 2002. Macedocin, a food-grade lantibiotic produced by Streptococcus macedonicus ACA-DC 198. Appl Environ Microbiol 68:5891–5903. doi:10.1128/AEM.68.12.5891-5903.2002 12450808 PMC134371

[57] Georgalaki M, Papadimitriou K, Anastasiou R, Pot B, Van Driessche G, Devreese B, Tsakalidou E. 2013. Macedovicin, the second food-grade lantibiotic produced by Streptococcus macedonicus ACA-DC 198. Food Microbiol 33:124–130. doi:10.1016/j.fm.2012.09.008 23122510

