# Supplementary figures and images for: Colorectal cancer-associated Streptococcus gallolyticus: a hidden diversity expose
Source: J Bacteriol. 2025 Aug 14;207(9):e00230-25. doi: 10.1128/jb.00230-25 (PMC12445087; doi:10.1128/jb.00230-25)

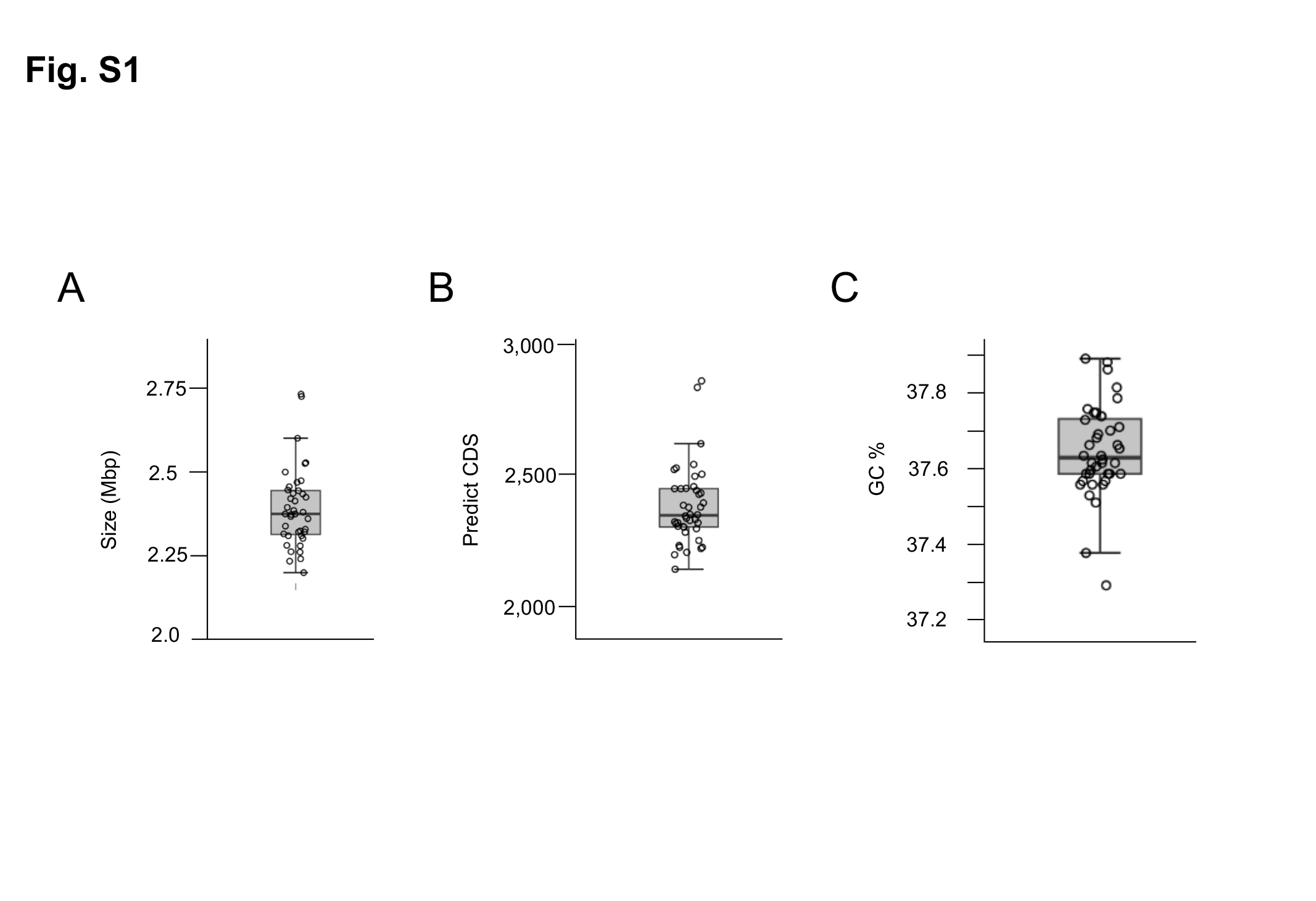

Supplement: Figure S1 — Genome characteristics of 40 SGG clinical isolates. [file jb.00230-25-s0001.tif]

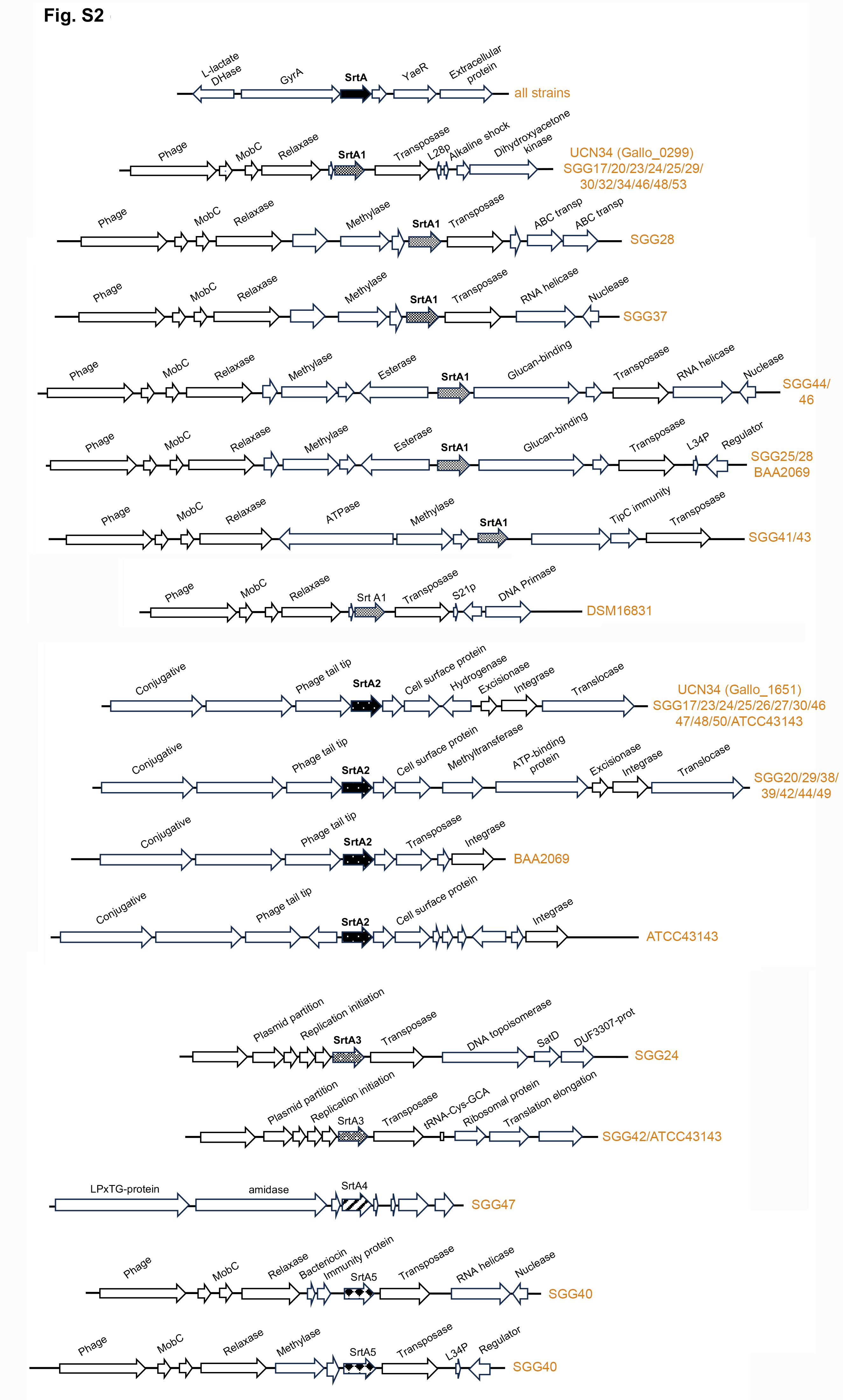

Supplement: Figure S2 — Sortase A-containing genetic loci. [file jb.00230-25-s0002.tif]

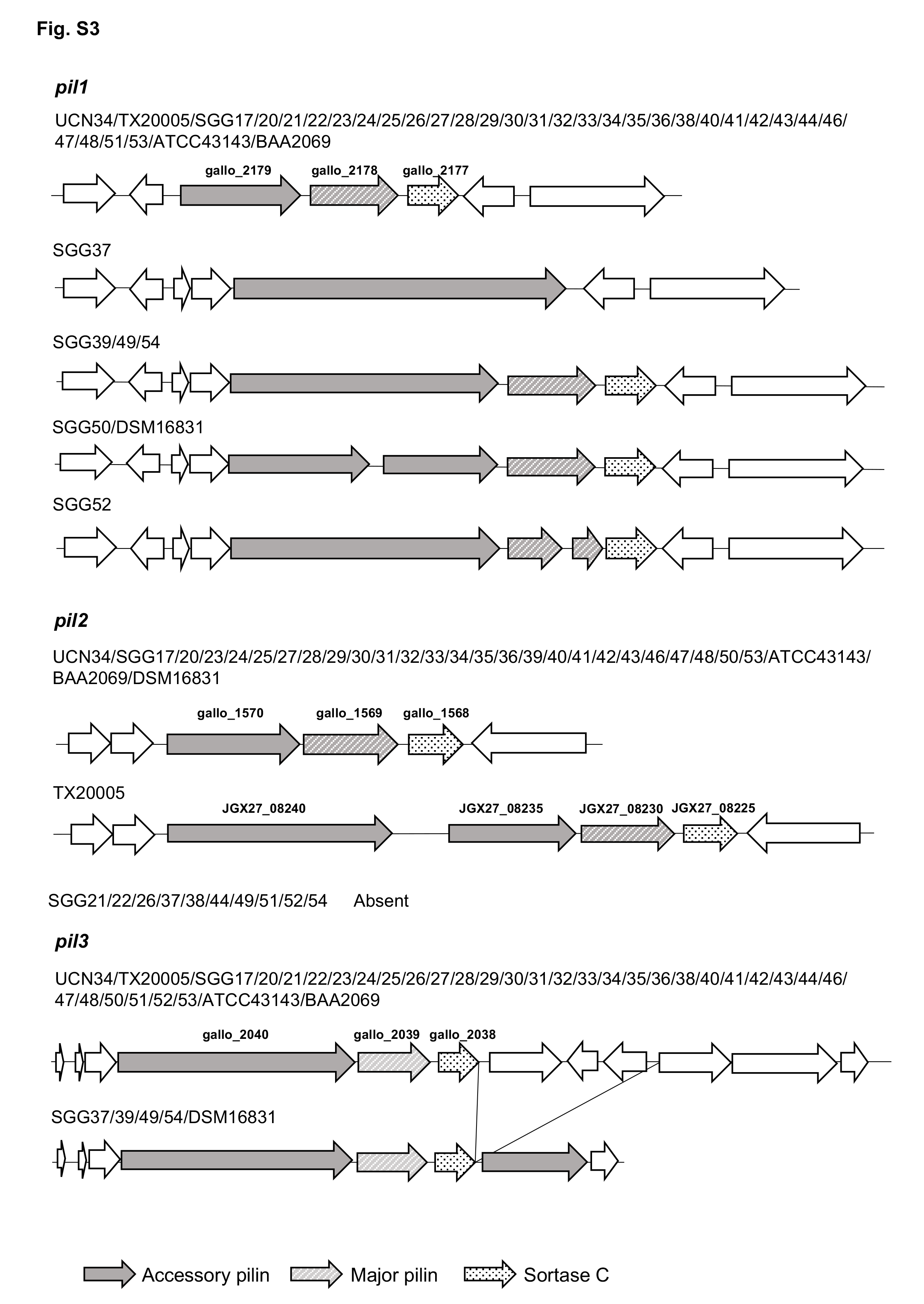

Supplement: Figure S3 — Pilus loci in the various SGG isolates. [file jb.00230-25-s0003.tif]

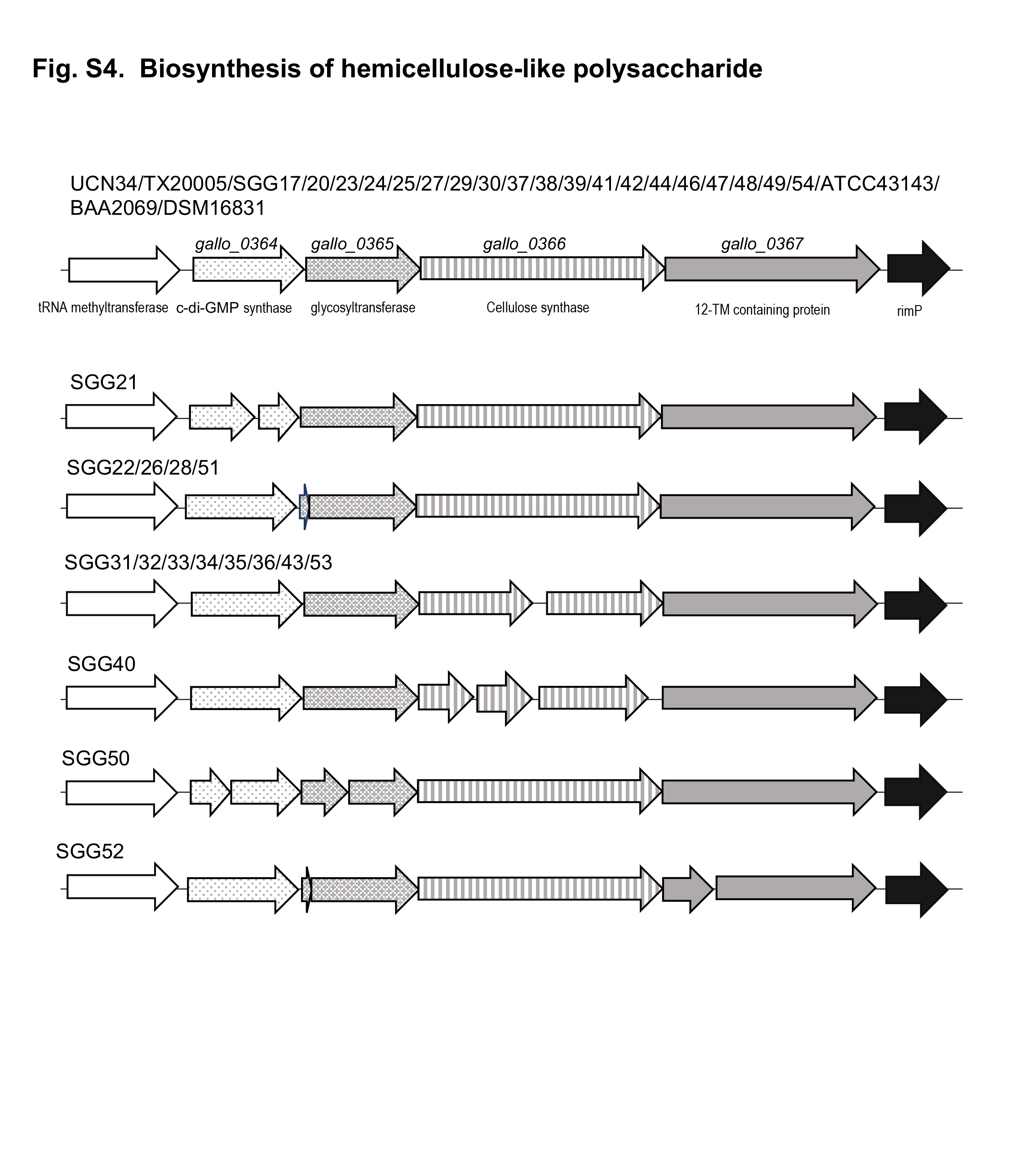

Supplement: Figure S4 — Hemicellulose loci in the various SGG isolates. [file jb.00230-25-s0004.tif]

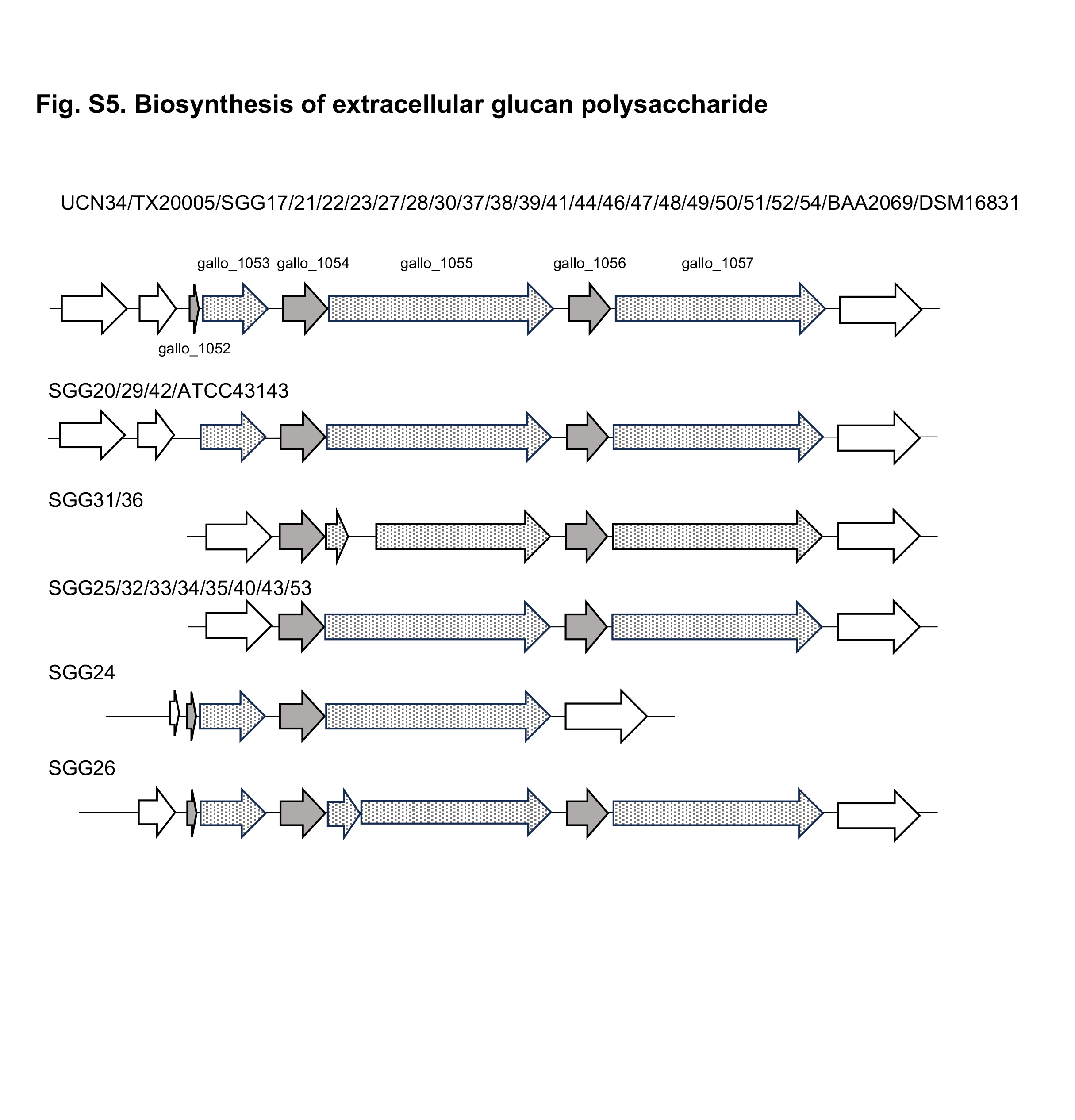

Supplement: Figure S5 — Glucan biosynthesis locus in the various SGG isolates. [file jb.00230-25-s0005.tif]

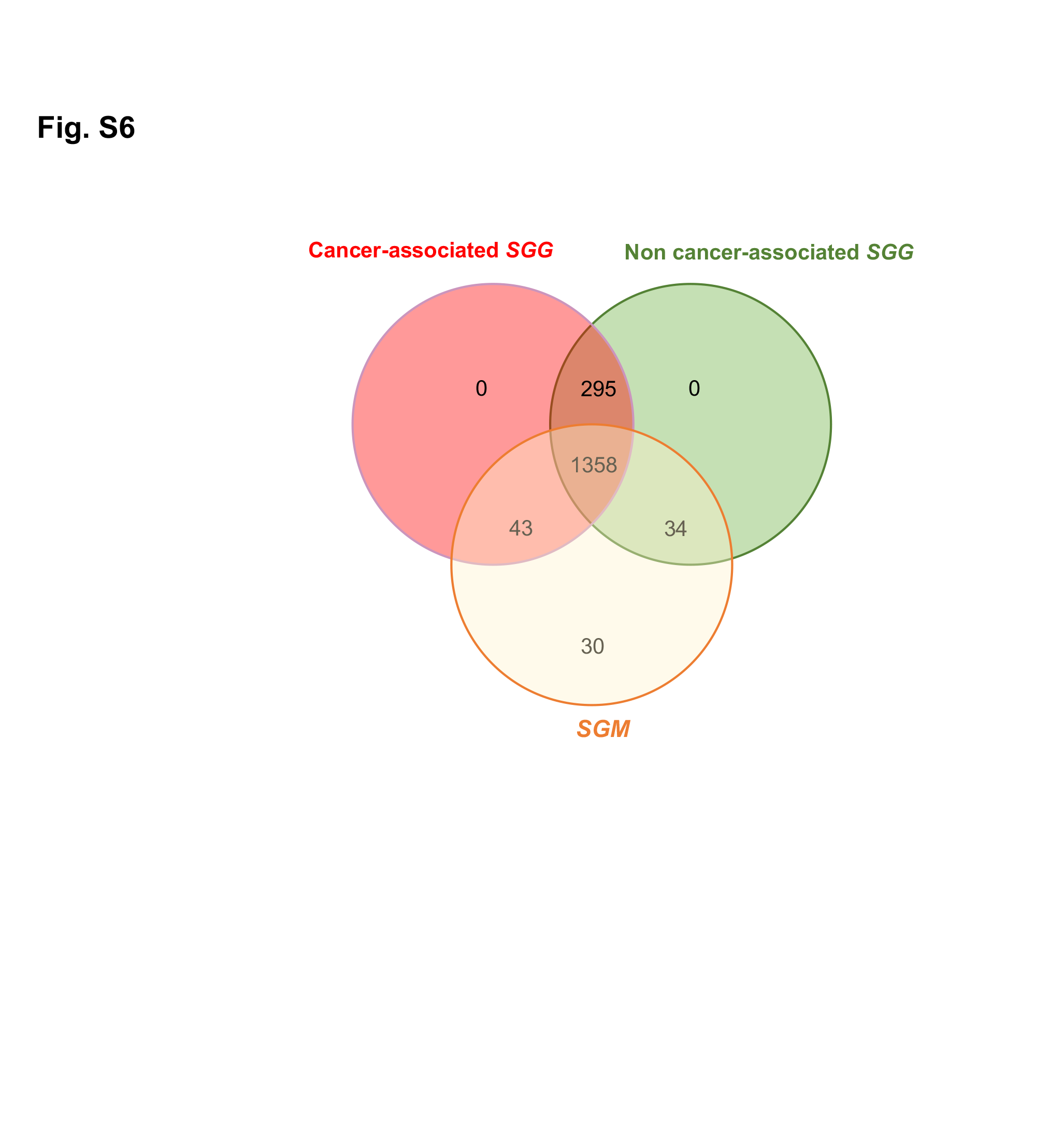

Supplement: Figure S6 — Venn diagram comparing SGG to non-pathogenic SGM. [file jb.00230-25-s0006.tif]

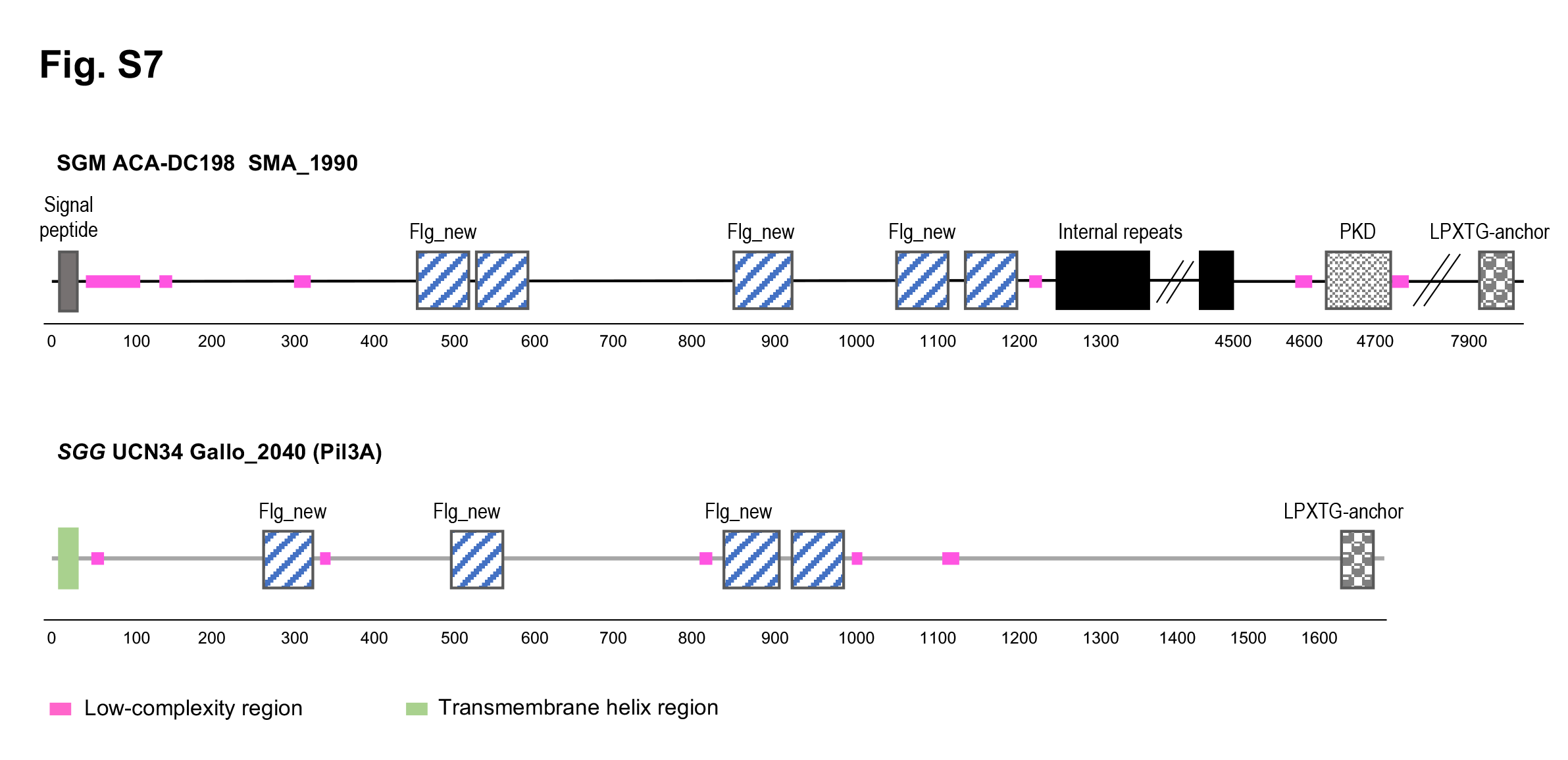

Supplement: Figure S7 — Schematic diagram of the Flg-new containing LPxTG proteins. [file jb.00230-25-s0007.tif]
