# Supplementary material for: Colorectal cancer-associated Streptococcus gallolyticus: a hidden diversity expose
Source: J Bacteriol. 2025 Aug 14;207(9):e00230-25. doi: 10.1128/jb.00230-25 (PMC12445087; doi:10.1128/jb.00230-25)
Supplement: Supplemental figure legends — Legends for Fig. S1 to S7. [file jb.00230-25-s0008.docx]

**Supplementary figures legend**

**Fig. S1**. Whole genome characteristics of 40 *S. gallolyticus* subsp. *gallolyticus* strains.

**Fig. S2**. Schematic representation of the genetic localisation of the various *srtA* genes.

**Fig. S3**. Schematic representation of the three pilus loci involved in pili biosynthesis. Arrows indicate sense of transcription. Nomenclature indicated in the upper locus is accordingly to UCN34 (3).

**Fig. S4**. Hemicellulose biosynthesis. Schematic representation of the locus involved in hemicellulose biosynthesis in the 40 *S. gallolyticus* strains. Arrows indicate sense of transcription. Nomenclature indicated in the upper locus is accordingly to UCN34 (3).

**Fig. S5**. Glucan biosynthesis. Schematic representation of the locus involved in glucan biosynthesis in the 40 *S. gallolyticus* strains. Grey and dashed grey arrows indicate genes encoding glycosyltransferases and regulators, respectively. Nomenclature indicated in the upper locus is accordingly to UCN34 (3).

**Fig. S6**. Venn diagram showing the distribution of shared orthologous clusters among core genomes of CRC-, non CRC-associated strains, and *SGM*. The numbers of unique and shared orthologous clusters of each core genome is indicated.

**Fig. S7**. Schematic representation of predicted domains within SMA_1190 of SGM_ACA-DC198 (upper) and Gallo_2040 protein of UCN34 (lower) using the Simple Modular Architecture Research Tool web server (SMART, <http://smart.embl-heidelberg.de/>). Flg-New is a novel
