# Supplementary material for: Colorectal cancer-associated Streptococcus gallolyticus: a hidden diversity expose
Source: J Bacteriol. 2025 Aug 14;207(9):e00230-25. doi: 10.1128/jb.00230-25 (PMC12445087; doi:10.1128/jb.00230-25)
Supplement: Tables S1 to S5 — Genome similarity, antibiotic resistance genes, amino acid sequence of LPxTG proteins, putative lipoproteins, and putative virulence factors. [file jb.00230-25-s0009.pdf]

**Table S1. Genome similarity between cancer-, non cancer-associated, and non human *SGG* isolated strains based on average nucleotide identity (ANI)**

| | | Cancer group | | | | | | | | | | | Non cancer group | | | | | | | | | Non human |
|---|---|---|---|---|---|---|---|---|---|---|---|---|---|---|---|---|---|---|---|---|---|---|
| | | UCN34 | TX20005 | SGG30 | SGG37 | SGG40 | SGG42 | SGG44 | SGG20 | SGG33 | SGG34 | SGG41 | SGG23 | SGG24 | SGG25 | SGG26 | SGG27 | SGG47 | SGG49 | SGG50 | SGG52 | DSM16831 |
| **cancer group** | UCN34 | | 99.32 | 99.99 | 99.04 | 99.38 | 99.49 | 99.26 | 99.80 | 99.56 | 99.60 | 99.57 | 99.92 | 99.47 | 99.80 | 99.13 | 99.49 | 99.48 | 99.01 | 99.35 | 99.03 | 98.96 |
| | TX20005 | | | 99.32 | 99.12 | 99.16 | 99.39 | 99.35 | 99.32 | 99.30 | 99.36 | 99.40 | 99.33 | 99.42 | 99.28 | 99.26 | 99.47 | 99.46 | 99.13 | 99.05 | 99.19 | 99.17 |
| | SGG30 | | | | 99.04 | 99.38 | 99.49 | 99.26 | 99.80 | 99.56 | 99.60 | 99.57 | 99.92 | 99.47 | 99.80 | 99.13 | 99.50 | 99.49 | 99.01 | 99.35 | 99.03 | 98.96 |
| | SGG37 | | | | | 98.97 | 99.12 | 99.00 | 99.10 | 99.12 | 99.10 | 99.15 | 99.04 | 99.00 | 98.97 | 99.05 | 98.99 | 98.96 | 99.92 | 98.98 | 99.04 | 99.12 |
| | SGG40 | | | | | | 99.34 | 99.08 | 99.42 | 99.77 | 99.72 | 99.57 | 99.41 | 98.96 | 99.51 | 98.94 | 99.14 | 98.97 | 98.99 | 99.14 | 98.95 | 98.88 |
| | SGG42 | | | | | | | 99.19 | 99.58 | 99.61 | 99.67 | 99.78 | 99.50 | 99.22 | 99.42 | 99.30 | 99.30 | 99.16 | 99.07 | 99.13 | 99.22 | 99.13 |
| | SGG44 | | | | | | | | 99.36 | 99.16 | 99.20 | 99.25 | 99.28 | 99.24 | 99.21 | 99.08 | 99.35 | 99.36 | 99.03 | 99.07 | 99.03 | 99.01 |
| | SGG20 | | | | | | | | | 99.57 | 99.60 | 99.60 | 99.82 | 99.54 | 99.80 | 99.19 | 99.55 | 99.55 | 99.11 | 99.41 | 99.06 | 99.02 |
| | SGG33 | | | | | | | | | | 99.93 | 99.72 | 99.59 | 99.31 | 99.71 | 99.23 | 99.30 | 99.21 | 99.10 | 99.29 | 99.11 | 99.00 |
| | SGG34 | | | | | | | | | | | 99.69 | 99.60 | 99.22 | 99.73 | 99.23 | 99.32 | 99.29 | 99.12 | 99.30 | 99.17 | 98.95 |
| | SGG41 | | | | | | | | | | | | 99.57 | 99.35 | 99.55 | 99.33 | 99.37 | 99.36 | 99.15 | 99.26 | 99.15 | 99.08 |
| **No cancer group** | SGG23 | | | | | | | | | | | | | 99.49 | 99.87 | 99.12 | 99.48 | 99.47 | 99.03 | 99.38 | 99.05 | 98.96 |
| | SGG24 | | | | | | | | | | | | | | 99.45 | 99.08 | 99.93 | 99.82 | 99.01 | 99.17 | 99.06 | 98.92 |
| | SGG25 | | | | | | | | | | | | | | | 99.05 | 99.45 | 99.43 | 98.99 | 99.38 | 99.01 | 98.91 |
| | SGG26 | | | | | | | | | | | | | | | | 99.17 | 99.00 | 99.02 | 98.97 | 99.47 | 98.98 |
| | SGG27 | | | | | | | | | | | | | | | | | 99.99 | 98.98 | 99.14 | 99.14 | 99.01 |
| | SGG47 | | | | | | | | | | | | | | | | | | 98.93 | 99.14 | 99.06 | 98.93 |
| | SGG49 | | | | | | | | | | | | | | | | | | | 98.96 | 99.07 | 99.17 |
| | SGG50 | | | | | | | | | | | | | | | | | | | | 98.96 | 99.00 |
| | SGG52 | | | | | | | | | | | | | | | | | | | | | 98.95 |
| **Non human DSM16831** | DSM16831 | | | | | | | | | | | | | | | | | | | | | |

| |
|---|
| >99.9 |
| 99.8-99.9 |
| 99.6-99.8 |
| 99.5-99.6 |
| 99.4-99.5 |
| 99.3-99.4 |
| 99.2-99.3 |
| 99.1-99.2 |
| 99-99.1 |
| ≤99 |

ANI based on MUMmer were determined by using the Jspecies web server (*23)*

**Table S2. List of antibiotic resistance genes found in the genomes of *SGG* isolates**

| | | Antibiotic resistance gene | | | | | | |
|---|---|---|---|---|---|---|---|---|
| | | *ermB* | *aphA-3* | *sat4* | *aadE* | *tet(M)* or *tet(L)* or *tet(O)* | *dfrF* | *catA9* |
| **CRC-associated strains** | UCN34 | | | | | + | | |
| | TX20005 | | | | | + | | |
| | SGG20 | + | + | + | + | + | | |
| | SGG30 | | | | | + | | |
| | SGG37 | | | | | | | |
| | SGG40 | + | + | + | + | + | | |
| | SGG42 | | | | | | | |
| | SGG44 | + | + | + | + | | | |
| | SGG33 | + | | | + | + | | + |
| | SGG34 | + | | | | + | | |
| | SGG41 | | | | | | | |
| **Non cancer-associated strains** | SGG23 | + | + | + | + | + | | |
| | SGG24 | + | + | + | + | + | | |
| | SGG25 | + | + | + | + | + | | |
| | SGG26 | | | | | + | | |
| | SGG27 | + | + | + | + | + | | |
| | SGG47 | + | + | + | + | + | | |
| | SGG49 | | | | | | | |
| | SGG50 | + | + | + | + | + | | |
| | SGG52 | + | | | | + | | + |
| **Non human-isolate** | DSM16831 | | | | | | | |
| **Other strains clinically undefined** | SGG17 | + | + | + | + | + | | |
| | SGG21 | | | | | + | | |
| | SGG22 | + | + | + | + | + | | |
| | SGG28 | + | + | + | + | + | + | |
| | SGG29 | + | + | + | + | + | | |
| | SGG31 | + | | | | + | | |
| | SGG32 | + | | | | + | | |
| | SGG35 | | | | | + | | |
| | SGG36 | | | | | + | | |
| | SGG38 | | | | | + | | |
| | SGG39 | | | | | + | | |
| | SGG43 | + | + | + | + | + | | |
| | SGG46 | + | + | + | + | + | | |
| | SGG48 | + | + | + | + | + | | |
| | SGG51 | + | + | + | + | + | | |
| | SGG53 | | | | | + | | |
| | SGG54 | | | | | + | | |
| | ATCC43143 | | | | | + | | |
| | BAA2069 | | | | | | | |

**Table S3. Amino acid sequence of the putative cell-wall anchored proteins (LPxTG) found in the pangenome of 40 SGG isolates**

| LPxTG | Sequence |
|---|---|
| 1 | MKKEQEKKCVNWFMHKRGKQWIYGCGVLVCGIVLGTVATPVMADEAVSSPMELVTVADVNADNNTNTDQKTEVDQETQQPANQVETPENQTPIEQGVVGEQNQKVTEENQVTENQDVTQQNQVTENQEPATKTQDDAQKTETTDAEEKVEVTDSLKQKADQPNESTEKARKALSTNLTTKKESSYNTNLQGLSYDANVWEVREDGLYSNAIGEGDSFLLSTSAGKNFVFQTDVTFLQNTGAASLVFRSTGDAQNLKGYVVNLDGNSHKIKFMRWGEANLIDEKEIEATSDNKYSLKVVAANGWISYYINGILVANLSDYTIQRDDRGQTTYIKDGNFSLLNWNGEMIFQNTFYRELTDAELPILKDVTVSSKNGPVEPKGQFFPEGAVYIQYVSHDASTVDLSFVPNNQDAVIKVTDDQGNVYSDPSNIPVSVGANYLTVTSTYTVDGYEVTSTYRINVHRRQSAEVYYNENFRDQYHYSVKDGWANDPNGLVYYNGVYHMFYQFYDDIQWGPMHWAHATSTDLIHWEDQPIAFYPDYNGAMFSGCIVADPNNTSGLFEGDKGGLVALITADGEGQRIKVAYSKDEGKTWQKLDEVAADWSTDPLQNRDFRDPKVFRWEGKWFMVLAGGPLRIYSSDNLLDWSVESTYPDLHTECPDLYPIMAEGNTVKWVLSRGGRYYKVGDLKQVDGHWKFVADADYQESDGIMNFGKDSYAAMTYYVQDFGTKDNPTIPQIIELNWMNTWDNYCNLVAERTGQKFNGTFNLNLTLGLVKDGDKYVLTQTPIKAYESLRDVDHKVEYKDVVVGKDNNLFKDFSGDTYEIVAHFKPSDRTTKVGFNLRVGQGEVTKVYYDLQTGRIAIDRSQSGIILTELFRNVDSQAVTRNADGSIDLHIFVDRASVEVFTKGGTVTGANQIFTSPQSLGLGVFAEGYEAKADIALYPLKSIWKDKVETTKPQSIVPASAKNVRMNVGDSTVVKAYVSPAVVNQDLLWSILNNGNVSTEISGNQVFVKALKKGQVIVRAQSKTDPSVYQDFVLDILEDNFKTNVKNVKVFAGDWHADGESLKVENHNSNDIYMAADKMPYENYQMDLDIKYGRGVVNIFFASGNPDANNAYSIQFGGDNSVRLFRFYSDTISESQMTAAINDNQFHHVRLVKSANAIQVFVDNQLAMSYTFDQVEDFFNNPYIGLGLWDGELEVQNFFVVDLDAKEPTQNEEKVEVVPTDPQTPAEQVVTTTTLAAKAPAKSEKATDAKAPVIPKTALVSETVLPQTGEKDSHLAGLGIVSILAAMGAFFGQFFKKKES |
| 2 | MTKKTILAALTLSTVALTQAGLASADEVSSPETPNEVLVVTETQPIDPSTPIVPDVPTDPSTPPTTPDVPTDPETPPTSPDTGGGDTGGETTPDQPTDPSTPPSTDGSDTDTSGGDNNPGTGGDTEAPSNPETGEDSNTPSQPTTPPITDDAVNKDDQGNMTVKPQVVAPNQTVVGTQNGQLLVQDASGNTRLAEASEFGGQKNDNGTVSFKTADGKMVTLPETGSTNGTLLSLLGSGLVGLLGFFWKKKRAM |
| 3 | MNTGVKDVMKKAKVTVAATALLAGATLAQTAHADEVDSSATLAEEQQNQSVTTSVTQTDVDNAKGELDKANNAVDNQNQAVNNAQQNANNAQDAYDQAQQDTNNAQDLVDKATPEEIENATNGVTDSQNKVDQAEKDQEQAEQNANNAQGAVNNAQNKVDQAQDQVNDAQNQVNDAQSKVDQAQDILDGTGSKDILDEAEAAKKDEATKKDDLESAKDQLEEAEKADKDRQNAIDKAQSELDKTEQELADKKTDLDQAQSDVNDKTIAQNQAQTNVDDAQKVVDNLLDEVANHNIITIPSGYGDKLKDFYLNGTAENSQALKEAAKVGLTNNAYKSNNVDRVVINDVNHLTEAQRRELTQFAVDLLNQVRSALGAELVVTNSSAMAFADEVANTSKELGHDTTAIPTAAAKFGLYSVQGVNHYENFSSGYFNPEKVLTMNDLKRALYNTILEMLFDDDTSSWGHTISLTGVGGFYGQSASKYIGIDFSILNYVVNGHSLPLGRIHILGVSDKQVQDNTKFDVNANIGNRDIEKELEAAKTSLEKTQEVLSQVQEALKVADTTLSAKKQAYNTVKTNLEAAQKVLSQAQSVAVQTPTAQAHLVQAQDAYNQAQTRLEKANEAVANLNADIKQKKENLDNAKQLLATRQADLQTKQEALTQAQATLTQLQADLVSAQNAVVTAKDNVEDAKEQLVQAQAYLESLQHAPERLAEVKEKEAMAKSNLVKALDQLEAELTTLKALQVKQLSAQTVYETTAKAYQDVLDAQHKQQLQDEYDALVSQGKTPVPIIDETGKIIGYQAETVQSTTTTQTSHQSLTPIANIPVQKASVLPTTGDKGSSVLAVIVGSLLTLFGLVDIRRKQNSK |
| 4 | MTTKTNGHGYFRKSKAYGLVCGIALATAFLGAHVSADEVTATQDSTVAQTDVSPSVTTSVEVETPSTDTTPPATGDIDETPKEDTTSSDVTDSTDEVVETPKQETDLKLPEATTPEQEKINQTVEDTKDILDDAGVKVEVNGENHYTDPNEAQADADNQKVEAEKFAETQKAIDEAIKQAQELAQKAGLTVTLKPTTVTSIDEAKAKLAEVQKLIEEATNKLIADKKAETEANKLIEEAKKKAEAAGVVITTTEKQNYDKAEDATADAESQKDKLEAFAQMQQSIDEAIKQAQVLAQKAGLTVNVSTVKVTSIEEAKTKLAEVQKLIEEATNKLIADKKTETEANKLIEEAKKKAEAAGIIITTTEKQHYDKAEDATADAESQKDKLEAFAQMQQSIDEAIKQAQALAQKAGLTVNVSPIKVTSIDAAKVKLAEVQKLIEEATNKLIADKKAETEANKLIEEAKKKAESAGVIITTTEKQHYDKAEDALADAEAQKQKLEEIINAQNSVNKQLQDLMASLNAVTQTITGNKVTVSSIEEANKKLAEIKAKIQAVDKLNAQLKAEYDAEVQRVNEHNAQLKADYEKKLAQYEADKAEYDKKLAEYEANKGKDGYLNQTYVQGLIFKSEADAHVTIVKSDGAIIVNDSTDSHRVVLHQGESVTVTYTNLKNSYYNGVKIDKVVYVYTAKDAVNGLHISNNPNITVTFMSSDFDTDDKNGEQNGSQSSHIGMSIQFFDEKGQVITFNEKNPALIAFNSLNKTEVYAGSGYGESIHNLSSNIKIETIAGSSVIYKDGVLYAGNYNDYVSNGSRFDANPATDPNSYWDGDTQANRWYGAAVGVVSSGDTISFDVVMDAGADAKRHEYGKFWFAFSSDVAAPVLTPPTPPEVPNYKKDPTTPPDYQKVNVPTIQIKTDVHEVGINKTTSIDVQTPQLETTVHEVGVNKTTEMEVETPQLETDVHEVGINKTTEMKVETPQLKMDMHTVAYDKPATPQVVKSSILPTTGDETSTASILVSVVGVFMTLVGLVGVRKRKEN |
| 5 | MAKLRKILIALLLLCSSIFSARVAFADTVDITVSNTSLSTNAINGGTSTEFSFDFAVPNSAKSGDTTVISLPDELNFQRNQTFNVYASDGTTVVATAVIDTTTKTLTLTYTDYVDTHDDVTGHLSMNVVVDRTVVTEATTVPATVTINGTTTITISSGGINYTVSTGDSDDIDFWKYGVSYSDDEVMYLINVNTSAATVSNVVISDTINSTGLEYVDGSFEIFEGTWYKNAQNYWALGGSTNVTSNYNIELSADNTSFSINLGTISKGYMIRYRVKANYTLINGEQLSNSATYYSENTALNNADNTFTYQGASGTASGYNYSLTVQKVNEAGEALAGAEFTVTRESTGQVVGTITTGSDGTATISGLLKDNYIITETKAPTGYAIADPVTAEADNSTVTVTDKKATVEVTGTKTWDDNNDQDGKRPDSITVNLLANGTVVDTKTVTADDNWTYAFSDLDQYDADGNEIAYTVSEEMVDGYTTVVDGYNITNTHASETTEVSGTKTWDDNDDQDGKRPDSITVNLLANGTVVDTKTVTADDNWSYSFTDLPKYDNGNEITYTVTEDTVADYTTTYDGYNITNSYTPGETSITVTKVWDDNNDQDGIRPDAIQVQLYANGEKSGDVITLTVADNWTYTWTGLAEKANKKTITYTVEEVSAVDGYTATVGEVENGNVTITNTHTPTTPETPSSDEPTTPSQSNKKSDKEQDKNIIAALLPSTGSRSGLGLTILGLVLVIALLAGLVYHKVKKA |
| 6 | MFKKSKETFYIRKLTIGVVSVAVAGLLVINNAQVNADETTTDMVEQVVTDTELTESDLIQAQADQDQAITEAQAAGVEVTTEDTVTTSDLSEAVTNTVADTEVIQDATAQQEQNNADYAVAVENYNQEVADNQAAYEAAVADYEAKTTEDGYLSEAAGQSLIFESGSEPNTVAEVDNNATAVDQATALAWVEANAVEDKDIIANDIISNPNYISSGSYYFLQQGISTVVTYNNLENTYFNTTKVASVVYTYTLESQESGDQVLVKFFNDPTNTIDVISLNQTTQIGMDVQFLDENGEAIDVSGALLSFASLNSGAVYDDESESYVLNSDEYVTNFDGDYIAINGSSVSYNASENRAYAATSNESLEQGSRFEQNEWDGTELSWYGAIVGVVGDDGEIHFSFGSNNRSTQWFAFNSSVKAYSVPVLETVEEPTLQVVSVSYTPYQYQAPTSSLDDIIVSEESLDDPETTVLNPTTDDISADVDDEINTDDEVIAGDTAVIVPRQEAIDNALDPVSSVTVSDAYIADTLPKTGTEKQSLLAILLGTLLTLSGLIGIRKKADK |
| 7 | MKKVSRLSFLEKRQFFGIRKLKVGVASVAIATALFWSASLANSVSADQISAETAEQAIDVAQSEIDTLADTQTEVDTQIEVENTESIVSEDNTQTAAAETDVDQAVEEVATIENVAEVNVQEASNDTTSLEQLATDATAENTTQTSEVTLLNATPETAENTTPAIAEDTIRVHFQEVTDDNFSQYSLWTWGAVAEPSDGNNWPAAATAFSENQKDDFGYYIDVKQTASHGEIGYLLLKNGEKISDSDQNITPLSQDVNEVWVTSDFKTYTYKPLADDSIIRINYQRDDGDYDGWGVWVWGDVAGEFSTWPTDALDFTQEGAYGRYVDVPLSKLLDSNIGFLLVNQNDPDGVGNKTIDLSFSERDSHSQIFLHNDDTTVYTNPYYIATVTGQDFSKATPGVHQVTVSASSYRSFNYNETDLIDVVVTNPENVDITRMQVDTTAIGGGIIEISPELNRVTITATSDTTPGDYDLPVRVYDSDNGYYDTTVTVTITERVKQEGELDWDEQVIYFMLTDRFYNGDTSNDNPYNQDYAGAINQAGTYKGGDFKGVTAKLDYLKELGVTSIWVTPIVENIPQNVGTEEGMEYYAYHGYWASDFEALNPHLGTLDDFHELIDEAAERGINIIVDVVLNHSGYGTEETFAGMVRTAEEDKGDDIQGSQSGLPDFKTEEETVREQLVAWQTAWLEKSTTAKGNSIYAFRVDTVKHVDDTTWQYFKNELALKDADFHLVGESWGASYKDTKGDLGTGSMDSLLDFGFKDIAKLLVNGNLKQANEELIARNDVLTSSYTLAQFLGSHDEDGFLYSIGGDLSKLKVAATLLLTAKGQPVIYYGEELGQSGANNWPYYDNRYEFDWDDVEGNDVLEHYQKLLAFRRDNSELLARGSHSTVAVNDSQQWLLAKRENDTDAAYILYNLKDESQTLRLLLSDDAVVTDYYSGQTYQAVLGEDGAYYLDLETPAISAGGTMLLKVSTGAITSAFIVQTEEEPIAENTIRIHFKTLPTDDLSSLGLWTWEDVETPSESWPNGAINLSNLKQDSYGYYIDVKLVDGNRQKVGLLINNTNGENLTGDKVVNLISQEMNEVWFDENYAINYYEPLEEGTIRINYYRSDGNYDNLALWLWGSVDSSVTDRLGSWPDGIDFENVGKYGVYMDIALSDFNELGFLLLDESKEGDDAKIQPDNYSFKDLANHTQIFLKDEDKTIYTNPYFVSTVRLTSAQQTSPTEIVAIVSNLADADKDDLFENVKVTDKDGNLMTISDLVLNPEANQLTIVGDFSQLLAPYTVSYAGDDYQAKTNWQYTDSLYAYDGELGARVSEEGARVDLTVWSPSADSVSVVLYDKDDQTKVVGKIAMVKGDKGEWSAALTQESGLGVSDYRGYYYHYEITRGDETVLALDPYAKSLAEWNSDLIGTDPSYKVAKAAIVDTSTIGNQELTYADISGYTDREDAIIYEAHVRDFTSDTAISDELQHQFGTFAAFVEKLDYLQELGVTHIQLLPVMSYYYVNELANAERLTDYASSNTNYNWGYDPQSYFALTGMYSEDPTDPAKRIEEFKNLIAEIHKRGMGVILDVVYNHTADLAILEDLEPNYYHFMEADGTAKTSFGGGRPGTTHYMTRRLVLDSIAYWVDEFKVDGFRFDMMGDLDAETVQMAYDTAKALNPNIIMLGEGWISYAGDANDSRQPADQTWMSQTDSVASFSDDIRNLLKSGYPSEGAAAFITGGAKDLQTLFNNIKAQPSNFTADDPGDVIQYIAAHDNLTLHDVIAKSIQKDPAVAENEAEIQRRLRLGNLIVLTSQGTAFIHSGQEYGRTKQFLDENYKTPVSADKVPAKSDLLTNADGTPFEYPYFISDSYDSTDAINHFDWAKATDSETYPENTQSQAYTKGLIAIRRSTDAFTLKTKEEVDRNVSLITIPNENGVGQEDLIIAYQTIASNGDIYAVFINADTVARDFVLTDDYRNLLSAEIIADGKQAGVEAIENPSGVTMTETGLTIEPLTATILRLKKASAETERTLYDEASGVSVILGAGERDDIKKIKVSHQETNDIKTPEVLKGTDYDLFDIQPTDNFGNVLAITKTARVILPIDAGKTVVQVFYLPENGQPQSLAFEEISRTVAGQTLKFVIFDTEHFSQYGIIYAQEATQSSSDMATQTSAETGVTPDDQAQASVVETTTTPITVDANLETLSGTDAADTLPATGEKDGTLLTFAGVLSLASIALLELKRRKKSE |
| 8 | MKKLKLILATLLSILFAFTGVKAFADESTYTLTINGTTTEHTYEAYQIFSGDIYTTTDENGNEVTTLSNIKWGSDVNAFTFNNSSDAATIAESLDGASDDSDVAKTFATTAAGKLKTPTKTVTSTANSTTFTGLTAGYYLVKDEDESQNGKNAAYTRFILQVVGDATAQLKNDTPTVQKKVKDINDSTETALTGWQDSADYDIGDTVPFQLKATLPKQIDNYDHYYVEFTDNLSAGFTFDGITKVTADGTTLKESDYQLTTSKNSDGSTKISVVITDVKAVATITKDDDIVVEYNATLNSDAVIGSKGNPNTVKLTYSNNPNNTGNGKSKPDDTGETPEDKVIVFTYKVVVNKVDKEKKPLVGAGFTLYKMVNGSKTKIKEIKANDSTSTFEFKGLDDGAYILSETTTPDGYNTIEDINFVVTAEHDVEADNPALTSLSGKLTTGDIVFESDLSEGSLTAEVVNEKGSKLPSTGGMGTTLLYVVGTIIILAAGALLITKKRSSVK |
| 9 | MRKFIRIFLVLLTTLLTLTGISAKAEEDVYYTGYTSDISLNSSYINPDPGPYAIDEGGESKLAYCFNRNKSRPPAKSEPEDGEAKYRKIADVDYVRLKENCSSDMEGRELYDAIMKVIYNGYPNNCSGINGKYRLKDGDFCAITQWAIWHFTDGADSDGTGNLPYYGKESMWNRSDVKEAYLELIDVANLSYPADAKLNLYIYDHGAEHDRQNLLTTDVGYTNLSVEKVWNDSDDQDGIRPAFIDVQLLANGVEVEGQKIELSKFLNSNWQGVFRGLSLYDSDGNPIEYSVKEVEKYRGQLDGYQSTVTKSDSGYSYTITNTHVPETTEISGTKTWDDKDDQDGKRPSSITVKLLADDEEIDSQEVTADTDWKYSFKDLPKYKNEGVEINYSVAEESVSDYETTISGTDITNTHVPETTEISGTKTWDDNDDQDGKRPTAITVNLLADGVKVDSKKVTAADDWKYEFKDLPKYKAGQEIKYSVTEEAVKDYETKVSGTDITNIHTPETTDITVTKIWDDRNDKEKKRPDSIKVTLKANDKDLQTVTITAEDDWKYEFKDLPKYENGKQIKYSVTEEEVTGYTTTIEEDESGNFEITNKIPRDYLFPSTGGSGTISYYVIGLFILLATIFMSIRVRKNKVKL |
| 10 | MKKVIISHYGLPIVKLMVILVVGILVLGISHTVQALTREEVAENLAINVEDGLSSEEYFALGQQYETGDGVVQWYAKALAYYKAAEEQGLVEARTAIDNLNAYKAEVLADGDQGAIFTFYRTGVTASQKGDYEQAFAIYYDDSFFFEDPQDRGLGGLADLYLAGDGVEQNVSSAMSIYEVMAVEQGKGNGYTALGNIYAAATGTYPGVNQSNDTALKYYLLSFQSDNLTATDFKGPRYAANFYDSGFYHDDGTWENPNYTLAEEYYLIAVAGNGRTFDGTAAYKLGTYYEYGREGIAQDYSKAVYYYEKALSDSNVHSTMLGIPDTYLALGRFYENGLGVAIDLEKALSYYYQAQAAAQDNLDLGGNVAGYEAALKVYQEATEAIQRLTQGTNDTETTTENQQTTTDDTTTETTSGDENQEVVTLSDATTGVSVIGTQAALSNAVTVVVMRTTVAALKDLNYDAYDITLQDASGNEVQPSEAVQVILPVTYEVSALYHVANDDSLTSVDVIQNGNKVTFTISHFSIYAVVYKEQIALEETSSDVAVPGPQKKQSDTLPKTGSQSSKPLTFIGVALVMILSVFTINYFLKQKNNE |
| 11 | MKKSKILTMVTVSALVLTQAGTAFADESDIAGTVDDTVVVTPSTTGSDSSTDTQTDTSNAGDNVNDTIDSGSGVIETPTTPTTDDSSSETTPSTETDVVGETTQTESSESNSSEDKSDTQTDSGETQTETSDSQTENDTSQQNQETGTVEVPTTTGETATVNTDKSQATNNPNVSAQTAYDAGASQVGTTSQVTGQVVRDVTTANPVTLASGVTITDIQAGVATLSDGSSANLTDLGVTENADKTFTATTIDGEQVTLPETGESTVGVVVGLLIILAGLGLGFKDKLKALVDKIKVKSKK |
| 12 | MKNNNVVGHGFFRKSKAYGLVCGIALGLAFLGGKVNADEAVVPDGTDVPVVAEASQTIVEPASDELNTAISDAENAGVTVSQTTSETVVNQEEAQADYATQAESLEAVTAQQEQINTENAQITADNQALNEAYESAKAQAESTNQAVSEAQSTYGATVTETTVDYGDGTLTTDYQAGQAQAESIAEANEQAVSDYLTEKAAVDAYNAQVKAREDALKSNNIASDEANYLYVTGEFDTNATGLAYYQNIKVVTLDPNAKTAQSLGWQDNTTISNANGVTVTSHDTANDPAIYGTTSDFLYKVTEATVGDTFTLNNIGKATDGTNINAIVTITKASALTDKEDSWFVIGKTADNGIAVDYWNYDNLGLSFQFVDDSGNAVKLVVASVVGDVDNDQTSKIEFDGNTLNYVNPDGSGLIANADKSLTGLGFAVDGYQQAPQGTYLMVGSSTTVNYTHTSDDNVVDGNGNIVNYIEFDLFGTTSMVTTEEFKYLPDPTLTLTSVTLPTSPVETPLKDNLTATYHLNEYDVALTTVKDVLNDQGISIDGGELQIGETGHYTLEGAKVLANGKDTLVKYDFEDYLDIEHDEYQGYSIYAFVPITLKDGTVIQSGEDLKAYAQAVYDDVTGHFYVSLNSDFLAQVAKDSDFQAKVDIEFVRIAAGDVYNDFTNHLAFEDEDGNVTEVPVPSNEVVTHTVEPPVEEVPEEPQAPTDVQTPEVAEDVPVVSQSVLPETGDNVVLSLATVLSGILMTMVGFLGIRKRKED |
| 13 | MKKLFTILFVLLSVLCFLGGEKGVLAESNSLETFRGYVNNDGKSSESRLGINNIYIDENSQTIVGYCFNIKKQYPSMYGDRYTKYTNVTAEQMKENSSSTLEDAVLYDSVMKVLYNGYPNNGSSIQQKYQLTDDQFRGITQLVIWKFTDSYTGYSFSSDNLENAYNELSNVSQLTYPSNFKLNLYLSTNPYVQNLMAAEMLPENQTEYTSVSVNKVWDDANNQDGQRSSEVTVQLLANGVEVSGQQLVLSDANGWNGTFENLDKYDSDNVLIDYTVKEVTDLSGYQSVVSGSDNNYTITNTHVPEVINLSGTKTWDDNNNQDGIRPESIVVHLLANGVDTGQTKEVSQTDNWTYQFENLPKYQNGQEIVYTVSEDSVGGYETIISGFNITNSHTPETTEVSGTKTWNDNDDQDGKRPDSITVNLLANGTKVASQEVTADTNWTYTFSNLAKYANGSAITYTVTEDSVDNYTTTINGYDITNSYTPGKTSLTVTKVWDDSDDQDGLRPNSIDVQLYANGKKSGDVVTLTAADNWTYTWTNLAEKANKKDIVYSVKEVTEVDGYTTTEGKVENGNVTITNTHVPVIPEEPKTTDVTFSKVEVNGSAELVGASLKVVSGETADGQIATDVKTGENLAWTSGDTAKVFSLSKGTYTMVESQAPAGYELAESITFRVTADGQVEVKGTDGSWTVQADAIIKMEDAKTPVIPEKPQSNTPTVDDPTTPSSNSDKQSEKKQEKKSLLPSTGNSSGLGLMMLGLALVLSVIFGFVYKAKRAKA |
| 14 | MNNLKKILTPFLTVLALVFVCGAVSAQTVDSGAGGSATITINNASQGQTYTVYKLFDATVDGNGAISYKLPSGKTSLGDGSTWFEVDAKGNVTAKENADVSSDAFKTWAASFGTQAATATAEDNTVTFTNLAYGYYYVQSSLGATITVDSTNPDATIKDKNTTAPNIPDDNNGGGKHILVNGETVDSTTAKVGDTVNYQIKFEATNYVTTETDSKQITKYTIIDTPTNLSIDQSSVKVLVKGTEAKAANVSTTFDDAGKMTIVLTWADESGKTIYDSPADVVITYNATVTKGAADAAATNSATIGYNTADNPNEDPTPVDPDKPTDTTEVKTYQFTLNKVDTSSNQLTGATFKLYDAATDGNEIKVVKVSDGVYRVAEASEEGTEIEAGSAIIKGLKGDDTKYYLEETKAPNGYNILTERKEVTISSTETNTSNVINKSGAELPSTGSFGTKVFYLVGSILLIGALIFMISKRRMNNM |
| 15 | MKKFFRREYLAQFKAKYKKVIGAIAVIVVFVTTYALILPALTLDSNAANQTPGINTEQSGSVDDNTTSTSLSTENSDSQAVSETNVSSQDEQLVTTDTTLIADDKNYQVTADITAEAKLPESVALEVNQVKPEDDTYQSKLQKIQDTLSLNTVANIRLYDIAFMQGDAEVEPSASVKVTITQKDSFEANKDNLKVVHIKTDGSTEVLDAEVAGDGNQVTTVTFNSDSFSDFALVDTSTSDDSILNSNGSSTGTDVDASVESTTETTSQSYTVTFYNDDSEITSTQIEAGTALTILPETPFKSGYRFDHWENAETHETVTADTIVTSDLTVKAVFTEISIYTVTVNYYYHNNSANQDVTFDKEIFQLEERDTPYQITPPTSTEVKREEDSTLTADAIYYPQEAVIELKSGDLAIKDELDGNIDGKVTINLQYVPYTAEYTVHYMLKNLTGDDYTEIQSVTNHGVLGSTVSPQVLSYDYAIFEKTEATKLTQSQGQNLYVYYTRNSYTLSYNTNGGSYIPYQTGLYESKVSLTDNVPTKTGYTFVGWHEKEDLSDTAKTSGTITLDSDKTLYAEWKANTVNYTINYYKEVYNNATGTTSYVYDSAVSASGKVGTTVQADEAPALTTVPTGYERESAYGMNANSNVTIAADGTSVLKVYYSLVRYTFVFNLNGPYYYTNGFSTGRISKNGSDYYNSNYTIQNVVLGQDISSQWPSGDDVTDTYNYYSFVGWSSNTTTVLFVTRRHEVTEDMILGADSSNRKTYTANWQTGLITKQVDYYLQSANDPNVYELSSKYSQFYNSQANTMLNPKNIAGFTITETPSGYSNSTGYYYGSTYVVDHYRFYYTRNIYSIDYYYNGSNINTISSIPFEQNINSSTYNYTPERPSGIDADYTWGGWYTDAGLTVPYTFDTMPSHNLVLYAKWVAPTFNVTFDLNGGDGVAPTTQEVEKYKIATSVADPSRQYYNFDGWYTAKEGGERYDWSQPVTSDTKLYAHWSLKPLTYTVRYLDADNNNNQLAADKTVTSPALNYQQVVSESALAITGYHPDANSKSVVLDYDADNNIITFYYTKKSAQVSYRVDYVLQDNPTVKVAESKSVTVDGSTISVKESAVTVDKDYLAKQLDMTPEQLDDYYALTDVESLTLTSSSNNNVITFYYVPYDTAHITVNYLDMDGNPIVGQEPLNTTRKKPSQYTVTHKTISGYTYAQSEDSNGDKNKVNYKIDKGDNITINLYYQKDLYIEAVSKEKNYDGIALENSGLTDLKDSYKELLETDDSLTGISFTGSQIDAGSSAVTPSIALVSSSTGKARNYYYNIIYVAGTLTVNKQSVTVIVNGQDKEKTYDGVAESISYDDLVINDSSGLYKESDIRFTGDKTLSATDAGTYNLTLQGKFTNSNANFEVTFQVSDGRLLIKPRPVILTSATAEKGYDGMALTAQTVTASEPTADTGFVTGEGLLYDVTGSQTATGSSDNTFTYTAMNAATKLSNYDIATVYGRLTVTPTVNIQKTKTDWTALTGGKFEISKWNGNTWTSIDGISDLTITSTDGVTIPVGLNTGRYRITETAAPDGYVILDNSVYFAVTESQAENGQSSFAISLTDENGNTISSLENVKVLEGNSSYSTRIQIANETGKALPHTGGSGRMFYVLVGLALMLVTLAYRQYQKHRERSDLP |
| 16 | MKKLKLIFATFFAALFTFTGGKALAYDITVENGGSGTYESYQIFTGTLSEDGKTLSNIEWGNGITTAGQTALQEKYGVSSAAGLAEVLGADDFTASQAEEFAKVVGQYLQNAGGLTGLAAGYYLVQNASVGNNEAHTNYILQVVKDVIVEPKTSVPTVEKKLKDTNDTTGETTDWQDSADYDINDSVPFQLTATLPDNLASYDEYYLELSDTLSAGLTYNKDAKVYLVNGTTKTDVTSSFTIADDGSSFKINNLKSLDGVTSSTKVVVEYTATLNSNAVIGLEGNPNTVKLIYSNNPNYTGSGETSPTGETPEDKVIVFTYKVVVNKVDQSGNALAGAGFTLYKKDSSGNWNAVSDEITGVTTFTFSGLDDGDYKLSETTTPNGYNTIDDITFTVTADHDVKSDSPALNSLSGDVTTGSLTFASNITEDDASLTTNVVNKKGATLPSTGGMGTTILYVVGTILVLAAGILLVTKKRMDAK |
| 17 | MFYYLGEGIINFRKLFIALVLIIFSFFTVSEVRAADVSNRVTSLTVATTELQDGGRTTVRVEFNDRAGKIHSGDTIEVTWSISNSIYLNGYTKSIPLTIQGVNVGTLEVTEHNAIFKFNSNIETMENVSGWGEFEVIGRNVTNTSSENTGTAVVQVGGYSQNISITKPQSGTGTSSFYYKTGDIQPSDTNRVRWFLLVNNNKEYVESDVTIEDDIQSGQTLDMSSFDITISGYQNKRFVGESALEEFKRSCPNSSIEITQKSEGGHISIRLSRDDVILNTISIHYKTKILDFDQEKFANNSNITYKPLYKDWVTNKESNYEVVNVNANGGVDGSRYTSVTVNKVWNDKDNQDGKRSDKVVIQLLADGQEISGKQLELSEENGWSGTFEKLNKYHSDNTLITYTVKEVTDLPDYQTTVSENSKNNYTITNTHIPEVIDLSGKKIWDDNNNQDGIRPETITVHLLANGVDTGQVKTVSKSDNWEYQFKDLPKYQNGEKVVYTVSEDVVVGYEMSVSGMNLTNTHTPEVTNILISKYWDDNDDKLKKRPESIQITLHANGKEYQTVTLTASNQWQYEFKDLPKYKDGEKIAYTVTEADVPNYQLISIEEDESGNWKITNKVEESYLFPNTGGKGISGYYTVGIVLLLSIGLLFLKKFIGDY |
| 18 | MRKFIRIFLVLLTTLLTLTGISAKAEEDVYYTGYTGYSDGSKMSYDFVHVFSYGIEEDGGSIRAYCFNHIKSRPSSKREEGEAKYRKIADVDYVRLKENCNSDMEGQELYDAIMKVICNGYPNNCSSIKEKYRLKDGDFCAITQKAIWHFTDGVDSDGTGDYIALGALHSKKAWDESSVKGAYLELIDVANLSYPAGAKLNLYLYDHGDTSGDVQNLITTDVGYTNLSVEKVWDDNDDQDGLRPTSINVQLLANGVEVEGQKIELSRSSNSNWQGVFRGLSLYDSDGNPIEYSVKEVERYSGDLDGYQSTVTNTKSDSGYSYTITNTHVPETTEISGTKTWDDKDNQDGKRPNSITVKLLADFRYDDGEEFAPQEEIASKEVTADTDWKYSFKDLPKYRDGVEISYSIVEEPVSDYETTISGTDITNTHVPETTEISGTKTWDDNDDQDGKRPTAITVNLLADGVKVDSKKVTAADDWKYEFKDLPKYKAGQEIKYSVTEEAVKDYETKVSGTDITNIHTPETTEISGTKTWDDNDDQDGKRPDSVTVQLYKSVNGSTPVAVEGKTLTITADDKTDDNTWIASFTNLPQFEKGQEITYSVQETDVADGYTASVSGQMITNSHTPDTVVISGTKVWDDNNNQDGKRTATVTVQILKGETVVDEIEVSESDDWSFTSKELPKYENGQEIAYTVKEIAVENYTTKAIEQGEDGKYTIVNEHTPEKITLSGEKIWNDANNQDGIRPESITVHLLANGEETGQTATASEATGWKYEFADMDRYKDGQEIVYSVVEDEVDDYTSQPTGMNVTNSHEPETTSVSGEKTWSDNNDQDGLRPDSIIINLLANGEVVASQTVTADNDWNYAFTDLPKYDNGNEIVYTVDEATTPDGYTSSVDGTTITNTHTPETTEVSGTKTWADNNDQDGKRPASITVNLLANGTVVDTKTVTADDNWSYSFTDLPKYDNGNEITYTVTEDTVADYTTTYDGYNITNSYTPGETSITVTKVWDDNNDQDGIRPDAIQVQLYANGEKSGDVITLTVADNWTYTWTGLAEKANKKTITYTVEEVSAVDGYTATVGEVDNGNVTITNTHTPTTPETPSSDEPTTPSQSNKKSDKEQDKNIIAALLPSTGSRSGLGLTILGLVLVIALLAGLVYHKVKKA |
| 19 | MRKFIKILFVLLITLLALTGIKAKAETYLGYADSSSEPVKCTLVENNEEAVAYYVTYTFLPKEKNQGGSSFTPTMDVNPNWSDASLAERTAIALYWRCPFIKMDRAELYDAVMKVLYNGYPNNYSGIQAKYNLSNDEFRYITQYAILHFTSEGFDPNGTGDATALGFTDQSWIDTWESNSKYKAAYRELINTSNLTYSPYFRLDIYGSRDGIAGACTLLVAKTAYTKVHVTKVWDDNDNQDGVRPDKVVFQLLANGVEVAGQQLELDAENNWSSAFPGTNQEGRLDKYDSNGTPIEYTVKEVTEVSGYGAPVYQAGVSGDYGDHIEYTVTNARKAETTEISGAATWDDKDNQDGKRPTSITVNLFADGVKVDSKEVTADNDWKYIFTGLAKYKDSQEIKYSVTAEAVKDYETKVSGTDITNTHIPETIEISGTKTWDDNDDQDGKRPTSITVNLLVDGVKVDSKKVTADDDWKYSFKELAKYKAGQEIKYSITEEAVEDYEAKVSGTDITNTHTPETTDITVTKVWDDKDDKEKKRPDSIKVTLKANDKDIQTVTITAKDDWKCELTELPTYEKGKKIKYSLSEKDVPNYKNVSIEEDESGNFEITNQIKKKYQFPSVGGSGSISYYVIGLTAVLFALIFMVVRFYKNQVKL |
| 20 | MKKLKLIFATLLSVLFAFTGGGKAFADDSTYTLTINGTTIGHTYEVYQVFNVQVADDGTTLSDVSWGSGVTPFSYAGISSMNPKEIADSLGSEDNSAVAKSFAASVAANLRTATTSVTASTTSVAIPGLTAGYYLVKDANEDSNFFLVAVTKDITATVKNGTPTVEKKVKDTNDTTGETSDWQDSADYDINDSVPFQLTATLPSNLDDYTEYYLEFSDTLSSGLTYNKDAKVYLDNDGARTDVTSAFTIADDGSSFKIDNLKSLDGVTSSTKVIVEYTATLNENAVIGDAGNPNTVKLIYSNNPGSTDKGETPEDTVIVFTYKFIVNKVDQDGKALAGAGFTLYKKVNDDWKQIGDEITGVTTFEFKGIDDGEYKLVETTTPTGYNSIDDIQFTVEARHTEESDSPQFVRIAVTIHGDGSLTSNEAVNAHGWFKFDVINKKGTVLPSTGGMGTTLLYVVGTILVLAAGALLVTKKHVSVN |
| 21 | MTKKQVIASLAVSTLVLSTAGTAFADEVTPLDPTTPSTEVVASDPSSSTDTSSGTSDTGSTTEPTVPVDPTTPSTGDNTGDNSSTNPGTSEDESSDPGSETTEPEIPTETEKPSTEEPSEVEVPTTDGGKATVVPDPSVPTNNPNISAETAQNAGASQVGTTSTMTGQIVRDVTRSNPVTLYNGASLVDIRDGFVTLSSGEKVTPESVGVTANADGTYTAKTIQGDMVTLPHTGEKNTAGLSAIGFFLMSLLGVASWRKHQKHKI |
| 22 | MAVKGVKDVMNKAKVMTATATLAASFGVVTQVHADDTDLATVSNNSEVAQNQATENVTKSDVDEAKANLDTANQAVSNQEQVVSDATQSADKAQTAYDKAQQETSEAQKLVDQATPENIVTVQNDVTEAEKEVAQAQGAEKVAEDVVNKAQEAVSNQSSVVSQAQQSVNDTQAAVDAAQKDVNDKQAILDGTGQAEIIANRDKAQKDVNSAQEQVKQSQSDLTKAQEADANRQEAIDNAQKTVDEANQTVTSTKSDLDAKTATATQTQATEDATQSAVDAAQKDVNDKQAILDGTGQKAILDEAEAAKVDKANKETALTDAQKGFKEAQEADANRQTAIDNAKTAVDEANQTVTSSKLALAAKTAKANQTEQALKNAEAAYKVVENDYKAINTITLSQAYVEALKKYNDYSLSAEERQQILKTLAEESEALAKVNTYKSNPNDDNSTKYQINDLPEDVIKELSLFAADLVNQIRVVFGSPQVIVTESSVKFVDLVTDGYVSDRWNVTEAMKQGQIGHDAKAVNKAAQYFGLPTSSADEESEGGQYYEDWASSQVYDGITFATLKQRVYQAVTDFMFNADEWRHARDIVGGGDVNSQVYFAIDFSRPSTDSGAFAGVHFVSISEEQVSNSSNFNTTAFANTKSAEKVTATYNAAKSALAKAQADNNMAQEAKTAAQLAYNNAISQLSSASASLTQAQSVAIQTPTAQANLASAETDLTKAQERLTKANKAVEDLNADIKVKQATLTSAKATLEATKSALANAQAENSVAQEAKVAAQTAYDNAVSNLSSASKALTQAQSVAVQTPTAQVNLSSAQATLIQAQENLVKAQKAVDDLSADIKVKQANLASAKQVLSTKQATLATKQATLATEQNRLASLQNSLAAAQANVLKAKENVMTANDNLAKAQAYLTSLQNAPELLAQAQQQEATAKANLLAALDNLEAELLKLKDLQVKQAVAQAVYDTTSKAYQTMLDAQEKQRLQDEYNAIIAQGKTPVAIVDETGKIVSYQAEDRQTTIQSSSVESISVKAQVKPVEEVSVVTASTLPMTGEHSSVLAVLFGCLMTIWGLVGVRRKNN |
| 23 | MTNQNTQNGHGFFRKKKWAKGLASGIALGAVVTISSAAYADDATNTVETQPEVTVVSDSNATNLAETQADNSQSHTDLTNQSGEQTGQLTETVTSDGLDQSVSDAKQAGVEVDENGTVTHSSYADAQADLAKQKEAVDQATETQQAVDSAKATAEEAAKAAGVDVSTEASSQAKTYTSVDEAKADAQTQVDHLSEVTEAQKQVNAELPQAVEAATKSGVKVTVTAGAKTSDAKKALENLANQVASLQKAQATQTTITNTMAQAFEEAKKSGVIINTTGSKSYDDLAQALADAQAQVTKLTEFKEAQASVDKAVKQSADNAKASGVTVSTGETKTYTSLDEAKADSAKQTSSLDNAAETQKEADKTIADLTKQAQEAGLDLTKGDSKSYSSISEAKADLAKQEQALKDAQAAKTKAETINSANKQAAEEAQAAANKAIEDAVSKATASGTTVTNAGTIETTVEEAQKIAQEQVAKVNEVAAKNEQIQEENAAKKSAYDSQKAAVDQKNKAAFDRVGLNYTGDYDTDKVTADKFNADEIERVKSQLEKANDGNVLGANDGWVQYVGSNGYTQSNTSQFKKAQTVNGVEIVATGSLEPGKATVYVKGDVNQSHVIANINWGNVAPEGDGLDGNIGTDWAGAYDYNANGTTTQLWKGKILTWYKIPNAITMLDDSVHDAYVLFHTDTSGLASPADEVVFWNQDGAINAVDGYRYGVNDQSDGIRTVIRVDSPDNNDNYLWISLLGDFDIGQYLEADGVTVLGIGGGFATNSGVGGNRVGVDENLGLTYGKNTTPTSALTGFNSAPDGVALIAQYSNEYSTVIRNTAGGNGVAVARTDFGAEAKVRIVTPRHVSISNVEEPTYTETLPVSVQTVHITADQVTVPTSVTLHDVNVQAETHAIKLETEVAPITVETEVTTPALSATVHDITLKTAVHPVEVTQKPTNEKAVTNNDNIDINGKTVAKGSTVKWVLKNNNLLAGREELTAVVMNDPFPAGFEVDSEATAEANADYYTLKTTEDGSYQLVGTAKLLSLLNAHRDQDVEVPGFVFVGKPLNDNAHYDNAFETIYSTAKGYYKVVSNIPSIDTPDSPNPTKTVTDKAGSDIDGKTVFDKEVTFHLITDYSVYVNTVVDVDTMAKSAGIYDDTDDKTINPDITKATVTDSQGSDITAKGTFYDIEAGQPIPAEIQIFLDGAHLTPDGEFVVWIPNDLKDYYQNYVLTGNNVTVNLPVEIIAQPGETAENTFVQVEFGNGYQSNLVFVKVPDVSPEKHVISKDGTVLDGQEVGLNQVFNYKLDGVTVQKDHDTLWQYDGKDKLDLEHDRYTGNWKGVITGTEYTAKEDMTLPYDVITEDGKLIKAGDTIAAGTSYHFIFEFNQDTNDDFINQIVKVTWDAEKGEWAYSIDKDFLNSLGIEGTFDADFWIEVERIKDGEVENTFINTINGRELVAKVTTHTPTPETPVEPEQPAKPVEEVPVVKAAALPMTGDETTLMSVLATLIGLMMTVGAVFGLRKKEKHH |
| 24 | MQHTLKSVCWDSVLSKPTEPTEPSKPVDTNPIENPVNTDTGVVIVAVEDSKPIIQLADGTTKKVEAKEIGADVQKDGTVTVKGSDGKMKVLPKTGETENIALSVLGSLMVLGSAFIFKKRI |
| 25 | MTSQPESTNDNNAYAENANTSEGTLSVDVDNSSVEKAATEASNAGVDVTKEAAKDYGTDTTTDELNDTKSEIANDQEKQVETLEQATATQTANNTAYADAQAAIDANNDYVNNAKDTYGSETTVTVENDSSTATNGTASANNDAKTKAEQTLVNNKEAVAEYVEAKANYDATVSKAEELNAAVEAAANELKNKGVTVTTSSQVVNSVAEVEALQAQNDAAIAQANSDKGAVDALLAQYAADLAAFNNGTNVSISAGVKTQDVDNLTYGNSFMNGTINADGTFTFTHDMNDGLNDTYGTLGTGTLTGKLNYRVESQGNGNINVYLDSVELYTYTYTAYRQNYAVNQNLNFHVYTLGGTEVYSTYHTGNSSFTDTINRSTTLTYEHGVSVGDTLANVGFLNIDDNWIYNTHGQAVISFTNTNRPPEPVIPPEVTANLVTAVNPEKPTLALTSLSNTTNQTIKASYYDYQLNYSPVVEKDRANSDGVDMDGKTAVKGDVLTDTLDISNIYANLKVGDTVTIVDPLEAGEYYNVDDNIAAAVANGWEVSYDAATETFTYKTTYTGEKLIVPTVDWAPAYDAAYYDNTYKVLVNDNLYHVYSNTVKTPTPEAPQPTKEITDTYGNDIDGSTTFDKNIVYHLNTDYTPYKEVTASTDAISKGFAILDDVADGMFTVNEDGIVIRLTDDNGATTTLSTDIADVTEAGGVSTNHTGNDDSEETTAINKLNDLFTMYHVLAGEAISDDVQAILDQSGLSPVGEFYLWVAKDPASFYTNYVKAGKNITIDVPAYLTANTGTVVPNDFYQIDFGNAYQSNLVTVQVPDVSPEKHAISTDGTNTVLDNQTVGLGEVINYLLDGIKIPEHHDTLWQYNLTDIMDTVHDRYTGNYHGEIIGTKYVASEDLTLAYDVMTDYTVNADGSISGTIIKAGDVIPEGTEYAFYFTFNQDTNEEFVNSIVKVTWYEEAGVWKLVVDSDFLQSLGIVGTFDVNAWLEAERIASGEVHNILINTINGVDYQTEVVTYTPDPEVPEPLPETPVEDVPVVQSSVLPLTGDETTPLNIIASLIGVLMFVFGILGVRKRKED |
| 26 | MTKKQLITALATSTLVLSTAGTAFADEVTPIDPTTPSTEVVTPDPSSGSSTDTSDTGTSTEPTVPVDPTGPSSDVTEVTDDNGVTTDHTNDSGTQTENNTEPSEEVPNVDDAGGVVTDHTVPTTDGGAATVTPDTSVPTNNPNISAETAAQAGASQVGTTSQVTGQIVQDVTSSAPVYTNTGATIVSTQNGSVVLADGTITTPEAIGATTNSDGTISVTTSTGESTTLPMTGETSTVGLSLLGSVLAGLSGLFFKKRQTV |

| | |
|---|---|
| 27 | MKSTGIKDVMTKTKTVAVATTLMAGLGVATNVHADDVTTPSQPETQVNQDKATVTQADVTEAKTSLDNANQAVSAQEQVVKEAATTADNAQAAYDQAKENTSDAQDLVNQATPENIASAQDDVNSAQEQVSQADTAEKVAENTVSQAQEAVNNQQASVDEAQSAVNTAQSDVDAAQKDVNEKQAILDGTGQAEIITNRDKAQDDVNSAQNQVAQAESNLTKAQTADANRQQAIDNAQTAVDEANQTVTSTKADLDANTAQAHQTQAIEDAKQADVATAQKDVNDKQAILDGTGSQAVINEQAAAQADNETKAAAVKDAEANLTKAQQADKDRGTAIQTAQAQVNSTTQAVATKKADLDTKQGQASKATQVLDKAKADYTKAENDYKSINTIQLSQAYIDALKVYADGKATAEQKAKATETLKVESARLAKLNVFKANPNEPDYKWGTTEQTYDAENLPDNVVQELSLFGSDLINQIRAAIGTPQTVVTSSSIKLAQLVKQGYQADNWDLNKGHDIEALKKAAETMGIDYPAEDATLASLQGETTIGSLKREVYNSIVDFMFSDYEWYHAGILSCVGDNYASYMKVTYFGFENGVGTHFLFNSNASLHVDSNFDITPIANPYSSDKVITAYNTAKATLDTAQATANTANQALATAQSAYSLAVTNLNKAQDSLTQATARPILTPQATSNLASAKVAYAKAQERLTKANQALASLNADVKAKQAALTSAKAVLATALAELAQAQANNKVAQEAKMTAQTAYDNAVSQLSSVSANLTQAQSVAVQTPTAKANLASAQSTLKQAQENLVKAQKAVDDLSADIKAKQANLEQAKQFLANKQAMLNTKEGLLATAQAKRDSLATDLATAKYQVAQAKANVDKAKAAYDSAKAHLANLQNAPQALKEAQARLATAKTDLLKKLDGLKASLETLQALKAEQATAQSHYDKVSTAYQNILEAQEKARLQAEYDSLVAQGKQPIPVVNETGKITGYTVLAPEAPQSASKAQGEVVTTAKTAMVTPTATKASTPVANTMTRQTANSLPMTGDTTSTVALLFGTLLTLFGLVGVRKKETK |
| 28 | MTKKHMLALLSTTVLLTQGAVTFADEVVPVDPTAPSTGVVVPDTSQPQGTDTSETSPVDPTVPSTLPSTDESTVPSETTPDTGSETETTPTEEEPSVPQPEPETPPTDTGEVEVPTTDGGTASVTPDTSVPTNNPNITADVAHNAGASQVGTTSTVTGQVVQDVTSTHPVQLANGATITDIRDGMVTLATGEKISPEAVGITQNSDGTYTAKTIQGDLVTLPHTGEKATPLLSLVGTALLAVMGLLIVKPWKKVRSHASVFGSRQK |
| 29 | MTKKQVIVSLAVSTLVLSTAGTAFADEVTPIDPTTPSTEVITPEPSSSADASSGTSDSTLGTSDTGASDTTVPVDPTTPSTGDNTGDDSSTNPGTSEEEPNTPSIDPSDPETPGDTEETPVDDPATDQSGTVEVPTTDGGTSTVTPDTSVPTNNPNISAETAAQAGASQVGTTSQVTGQVVQDVTSEAPVYTNTGATIVSTQNGSVVLADGTITSPESIGATTNSDGTISVTTSTGESTTLPMTGDTNTTLLSVLGTGILGFVGFLFKKRQRFN |
| 30 | MLLGFRKSLLDYTNLKNKKELASKVLLKVKIMSEELRKKERQRFTIRKTKAWGACSVLLGLSILFILSPVSVKADDATNSVLENTTVVENPNETLSVDTDDSSTTPENNSDDNFSEAQEESSAENDIKSENMNALSQSALLSNSLSSDANDEVSTVTISYTINYVNSEDNSIVSTKTASVDVESTTDTEVASTTVTEKSEAISGYSIASGTSNIITTTLTEGEENVITFYVTPSESTTKDVTGVETVYSRDTSATTNNDSTVANETGEYWPNTNGKLLSEYAKSITTSFATAQVYSDEYSATPTLVNYSDLLLYVNGEQVDVATLISDGSSGNPSSIATNLFGENTYFYVKNAFSIYNQETGQIDVYSMKWTLQSVNYSTDDTTIGFSVYETGSGRVNTFLSLGSGSQVQQRGNYINSHVQFFKVVDNENAGSVSVEEAIAAGYIESSAVKIHLGYADIDADESVEITENIIKNVYVSDNIALAYQLTDDGFLAVTRKYNDTSNVSETTEGQAYFLMELDVPADGFDISISTVGTKLDASGNNIVGQGSKVAPSILSTIKYPPKLFINYYELGESGNKTTIKLQDSVVGNGYSGDLYTDAVDENGDKISLTPPSTITDTEGNVWVLISDRTDGTKNTRFIDGTTESINYYYALLTGDVIVHYVDESGNSISADILDTENGNNGSSYDTTDNKLTSITSNGKLYQLVGVGVEDSDGALTFTSDGKVITSETSSEEQGYLTSTSTSTTETTSSYDSTTEPSTITVTTENRDETTRELLSITEVTTTITVSAVEGSDENLVTKNVTTTIITIPTVEVTYVYKLVTGDVLVHYVDKNGNTISDDVIDEENAPVNDTYDTTDHKPSEISYNNHTYRLVPSLTEGNETGTVVKGITEVTYVYELVTGDVLVHYVDKAGNSIATDVVDEENAPVNDAYDTTDHKPSEITYNGHVYRIDPTATEGNETGTVVKGTTEVTYVYELVTGDVSVHYIDKDGNPISDDVVDEENAPVNDAYDTTDHKPSEITYGGHTYRLVPSLTEGNETGTIVEGTTEVTYVYELVTGTVVEDIPEVAYVYELVTGNVLVHYVDKDGNSIADDVVDEEAAPVNDTYDTTDHKPSEINYDGHIYRLVPSLTEGNETGTIVEGTTEVTYVYELVTGDVIVHYVDKDGNTISADVIDEEAAPVNDEYDTTDHKPTEISYNGHIYRLVPSLTEGAETGIVVEGTTEVTYVYELVTGDVVVHYVDKDGNPIADDVVDEEATPVNDEYDTTDHRPTTITVDGVTYHLVESELPSNEIGKVSEGVTEVTYIYQKEIIEVVTHHIDENGNPVADDERGTTPHKTPEELPEYEFVKTTTDEFGNTTHIYKKVEKPVVRKTVAPPTDNPQKHETNVKTVINPTDNKAQSNDFLPNTGNENSSMTSLAGMLVTFIATLLFRKKRKDN |
| 31 | MTKKQVIASLAVSTLVLSTAGTAFADEVTPLDPTTPSTEVVTPDPSSQPEQSTEPTVSTDTSSTDTGSSTEPTVPVDPTTPSTGDNTGDDSTVPGTSEEEPSTPEPTETGETETPSQPEEATTEPTEQPGTVDVPTTDGGTSTVTPDTSVPTNNPNISAETAASAGASQVGTTSTVTGQVVQDVTSEAPVYTNTGATIVSTQNGSVVLADGTITSPESIGATTNSDGTISVTTSTGESTTLPMTGDTNTTLLSVLGTGILGFVGFLFKKRQRFN |
| 32 | MRKFRKRLMMLLSVFLAFLGFNVQTVLADNAKELTNVITDIAIWDTSNGRYATQSGGVYQLTQNVSYSFEVDFDLSAYDGNLANGDYFTFTIPEPFTVASTSFELTDEESGVAVGEAVVISNGEGLGATVTITLKNLEEYLEKTGGTEVQGVQGTFYTDFSVTELITEETVTFDTTETTDTITHTIKVSERTSTDYSSVIGKTNFSKINGVLRAKSWTSEALGKSGEYVHPFTVRVNERQASYDTIVVHDYISENYAPAQFIPETLAINASYYNSNFGFVYPFTRLVEGVDYTIEWNSSYTDFVVTILNASTRLAANGQPAAYQITYDTTAPANGTSIGNYVEVTADGQKLTNTTDGTRDYALAVRESKVTSGGTIQLDTGYRITLYKQDAETGNLISGAEFEITSPSGNTETVVIGDDGYAQSQVYSAEEVAKGNFTVVETKAPYGYELDSTPIEVTVGASGAIRTIKNSKYTATANISATKQLTGRELKDDEFEFTLTDSDGNVVGTAKNDASGNIVFDELTFDEAGTYTYTITETNGNLGGVTYDDSTIKATVIVTADEDDNGRLSATVTYENNDNTFENTYVPTPTTATLAATKALSGRALTADEFEFTLKDSEGNTVETVKNTLAGVVTFSPITYDKAGTYAYTITETNGGTTVDGVTYDDMAITATVFVVDDGTGNLTATVTYSDDTEFNNKYETNEASAALEVTKAVNGRDLTAGEFEFTLKDSDDKTIETVTNNANGAVTFSPITYDAVGTYVYTITEANGGETIDGITYDALIVTATVEVTDNGKGELQATVTYSDDTEFNNIYTPAGATTATISATKVLDGATLTAGQFSFVLKDESGTEIETVTNTADGSITFTPITYDESQVGTHKYTIAEVAGTATGISYDTTIQEVTVTVEKVSATELTATVSKTANGVVFTNTYTPAKTEVKVTKNWDDADNQDGKRPNAITVTLMADGTDTGQTLELSEANGWSGSFTDLDADKGGVAIDYQVVETTAVDGYTTSTSGNATDGFTITNSYTPETVDVKATKNWDDGDNQDGKRPTSITINLLADGEKVASKEVTAADDGTWTAEFTNLAKYKDGKEITYTITEDSVTEYETTITDFDIINKYTPKAIEYQVTKVMKKNVDTQQRYAFRKLKFGLVSVAVALLFGISYQVSADETSVVSDVSTEVTVDATDSSDINNTITDGVITDGPVASAIDSISESAETVIVPEESAEVGDVIDVNIDSTEPVITETTDGDKEIATAQATVTVTTTSIADPEEPVGISDPVVEVSEATATDEDDNYTYTQYEKVTKTTVTEIVKETDVVTNVIAPKADVVFIIDHTGSMSDQIEAVKDNIKDFVSGLDAHNVGVRLGLIDYEDSSDVNYIDFSGSRFTTDTDAFIQALEAITIRGGFEEPTTPLTYIASSGDYDWSTDADAKRFAILITDEDFDYYSAGIPSLESTIQALQAAGISTTVIGETYLQDEFTSLVNATGGIFVDIDSDFAQVLSNDILTWIVETIEEGRLLKVVTETYDFYIEVVAVPKAISSVDEVTLISVKEDDNTPKTTVITITDSEQLPETGSQSNAMLSVAGIISLALASGLVGFKKKTKE |
| 33 | MKKIKAVALFSTAILATASTGVYADELISDGTTSPSTEQVTPSTAPSSEVVIPDEGSTTPSTDTGSTTEPTVPVDPTNPSSDVTEVTDDNGVTTDHTGDSDTQTESNTDPSEEVPNVDDAGGVATNHTVPTTDGGTATVTLDTSVPTNNPNISAETAANAGASQVGTTSTVTGQIVQDVTTDSPVYTNTGATIVSTQNGSVVLADGTITSPESIGATTNSDGTISVTTATGESTTLPMTGDENTTLLSILGSALAAIGGLFFKKRQYFN |
| 34 | MTKTCNYHFLVNQEKGEKHVFRKSKKYRTLCSVALGTMVTAVVAWGGTVAHADEVTSSVDTTIQRTENPATNLPEVQPNPVSEQTESLALTGQYSGAIAVTVPHDTVTKAVEEAKVEGVSTVEDSPMDLGNTTSAAETNQQISKAEADVQNQVEAINEVTETYKADKVAYEDEKARIEQDNKELSQAYEEANQTGKETNAWVDSKVNDLKTRYADADVTVKEQVVSSGNGTSVLDYTNYGKAVETIQSTNEQAVADYLTKKTKADDIVAKNQAIQKENEAGLAKAKADNEAIERRNQAGQAAIDAENRTGQAAVDQANQEKQQLVSNRAAEIEAITKRNKEKEATARKENEAINAYNTKELERYQRDLAEISKGAEGYISEALAQALNLNNGEPQAQHGANTRNPNQIISTGDAMLGGYSRILDSTGFFVYDSFKTGETLSFSYQNLQNARFDGKKISRVTYEITNLVSPAGTNAVKLVVPNDPTEGFIAYRNDGNGDWRTDKMEFRVVAKYFLEDGSQVTFSKEKPGVFTHSSLNHNDIGLEYYVKDTSGKFVPINGSTVQVTNEGLARSLGSNRASDLNLPEEWDTTSSRYAYKGAIVSTVTSGNTYTVTFGQGDMPQNVGLSYWFALNTLPVARTVTPYSPKPHVTVELEPIPEPITVTPDVYIPKTFTPEKPVTFTPKPLKEVVQPSLTLTKVTLPVKPTPKELPTPPQAPTVHYHAYRLTTTPEIMKEVVNSDQANLHEKTVAKDSTVIYPLTVDALSPNRAQTTSLIFEDYLPAGYLFDKETTQKENGNYVLSFDETKNFVTLTAKENLLQEVNKDLTKVYQLTAPKLYGSVQNDGATYSNSYKLLLNKSTTNAYTVTSNVVTVRTPGDGETTTLITPDKNNENADGVLINDTVVALGTTNHYRLIWDLDQYKGDRSAKETIARGFFFVDDYPEEVLDVVENGTGVTTLDGQKVSGITVKNYASLNEAPKDLQDKLARAKITPTGAFQVFLPDDNQAFYDQYVQTGISLALLTKMTVKDSLYGQTKTYTNKAYQVDFGNGYETKEVTNTLVSPVPKKQNLNKEKVDINGKPMLVGTQNHYTLSWDLDQYRGIKADNTQIAQGFYFVDDYPEEALLPDEVAIQFVTSDGKTVSGITVKAYSQLSEAPKMLQAALSKQKIQPKGAFQVFMPEDPQAFFESYVTKGENITIVTPMTVLETMLNSGKSYENVAYQVDFGQAYETNTVTNFVPKVTPHKSNTNQEGISIDGKTVLPNTVNYYKIVLDYSQYKDMVVTDDVLAKGFYMVDDYPEEALTLIPDGIQVLDKDGNRVSGISVSTYASLSEAPKVVQDAMAKRQFTPKGAIQVLSSDDPKTFYETYVRTGQTLVVTLPMTVKNELTKTGGQYENTAYQIDFGLAYVTETVVNNVPKLDPQKDVVIDLSHKYESLDGKEVALHQTFNYRLVGALIPSNRATDLFEYGFEDNYDEKHDEYNGVYRSYLMTDVILKDGSVLKEGTEVTKYTLQQVDTENGLVSISFDKSFLETISDGSAFQADVYLQMKRIATGQVENTYLHTVNGYVISSNTVVTHTPQPEEPSPNQPSPPQPPIETIAPPVPASVLPNTGEQESLLGLIGAGILLGTAYGLKKKEEK |
| 35 | MTKKQILSALALSTLALAQAGFVLADEVTLVDSSEPSTEVVTPVEPSAPVETPTEPTQPVETPTEPTDPTEPVEPANPTPPVEPTDPSTPPTTEEPAKPSPDPVVPTPSDGDGSSNPGTSDPDPEPKPGDPVPVPAEDPISSKPISEPTLPFNPFKTQGGQTIVGTQNSQVIIQQADGSTQTVKAEEVGGTTNADGTVTLKATSGEVKTLPQTGEEAGGLFSLLGTVILTSLGFLKKKKII |
| 36 | MNQETKQIFSLRKFKTGTHSALLAKYGIALATTAVLMGAGSVSADETPPASTEPSTEAVAPTIEQPATTEESPVQEAPAITSDIPTSVPATVTQDQAVTAVEDAQDTLKGNVDTAKNTGVVVTEGEPQEVVIDDANAAEKTSEVLTDLNKQDQAVKQATAKQADNQKAYEDTKAEHEAVTKEGQESLDKSTVEVDQLIEQAKKAGLIVTDTTTDQTPAYKDIKGLTGDELRKAMSENLDLYKAAITAGITIQDKSVADLKAALALYQANQKAYNQANTALKDAKSAGVADLEGADGRQDQVIDLAKKNDVPINVKSETVTPKYTDVKGLTGDALLKAINDNIALYTQAVADSVAKQDGSTKDAQAKLDAYLKLLADYQAGKGQGSGIKWAANTTVVAGTAQKMTGDEPAVDWGNLRTAAADAIQSLDRNQNTDAKFDNLFKINGSGTVTIKNTTNGDVTLKISNIQAWQATATYLAVWGAKDGGIAWGVFATTNGTVAGGAGEGATGGGTIGYSGNILAMVKSYDYEVSTSNAVSTVTFNDIDNNQTVTLNGLDGSKVVKGRNVQQSGNSYSAGAGDVSQASGGVIDSNGVQFNFEDAKKVTFSGSHFLPGHINTSIVAGIFGVASEVPKEPKKPNLEVTNLDVTVPEAPEAPEAPKVAVHKVTVKAPEAPEAPAPVEVSVHYYKMKTTPAPEKPVTPPTPNTPVQPAAVLPLTGEASSVLSVLGGIILSGLGLAGVRKRKED |
| 37 | MTEYNQAQKEAYDKAYAQYLKDLEVYNKKLAEVQKHNEEVKKQEEEAQKNNIASDKQNGIYVTGQFNKDQTGLDYYKDITIMSTNKDAVGAESLGWQANTTISNVNGLTITPHDTANDPAVHGTTSNFLYKVTEATVGDSFMLNNIGKTSDGRTINALVTIAKASILTNKELSWFLIGYTADNGIAIDYWNFDSLGLTFKFLDDNGNELRLVVGSVVGDVDNDQTSKIEFEGNNLHYVNPDGSDLVQNADGSLTGAGFATDGYQNAPQGTYLMVGSSTTVHYTHTSDAGVIDGNGNIVNYIEFDLFGTASMVNTLTLIPEPQKPDEVTPPTYQTMPDKPNYQKVEIPTIQEAPEIVKSSVLPMTGDTSGSIVATIAGFFMTLVGLVGVRKRKED |
| 38 | MSESVSESLSEINHSDSRKGSMSSTPSQTRLPNTGTSSSKAELFGSSLLLSGLVLAAYKKRKKDETEESSK |
| 39 | MKLSYEDKIQIYELRQEGHSIAHLSQQFHINRSNLTYMIRLINRMKKILSRHKFFIGLCTLFTIIGLIGTGSDLVSAEETNLVDVTITDFRIENLKHEEPASIYYTDRFYLAMDWDASAAGADIHEGDYFDVTLPSNMWFPSDTSARNFDLKNQEGVVIGQAMVNPGLNDIGGSIHVIFTDAVENKYNVKGTMYLASKFDKTQTQIGQDNSFSITVNGQVIITEVNITGPTLVGDEILAKWGWGEITHPEKAGWSARINTNGKNLHQVVISDTLGNSGMTYLEDSFKLQKVKFNEYGQISVSLETISGETLKKQGKLKFENGGTSFILTLGDITDGYYLDYSSTYIPGTTLTNKITLTSTEESKTIIANHIGAESGGTGSGDLASKIKITKVDADDNVTPLANAVFEVTAPDGNTFNLTTGADGTVTSGVLKQGTYTVKEVTPPTGYELNTDTFTLEVTPTGGALQTVKDTPIKRSIKVDKKWIGKEGGPVEVILKADGTEVQRASLDVAGNWTHTFDNLREYNANGQKINYTVEEVSLPNYDSAVSGDMDHGFVITNTNNEKVSISGTKTWNDNNNQDGVRPDKITINLLANDKKVDSKEVTANDNWAYSFDNLAKYDDKGKEITYTVTENPVDDYTSEQKGNDFTNTHTPATTQVSGTKTWKDNNNQDGIRPDKITIHLMANGQEVHTTEATVANNWSYSFTDLPKFENGQEINYTVIEDQVPGYTGEQNGNDFTNTHTPATIQVSGIKTWNDNNDQDGIRPEKITVNLLANGKKVDSKEVTANDNWAYSFDNLPKFENGQEIKYTVNEDAVKDYTTEIIGNNITNSYTPGKTAVNVTKVWLDNNNQDGIRPSEIKVQLYANGKAIKDKVTTLSAANNWQANFTDLDIKADGKVIDYTVKEVTVPKGYKDKVTADKSNNVTITNSYTPATTEISGTKTWKDNNNQDGIRPDSITVELLANGESTGNTQTVSVDTNWEYIFKDVPKYANGQEVIYTVREVNIPEGYTSAANGMDITNMHTPETTEVSGTKMWNDNENSVGLRPDSITVNLLANGIKVASQEVTAQSDWNYTFTDLPKYANGNEITYTVTEDTVANYTPTYNGYNITNDYTPGETSVTVTKVWDDNNDQDGIRPDAIQVQLYANGEKLGDVITLTAADNWTYTWTKLDENLTYTVKEVSDIGGYTATVGEVENGNVTITNTHTPTTPGQSDNKSDKKQDKNIIAALLPSTGDSDGFVLSIIGITIIIVIIAVFVYSKVKKHN |
| 40 | MKRWGLLVVSCFLMFLPHIVSAQEQQTTLSLTVTNTVSENQDSTTPFTFQIDGKDNAPMPETNQITINGSGKSSFGRMIYDNVGTYYYTVQRLNNGLQDYSDDTSIYTVQVIVTYRLDNPDQLIATAIAYKSGEDAKEDLTFQSVYTGKITETSSSITSNIKTDSSQKGWKRLLPNTGQSKNLLTIIGFACIISVAIITIRYNRRQK |
| 41 | MTTNNTLGRAQGLVSGIALAGAVAFSSGAVLADEVTSPVTESEATTVVTQPESTNDNNAYAAQANTSTGTQPVAIDNSTVEAAVETATETGVAVTKEATVDQGTDTNSTDLDASKADIKSDQEKQVKEIKAATQTQADNNAAYSEAQGAIDANNDFVEDAKGKHEKDTTVTVTTDGSTATDGSSAKNKQATEVAKKTLKGNQESVKTYELNKATYDATVAEAKTLNQAVESAANALKDKGVTVTTTEKVVTSVAEVEALRKQNEAAIASAEKQITLNTAILAAHDKTKKASDATNADADSKVTDLKEKGVTVNVTTTVVSSAEEAETLRKQNEAAATSAGNQVASNNAVLAAYDKTKKASDATNADADTKAKELQGKGVSVTTAEKEVSSAEEAESIRKQNADAVALAEKQVTSDNAILEAYNKTKKASDTTNADAEAKVNELKGAGVVVTTSSKEVSSAEEAEKITSQNKSAYDQAKQKQADWQKKYDELQSKTGTEGYTKQVVLQALDLSASNPQATHGSSAAGSQIVGTRDIGSTTGSSGYGRILDSTGVLKYANVGKGWTTEIDYTNLKGLTVTTTDGQKHDISRIHRTFQLVNTGATGLNDVYIPNDPTEGFIVARNNGTGSYSDYMNFLVTDSYYYTVNGKEVVYSASQETPIALTYSSLNNNPIGREGARATGDGSSMVEINGSTVSVHGDQFAYSDNYNRQEETGLNWDTTDSAYQYKGAAIGVFTQGSSFTTEFVQWDGSADPAGQTYWFAINTRVVAPVVKTPTSARLTKTTVKPTEVKPISASLIKTTVKPSEVEPVNVSIVKTTVDPVSPDPVSAELVKAKNPEKPTLELTKLADTKNQQVSVAYHDYKLSYKPTVTKTVTDTDKKNTDGATVAKNTPQVYTLDHDNIYANIKVGDTITIVDPLEAGATPVVAENEADAQAKGWTVNYDETQETYTYTAVYQGKKLEAPTIKWVPIYDKAFYDNTYKVFKNDYEAFSNTVTTYTPPTPDPVKSITDNSGADINGAKTFDRNVNFHLTTDYSPYTKVTASTLAIAKGFAIMDDVQDGAFTVNEDGITATATDGTDVKDLFTMYHVLSGEARTEAIQAILDNANLSPVGEFYLWVAKDPASFYANYVKQAKNVTIDLPARLLVPEGEVVENDFFQIDFGNSYQSNLVTVEVPDVTPEKHALDQKDDNIILDDETVQIGEYIRYLLDGVTVPVKHDTLWQYDGKDKLDIAHDRYTGNWKGIIKGTEYTAKEDLVLTYDVTLEDGTLVKAGDTIKSGSKYAFTFEFDQDTNNDFIKKIVTVTWDDKKGEWAYSIDEEFLRSLGVEGTFDADFYLEVERIAAGDVENTFVNIVNGKEMTAKVTTHTPEPPKPTPPAPEPPKPTPPAPNTPVKQASLPSTGEASSILTVLGGFLLSGLSLAGVRKRKEN |
| 42 | MKINAVDLFCGVGGLTCGMQQAGINVVAGYDIDARSKFAYEYNNKAKFILKDVKEIDDTEIGNLYPVDTDIKVLMGCAPCQPFSTYSHKYKNNKNTIQKMDLLNYFGEQIEYVQPDIVSMENVPQMVKEKQSSVLPETGEDTTLSLATAFVGMLMSIVGLFGIRKRKKD |
| 43 | MKKLGIKLANLIKKRVQDSIIAKIALVLSVAIVFCTTYLLILPALTISTDSSSSVIQSVSSSEETTTEALQEESSQAESSEASDSSTTTSEAETETSDSVVPGSLSAETSDVTVTVTYEDDTFSEPVTLKVSPVSDTSAINDKLTSVLSETKQTLAQAYTYDISFVTASGEEVEPSKDVNVSIAFKNAVSSSELGGNWKLYHFVDNDVNKVEDLTGQTDTTINQDSSENVTSVDFKSDSFSNYTVADVTYADFSDYLTGYSYDESSVQEDLSTEELTISLKLSFKINQTDLTSEKNYYLALPDDTSIGADITLGTEYTGRDGNGTEAYKYKFYYDESTGKYYILVSFDDSYVSSMGSGVESTGYINYEAIFGLEHKTSDGGYTVVYSDDVKVEIPASDITKNYDLTTSKTGTVSYDGDTPYITYTITVDSKYGTPGDIDLTDTLTASGISIDSVDNVSITKSTYAGSTSNVTSTETVSKSPTFDTANGTWSLTLDQLTSGGTDSDGNAIGHFYTITYRYKVADLTVGEDVKVSNTVNAVSKDDSDKRETSSSTSVDLKLNEVKKSGKYDSSTGKITWTITVNPNGNDIAGAILKDSFLSEASDLTISPSNGYRIYNTSGGYWDGNDTSDIAYIRFTGSKNTKSYTITYITDVTDTGWSSSKVKNTVTLDDDGDTTTTDDQTSDSSSPSVPGSGDLTKSFVSEEDTDDSDVKVLNWKTEITMPDSGVIEKGTTFTDVLTKPYAQNSEIHWYSKSQLEDLYTQLVSVFGEGNFTLKARQRSSGSYTDYANLDDSVNYTEFVITLTSDFTSTDNVSFEYASTAKLSGGATLDFRNTITSGDHSSQVDYHHTSNPNVVKMDGDENPATSTKSSTDGVITWKVKVNLSDDATSMTVTDTLPEGVSLTGLTYGQHWGQISAVISGTSITSGENGWGTYNIDLTGSISDDNVVTLNFSTTDGQTLKNNIGGNNDFWLTFTTTYNDLPEEGTVVTDTLTNTVSVIVDGDQDYGSDSQTQNVTIGKEKTETTPMTKTGTWDNNNRALHYSLSINSSAEDLVDGTDELTLTDVLTFYGADISVALNQSSVKLYYADSGEEVPTSEWSWKVSLTDNGYGTYTSTLTVTLKDATAYTLAYDYLVSKENPDTSSTYWPTNTATLSGLSNGSTGTTTEVAWSKIGTDAGIDTEKSYTINKVDADNYGLGLANAVFTVYKYDGDDDDSNDEAIATYTTDANGHITITKSNLPSTFTEGNFYYFKETTAPSGYELSDSSSKYYFYYDTENTITPTGVLSGAVNLAKVSDSAYVENEKIKTIDLTVDKKWFTADGDETSRSDGSITYDVIQVATAEDGTSTESTYKSGETLSHDDNWTKTYTELPVSGTDTSGNAVTYTYYVKENAVSGYDTSYTNSNGDSSTTAADMAIASDSAEITIENTAQKQYDLPDTGGNGTQWHYIAGAVLSLLAIGLLIFKVYKRYQIGGHL |
| 44 | MYNHNRILEFNFLNEGDHILASSDNTNLKLLKAFSELLKVTNFDQIKVSDLVKEAQLSRQSFYNHYRSKEDFLRESILVIFDDITKILNNNLLYDKVILQEMLNYIYINREIIKPLVSYFPNIDNIIIDYIKDMIIHCTDPKKMIRNHRQKNYDIAHTKQHFSIKKFKFGVASVLIGLSFLGGVSQGQFNTAVSTVYAAEVIPGSAATLNASMTKNIQNGRAYIDLYDIKNGEIDPLQLIIISPTTYSAQYYIKQGNSYFTDPSNLTDTGSATISYTIYEENGQPHKKPDGQVDIVNVSLTIYNSSNLRDKIDEVKKNAEDPKWDEESRNKVLEALNDIKIDIDNNPKTQTDIDNKITEVADLEKTLVTPRTDADKNDPTGKDQQVNVGETPNVKDQQVNVGETPIVNHESNKGSRLPATGEKESLFFNITGWLVLTSVVLLSILKKKEE |
| 45 | MLTQAKARLASLQADLLMAQENVVTAKANVDKAKAMLDEAQATLANLKNAPQLLKEAQAKIGYHIPQTVVAHPTTAQVVSKASKAVETPTAFKATVTPQSSALPTTGDSKSVLAILSGGLLTLFGLIGVRKRKEN |
| 46 | MTTKQLSQSTVNGHGYFRKSKACGLVCGVVLATAFFGAHVSADEVTSSEPISIATEQEANQTIVAPENDKLSDAIQNATNSGVNITQDANQSVGNKEEANNDYDHQAEELDKITTEQDKINQENAQINKDNQALEEAQQNANQAAQNTNQNADKYHEQHGGTVTDTTIDYGDGTSTDDYHKGQQEAEKIDKENDKAVTDYLNEKAKYEAIVEQANALNNAISKAENDLTSKGVTVIKVNKVVHSLEELKQLLAQNEAAIAYANKQVTLQLAIMKAYNDANDAYQNLNQQVSDAVTNHGGSISVDKVEINTGQSESDYQDYKATVEDLIAQNQQKLQEFLQKLEEFKTSSQVNVSTSVATQNVDNLTYGDSFMSGVINADGTFTFTHDMNDGDNSVAGILGTGTLTGKLNYTTVANGDGSITVKLSSIDLYSYQYTSNRLNHAVNQNLNFHVYTIDSVEIYSNLHNGNNSFTDVINRNILLNKVYTIAAGASTGDIGVLTIDDNWIYNTHGQAIVDFTNPNQLPIATLHSVKAPVNPPVVEVQAELVKASNPDKPELVLQKVNEAPDPQKTPLKNNLTASYHLNEYKIPLTTVKDVLNDNGISINNGTLQKEETGHYILEGAKVEAHGKDNLVKYDFEDYLDVKHDEYKGYHIYAMVPITLKDGTVIKSGADLQAYAQAIYDSITGRFYVTLNSDFLAQIAKDSDFQAKIDIKFVRIAAGDVYNDFVNHLAFQDDEGNVTEVPVPSNEVVTHTPEPEVPATPETPVEDVPVVQSSILPLTGDDTSSTSILASVAGILMILVGLVGVRKRKED |
| 47 | MTKKHQLLAALTVSTVALTQAGLASADEVSMPETFNEVSVVTETQPVDPSTPIVPNVPATPDVPTDPETPPTNPDTGGGDTGGETTPDQPTDPSTPPSTDGSDTDNSGGDNNSGTGVDTETPSNPGTGEDTNNPTEPSQPTNPPITDDAVNKDDQGNITVKPQVVAPNQTVVGTQNGQLLVQDTSGNTRLAEASEFGGQKNDNGTVSFKTADGKTVTLPETGSTNGTLLSLLGSGLLGLVGFFWKKKQLLS |
| 48 | MRKIRDYFLLLLTPIIVFMGVLTTTTGQIVHAASGNELTNVITDISLWDVNNGQTLQPNSSGTYNLVKDNSYRYETTFDLSKYDGKLNDGDYFIFTVPAPITVENTSFDLEDKETGVAMGTAVVTSSGDSKGGTVNVTLKNLKDYLAKKGGSQVQGVKGTFYTDFKITKVTKSETLTYSTNQTTKEITHTISVTERGTSDYTDTIGKENFAKIGGVMKKESWSSEILNKSGEYIHNWYLRVNTNQSTYDSISLKDAIPSDSAPMQYIPEKLKIYAGYYNTSLELTDIQLMKKGEDYTIEYNSAYTSFNLKILNTATRLATNGKPATYSISYSTTSPANGTDVVNELSMTGGEQEITTRTDRTTTINRVTRSTKITTGGTIQLETGYRITLYKQDAETGNLLNGAEFEITSPSGDKETVKIEKDGVAQSKVYSAEEAKKGKFTIVEIKAPKGYVLDQTPIEVSLGENGIIRTIKNSKEKLSIPVTKQWNDNNNQDGKRPEDITVQLYANGQVVDGQTMTLNNENNWTAQFENLAKIDDDGKEIAYTVKEISVDGYTSNISGDTENGYTITNTYSPGKVEISGTKTWSDNNNQDGKRPDSITINLLADGEKVDSQTVTANNNWAYNFTDLAKYKDGKEVVYTVTEDPVSDYTTTINGYNITNSYTPATTSISGTKTWSDNDNQDGKRPDSITINLLANGKKVDSQKVSAATNWAYHFGNLPQYENGQVITYTVNEDAVDSYSTSINGYNITNSYTPETVDVKATKNWDDNDNQDGKRPTSITVNLLADGEKINSQKVVAAADGTWTASFTNLPKYKDGKEIKYTVTEEAVEEYETIINDFTITNKYIPKAIEYKVTKVWKDNNNQDGKRPSAITVQLYKSVGEAKPTPIAGKTLTLTADNQTNDNTWEASFTNLPQFEKGQEIAYSVQEVDTPEGYTASVEGQTITNSHDPETIIISGQKKWDDNDNQDGKRTTTVKVQILKGEEVVDEIEVSEASNWAFTSKALPKYENGKEITYTVKEVAVTAYETKIEKDDDGQYTITNTHTPEKINLSGQKVWNDNNNQDGIRPENITVELLANGEATGQTATASEATAWQYSFENLDRYKDSKEVSYSVKEVNIPEGYKAEVDGMNVTNNHVPETITLTGQKTWDDNNDQDGIRPQSITLHLLANGVDTGLTTTATADTNWTYSFKDIAKYQNGKEVTYTVSEDTVGGYTSVVDGMNVTNTHTPTTPTTPKSDKPNSPSSNQKNKPKQSSLLPATGDKGDLTLTILGVFLIVAIISGMIYYKIRKM |
| 49 | MQDGIQEVEHKPSETTPPTSSGDKVTITSYQKGWKHFLPKTGQAKSILTIAGFLLIVLAVVGLICYRRRQTKD |
| 50 | MAVKGVKDVMNKAKVMTATATLVAGLGVASHVNADEVDLSSTIQNSEAQANQTESNVTKADVNAAKADLDTANQAVSHQEQVVSDAATAADEAQSTYNQAQQSTNEAQNFVDQATPENIASAQDDVNSAQEQVSQADNAERVAENTVAQAQEAVNNQTEVVNSAQTAVDAAQTAVDAAQKDVNDKQAILDGTGQAEIIANRDKAQDDVNLAQNQVAQAESDLTKAQEADANRQQVIDNAQKAIDAANQTVTSSKSDLAAKTAQANQTQATEDAKQADVATAQKDVNDKQAILDGTGQKAILDEAEAAKNDEASKQANLSAAQSALKQAQEADANRQQSIDNAQKAVDESNQIVSTTKSDLDAKAAQAQQAEQALADAQSVYKTAKNDYKAINTITLSEDYIRLLKQSYTANTLKERQQALEELKLKNNDLKPEYVFKSNSNDDKHIVDLNNLSLNQLTELSLFASDLVNQIRQQVGSASTVVTDSSVGFAKKVTENYILDNWDMAKGHDAFAVNNAAKYYGLSTSTEQWEKQGLQYYENWAGLSRNSQSTMSDLKSDIYDAIYRFMFDGDEYDHASSIAGIRATNTTTVSYLGVGFTSLSTSRFDGVHFLLVGDNYVKNAQNNNFSTIVIENLKTPEKVTTTYNTAKADLEAKTATNNSAQSALSAAQNSYNTAIAANTKAQQVLDEAQSVVIQTPTAKANLASAETALTKAQERLTKANKAVEDLNADIKAKQAALTSAKATLEAAQSALAKAQAENSVMQEAKATAQTAYDNAVSNLSSASATLTQAQLVAIQMPTAKANLASAQATLKQAQENLVKAQKAVDDLSADIAVKQANLASAKQVLSTKQVTLVTKQATLATEQNRLASLQNSLATAQANVLKAKENVVTAKDNLTKVQTYLTRLQNAPVLLAQAQQQEAIAKANMLAALDTLEAELLKLKDLQVKQAAAQAVYDTTSKAYQTMLDAQEKQRLQDEYNAIIAQGKTPVAIVDETGKIVSYQAEDPQTTIQSSSVESTAVKAQVKPVEEVSVVMASALPMTGEHSSVLAVLFGCLMTIWGLVGVRRKNN |
| 51 | MLQSNLKGHGYLRKSKAYGLISGLALGAVAVTTTAVSADETTEMVNTQPDITITSESNATNLSAPQADNSEAHDDLATQAGSQSGDMTNSVTSDTLNQAVTEAEQAGVDVTTGDTVTHDSLADAQADLNNQQAAVDQATETQKAVDNQTATATETAKQTGVEVTTSDSKTYTSTVDAKADAKAQTDNLNQVTTAQKQVNEQLPKAIESATQSGVKVTVKAGAKTSDAKKALDELSKQVVSLKQAKDTQTAITESMAQALNEAKKIGVTVNTANAKTYDDLAQALADAQAQVAKLTEFKETQASVDEAVKQSVDNAKTSGVTVSTGETKTYTSVEEAKADSAKQTASLDKAAETQKEADQVISDLTKQAQDAGLDLTKGASKSYTSVEEAKQDLAKQEQALKDAQAAKTKAETTNLANKQAAEEAQAAANKAIDDAVSKATASGTTVTNTGTIETTVAEAQKIAQAQVDKINEVVAKNEQIQKENADKKTAYETQKAHIDAINKAEFDKYKLTYTGDYAKDKATADKFNADEIERVKHQTKEFNDKFNALNNQNALNPTDGWVANVGANGFTQSGTSQFKKAQTVGEYELIATGSLEPGKSTVYIKKADGSNVDKNHIIKNINWGNVSPEGPSLEGNIGLDWAGAYHYNDNGTTTQIWKGKISNWYRIPQAITMLDGSVHDAYVLFHKDNSGLNSPADEVVFWNQDGAINVIDGYRMGVNDQSDAVRTVIRVDAPENEQNYLWISLLGDFDMGQYLQAENNVVVLGLGGGFATNSGIGGNPIAVDENLGGNYGINYGFNALDGFNSAPDGVVLIAQYGREYSTVIRNTPGGNASAVARADFGSDARVDTIPSPEPPKSITISNIDKPTYKDPLTTSVNTVHITPNTVSIPKAVSLHDVNINASTHDIHLETATNPITLETTVNTPALAANVHDITLKTGIHPVQVTQKPTNTKAVINNDKVDINGKTVAKGSTVQWLLQNHKLLAGRKEITAVVMNDPFPAGFDVDSEATAEANADYYTLKTTDDGSYQLVGTAKLLSVLNTNRNQDVEVPAFTFVGKPLNDKAHYDNTFETIYSTASGYYKVVSNIPSIDTPDNPNPTKTVTDKAGSDIDGKTVFDKEVTFHLITDYSVYVNTIVDIDTMAKSAGIYDDTDDKTVNPDITKATVTDSEGNDITAKGTFYDIEAGQPIPAEIQIFLDGAHLTPDGEFVVWIPNDLKDYYQNYVLTGNNVTVNLPVEIIAQPGETAENTFVQVEFGNGYQSNLVFVKVPDVSPEKHAISKDGTVLDGQEVGLNQVFNYKLDGVTVQKDHDTLWQYDGKDKLDLEHDRYTGNWKGVITGTEYTAKEDMTLPYDVITEDGTVLKAGDTIAAGTSYRFIFEFNQDTNDDFINQIVKVTWDAEKGEWAYSIDKDFLNSLGVEGTFDADFWIEVERIKDGEVENTFINIINGRELVAKVTTHTPTPETPAEPEQPAKPIEEVPVVKAAALPMTGDETTPMSVLATLIGLMMTVGAVFGLRKKENHH |
| 52 | MKKIKAVALFSTAILAIASTGVYADELISDGTTSPSTEQVTPSTDTSSEVVIPDDGSTTPSTDTGSTTEPTVPVDPTTPSTGDNTGDNGSTNPGTSEDEPSNPSSEITEPETPAEPEQPSTEQPGEVEVPTTDGGTATVTPDTSVPTNNPNITAETAQNAGASQVGTTSTVTGQVVRDVTHSNPVTLYNGASLVDIRDGLVTLSSGEKVTPESVGVTANADGTYTAKTIQGDMVTLPHTGEKNTTILSVLGAFILSVLGFSLKKRNHQES |
| 53 | MNQKETLNGHGFFRKSKAYGLICGIALGVAFLGGKVNADEIVVPDGTDVPVVTEASQTIVEPASDDLTTAITNAEEAGVTVSQDTSETVVNQEEAQADYASQAESLAAVTVAQEQINAENAQITADNQALNEAYDNAKAQAESTNQAVSEAQSTYGATVTETTVNYGDGTSTTDYQAGQVQAENVAKANSQAISDYLAKKAEVDAYNAQVKAREDALKSNNIASDEANYLYVIGEFDTNATGLDYYKNITVVTLDPNAKAAQTLGWQDNTTISNVNGVTVTSHDTANDPAIYGTTSDFLYKITEATAGDTFTLNNIGTATDGTNINAIVTITKASSLTDQEDSWFVIGKTADNGIAVDYWNYDNLGLSFQFVDDSGNAIKLVVASVVGDVDNDQTSKIEFDGNTLNYVNPDGSGLIANADKSLTGLGFAVDGYQQAPQGTYLMVGSSTTVNYTHTSDDNVVDGNGNIVNYIEFDLFGTTSMVTTEEFKYLPDPTLTLTSVTLPTSPVETPLKDNLTATYHLNEYDVALTTVKDVLNSPGISIDGGELQIGETGHYTLEGAKVLANGKETLVKYDFEDYLDVEHDQYQGYSIYAFVPITLKDGTVIQSGEDLKAYAQAVYDDVTGHFYVVSLNSDFLAQVAKESDFQAKVDIEFVRIASGDVYNNFTNHLAFADSEGNVTEVPVPSNEVVTYTPEQPVEETPETPITPTSETPATPEDVPVVQESALPLTGDETTPLNIIASLFGVLMFVLGIVGIRKRNEN |

**Table S4. List of putative lipoproteins found in UCN34 present or absent in cancer associated- *SGG* (red), non cancer- associated *SGG* (green), non human *SGG* isolate (blue)and *SGM* (orange)**

| Name in UCN34 | LipoBox motif | Annotation | Size (AA) | *SGG* | | | | | | | | | | | | | | | | | | | | | *SGM* | | |
|---|---|---|---|---|---|---|---|---|---|---|---|---|---|---|---|---|---|---|---|---|---|---|---|---|---|---|---|
| Gallo | | | | UCN34 | TX20005 | 20 | 30 | 37 | 40 | 42 | 44 | SGG33 | SGG34 | SGG41 | SGG23 | SGG24 | SGG25 | SGG26 | SGG27 | SGG47 | SGG49 | SGG50 | SGG52 | DSM16831 | ACA-DC198 | 679 | CIP 105683T |
| _0116 | LSAC | multiple sugar ABC transporter | 435 | + | + | + | + | + | + | + | + | + | + | + | + | + | + | + | + | + | + | + | + | + | Abs | Abs | Abs |
| _0122 | LfGC | Trp / Tyr ABC transporter | 658 | + | + | + | + | + | + | + | + | + | + | + | + | + | + | + | + | + | + | + | + | + | + | + | + |
| _0163 | LVGC | raffinose/stachyose/melibioseAB[illegible] | 430 | + | + | + | + | + | + | + | + | + | + | + | + | + | + | + | + | + | + | + | + | + | + | + | + |
| _0192 | LTAC | raffinose/stachyose/melibiose AB[illegible] | 423 | + | + | + | + | + | + | + | + | + | + | + | + | + | + | + | + | + | + | + | + | + | + | + | + |
| _0324 | LAAC | oligopeptide ABC transporter | 551 | + | + | + | + | + | + | + | + | + | + | + | + | + | + | + | + | + | + | + | + | + | + | + | + |
| _0414 | LAAC | L-cystine ABC transporter | 274 | + | + | + | + | + | + | + | + | + | + | + | + | + | + | + | + | + | + | + | + | + | + | + | + |
| _0624 | LGAC | DUF6287-containing protein | 207 | + | + | + | + | + | + | + | + | + | + | + | + | + | + | + | + | + | + | + | + | + | Abs | Abs | Abs |
| _0696 | LTAC | hypothetical serine rich lipoprotein | 168 | + | + | + | + | + | + | + | + | + | + | + | + | + | + | + | + | + | + | + | + | + | Abs | Abs | Abs |
| _0751 | LTAC | Flg-new containing protein | 258 | + | Abs | + | + | + | + | + | + | + | + | + | + | + | + | + | + | + | + | + | + | Abs | Abs | Abs | Abs |
| _0874 | LgAC | ferrichrome ABC transporter | 353 | + | + | + | + | + | + | + | + | + | + | + | + | + | + | + | + | + | + | + | + | + | + | + | + |
| _0883 | LSAC | 7,8-dihydro-8-oxoguanine-triphosphatase (MutT) | 158 | + | + | + | + | Abs | + | + | + | + | + | + | + | + | + | Abs | + | + | Abs | + | Abs | + | Abs | Abs | Abs |
| _0933 | LTAC | tannase (tanA) | 596 | + | + | + | + | + | + | + | + | + | + | + | + | + | + | + | + | + | + | + | + | + | Abs | Abs | Abs |
| _1000 | LAAC | amino acid ABC transporter | 280 | + | + | + | + | + | + | + | + | + | + | + | + | + | + | + | + | + | + | + | + | + | + | + | + |
| _1117 | LgAC | carbohydrate-binding domain-containing protein | 410 | + | + | + | + | + | + | + | + | + | + | + | + | + | + | + | + | + | + | + | + | + | Abs | Abs | Abs |
| _1136 | LgAC | BMP family protein ABC transporter | 354 | + | + | + | + | + | + | + | + | + | + | + | + | + | + | + | + | + | + | + | + | + | + | + | + |
| _1149 | LSGC | phosphate ABC transporter (PstS) | 288 | + | + | + | + | + | + | + | + | + | + | + | + | + | + | + | + | + | + | + | + | + | + | + | + |
| _1234 | LVGC | polar amino acid ABC transporter | 292 | + | + | + | + | + | + | + | + | + | + | + | + | + | + | + | + | + | + | + | + | + | + | + | + |
| _1245 | VVGC | hypothetical protein | 154 | + | + | + | + | Abs | + | + | + | + | + | + | + | + | + | + | + | + | + | + | + | + | Abs | Abs | Abs |
| _1367 | LAAC | DUF6287 domain-containing protein | 193 | + | + | + | + | + | + | + | + | + | + | + | + | + | + | + | + | + | + | + | + | + | + | + | + |
| _1373 | VSAC | glucosyltransferase domain-containing protein | 489 | + | + | + | + | + | + | + | + | + | + | + | + | + | + | Abs | + | + | + | + | Abs | + | Abs | + | Abs |
| _1392 | LAAC | arginine/lysine/histidine ABC transporter | 273 | + | + | + | + | + | + | + | + | + | + | + | + | + | + | + | + | + | + | + | + | + | + | + | + |
| _1394 | LSAC | arginine/lysine/histidine ABC transporter | 271 | + | + | + | + | + | + | + | + | + | + | + | + | + | + | + | + | + | + | + | + | + | + | + | + |
| _1395 | LSAC | amino acid ABC transporter | 281 | + | + | + | + | + | + | + | + | + | + | + | + | + | Abs | + | + | + | + | + | + | + | + | + | + |
| _1399 | LVAC | maltose/maltodextrin ABC transporter | 418 | + | + | + | + | + | + | + | + | + | + | + | + | + | + | + | + | + | + | + | + | + | + | + | + |
| _1412 | LAAC | oligopeptide ABC transporter | 548 | + | + | + | + | + | + | + | + | + | + | + | + | + | + | + | + | + | + | + | + | + | + | + | + |
| _1413 | LAAC | oligopeptide ABC transporter | 549 | + | + | + | + | + | + | + | + | + | + | + | + | + | + | + | + | + | + | + | + | + | + | + | + |
| _1586 | LAAC | LPAM-1 containing protein | 588 | + | + | + | + | + | + | + | + | + | + | + | + | + | + | + | + | + | + | + | + | + | Abs | Abs | Abs |
| _1601 | LAAC | FIVAR domain containing protein | 427 | + | Abs | + | + | + | + | + | + | + | + | + | + | Abs | + | + | + | Abs | + | Abs | Abs | + | Abs | Abs | Abs |
| _1650 | VrSC | New glue mucin-like protein | 188 | + | Abs | + | + | Abs | Abs | + | + | Abs | Abs | Abs | + | + | + | + | + | + | + | + | Abs | Abs | Abs | Abs | Abs |
| _1710 | LVGC | Tpr ring domain containing protein | 127 | + | Abs | + | + | + | + | + | + | + | + | Abs | + | + | + | Abs | + | + | + | Abs | Abs | Abs | Abs | Abs | Abs |
| _1717 | LSGC | peptidyl-prolyl cis-trans isomerase A | 278 | + | + | + | + | Abs | + | + | + | + | + | + | + | + | + | + | + | + | + | + | + | + | + | + | + |
| _1760 | LAAC | branched-chain amino acid ABC transporter | 393 | + | + | + | + | + | + | + | + | + | + | + | + | + | + | + | + | + | + | + | + | + | + | + | + |
| _1772 | LVAC | ferric hydroxamate/ heme ABC transporter | 310 | + | + | + | + | + | + | + | + | + | + | + | + | + | + | + | + | + | + | + | + | + | + | + | + |
| _1778 | LSGC | DUF6287 domain-containing protein | 325 | + | Abs | + | + | + | + | + | + | + | + | + | + | Abs | + | Abs | Abs | Abs | Abs | Abs | Abs | Abs | + | + | + |
| _1814 | LTGC | YidC/Oxa1 family membrane protein insertase | 306 | + | + | + | + | + | + | + | + | + | + | + | + | + | + | + | + | + | + | + | + | + | + | + | + |
| _1844 | LAAC | D-methionine ABC transporter | 299 | + | + | + | + | + | + | + | + | + | + | + | + | + | + | + | + | + | + | + | + | + | + | + | + |
| _1845 | LAAC | L-cystine ABC transporter | 280 | + | + | + | + | + | + | + | + | + | + | + | + | + | + | + | + | + | + | + | + | + | + | + | + |
| _1907 | LAGC | murein lipoprotein | 149 | + | + | + | + | + | + | + | + | + | + | + | + | + | + | + | + | + | + | + | + | + | Abs | Abs | Abs |
| _1966 | LTAC | phospate ABC transporter | 289 | + | + | + | + | + | + | + | + | + | + | + | + | + | + | + | + | + | + | + | + | + | Abs | Abs | Abs |
| _2047 | LgAC | iron/zinc/manganese/copper ABC transporter | 308 | + | + | + | + | + | + | + | + | + | + | + | + | + | + | + | + | + | + | + | + | + | Abs | Abs | Abs |
| _2078 | LAAC | YidC/Oxa1 family membrane protein insertase | 271 | + | + | + | + | + | + | + | + | + | + | + | + | + | + | + | + | + | + | + | + | + | + | + | + |
| _2130 | LTGC | D-methionine ABC transporter | 277 | + | + | + | + | + | + | + | + | + | + | + | + | + | + | + | + | + | + | + | + | + | Abs | Abs | Abs |
| _2214 | LVGC | hypothetical protein | 304 | + | Abs | + | + | Abs | + | + | Abs | + | + | Abs | + | Abs | + | Abs | Abs | Abs | Abs | Abs | Abs | Abs | Abs | Abs | Abs |

**Table S5. Predicted virulence factors detected in cancer-, non cancer-associated SGG, non human SGG, and SGM isolates by using the Virulence Factor Database (VFDB)**

| | | | ***S.gallolyticus* sp. *gallolyticus*** | | | | | | | | | | | | | | | | | | | | | | | | ***S. gallolyticus* sp. *macedonicus*** | | | |
|---|---|---|---|---|---|---|---|---|---|---|---|---|---|---|---|---|---|---|---|---|---|---|---|---|---|---|---|---|---|
| | | | **CRC group** | | | | | | | | | | | | **Non cancer group** | | | | | | | | | | **Non human** | | | | |
| **VFclass** | **Virulence factors** | **Related genes** | **UCN34** | **TX20005** | **SGG 20** | **SGG30** | **SGG37** | **SGG40** | **SGG42** | **SGG44** | **SGG 33** | **SGG 34** | **SGG 41** | **Prevalence (%)** | **SGG 23** | **SGG 24** | **SGG 25** | **SGG 26** | **SGG 27** | **SGG 47** | **SGG 49** | **SGG50** | **SGG 52** | **Prevalence (%)** | **DSM16831** | **CIP_105683T** | **ACA-DC198** | **SGM_679** | **Prevalence (%)** |
| Adherence | Agglutinin receptor | Undetermined | - | - | - | - | - | - | - | - | - | + | - | 9 | - | + | - | - | - | - | - | - | - | 11.1 | + | + | + | + | 100 |
| | Fibronectin-binding protein | fbp54 | + | + | + | + | + | + | + | + | + | + | + | 100 | + | + | + | + | + | + | + | + | + | 100 | + | + | + | + | 100 |
| | Streptococcal glucosyltransferases | gtfD | + | + | + | + | + | + | + | + | + | + | + | 100 | + | - | + | + | + | + | + | + | + | 88.9 | + | - | - | - | 0 |
| | | gtfG | - | + | + | + | + | + | + | + | + | + | + | 90.9 | + | + | + | - | + | + | + | + | - | 77.8 | - | - | - | - | 0 |
| | Streptococcal lipoprotein rotamase A | slrA | + | + | + | + | + | + | + | + | + | + | + | 100 | + | + | + | + | + | + | + | + | + | 100 | + | + | + | + | 100 |
| | Streptococcal plasmin receptor/GAPDH | plr/gapA | + | + | + | + | + | + | + | + | + | + | + | 100 | + | + | + | + | + | + | + | + | + | 100 | + | + | + | + | 100 |
| Enzyme | Streptococcal enolase | eno | + | + | + | + | + | + | + | + | + | + | + | 100 | + | + | + | + | + | + | + | + | + | 100 | + | + | + | + | 100 |
| Immune evasion | Capsule biosynthesis | Undetermined | + | + | + | + | + | + | + | + | + | + | + | 100 | + | + | + | + | + | + | + | + | + | 100 | + | + | + | + | 100 |
| | Polysaccharide capsule(Bacillus) | galE3 | + | + | + | + | + | + | + | + | - | + | + | 90.9 | + | + | + | + | + | + | - | + | + | 88.9 | + | - | - | - | 0 |
| Manganese uptake | Pneumococcal surface antigen A / Metal binding protein SloC | psaA | + | + | + | + | + | + | + | + | + | + | + | 100 | + | + | + | + | + | + | + | + | + | `100 | + | - | - | - | 0 |
| Protease | C3-degrading protease | cppA | + | + | + | + | + | + | + | + | + | + | + | 100 | + | + | + | + | + | + | + | + | + | 100 | + | + | + | + | 100 |
| | C5a peptidase | scpA/scpB | + | + | + | + | + | - | + | - | + | + | + | 81.8 | + | + | + | + | + | + | + | + | + | 100 | + | - | - | - | 0 |
| | Serine protease | htrA/degP | + | + | + | + | + | + | + | + | + | + | + | 100 | + | + | + | + | + | + | + | + | + | 100 | + | + | + | + | 100 |
| | Trigger factor | tig/ropA | + | + | + | + | + | + | + | + | + | + | + | 100 | + | + | + | + | + | + | + | + | + | 100 | + | + | + | + | 100 |
| Toxin | Cytolysin (Enterococcus) | cylR2 | - | - | + | - | - | + | - | + | - | + | + | 45.5 | + | + | + | - | + | + | + | + | + | 88.9 | - | - | - | - | 0 |
| Bile resistance | Bile-salt hydrolase(Listeria) | bsh | + | + | + | + | + | + | + | + | + | + | + | 100 | + | + | + | + | + | + | + | + | + | 100 | + | - | - | - | 0 |
| Lipid and fatty acid metabolism | Pantothenate synthesis (Mycobacterium) | panD | + | + | + | + | + | + | - | + | - | + | + | 81.8 | - | + | + | + | + | + | + | + | + | 88.9 | - | - | - | - | 0 |
| Secretion system | Type VII secretion system | esxA | T7SS$_{UCN34}$-type | T7SS$_{UTX20005}$-type | T7SS$_{UCN34}$-type | T7SS$_{UCN34}$-type | T7SS$_{UCN34}$-type | T7SS$_{UCN34}$-type | T7SS$_{UCN34}$-type | T7SS$_{UTX20005}$-type | T7SS$_{UCN34}$-type | T7SS$_{UCN34}$-type | T7SS$_{UCN34}$-type | | T7SS$_{UCN34}$-type | T7SS$_{UTX20005}$-type | T7SS$_{UCN34}$-type | T7SS$_{UCN34}$-type | T7SS$_{UTX20005}$-type | T7SS$_{UTX20005}$-type | T7SS$_{UCN34}$-type | T7SS$_{UCN34}$-type | T7SS$_{UCN34}$-type | | T7SS$_{UCN34}$-type | - | - | - | |
